# A new network representation of the metabolism to detect chemical transformation modules

**DOI:** 10.1186/s12859-015-0809-4

**Published:** 2015-11-14

**Authors:** Maria Sorokina, Claudine Medigue, David Vallenet

**Affiliations:** 1 Direction des Sciences du Vivant, Commissariat à l’Energie Atomique et aux Energies Alternatives (CEA), Institut de Génomique, Genoscope, Laboratoire d’Analyses Bioinformatiques pour la Génomique et le Métabolisme, 2 rue Gaston Crémieux, Evry, 91057 France; 2 CNRS-UMR8030, 2 rue Gaston Crémieux, Evry, 91057 France; 3 UEVE, Université d’Evry Val d’Essonne, Boulevard François Mitterrand, Evry, 91057 France

**Keywords:** Metabolic network, Reaction signatures, Graph reduction, Pathway conservation, Chemical transformation modules

## Abstract

**Background:**

Metabolism is generally modeled by directed networks where nodes represent reactions and/or metabolites. In order to explore metabolic pathway conservation and divergence among organisms, previous studies were based on graph alignment to find similar pathways. Few years ago, the concept of chemical transformation modules, also called reaction modules, was introduced and correspond to sequences of chemical transformations which are conserved in metabolism. We propose here a novel graph representation of the metabolic network where reactions sharing a same chemical transformation type are grouped in Reaction Molecular Signatures (RMS).

**Results:**

RMS were automatically computed for all reactions and encode changes in atoms and bonds. A reaction network containing all available metabolic knowledge was then reduced by an aggregation of reaction nodes and edges to obtain a RMS network. Paths in this network were explored and a substantial number of conserved chemical transformation modules was detected. Furthermore, this graph-based formalism allows us to define several path scores reflecting different biological conservation meanings. These scores are significantly higher for paths corresponding to known metabolic pathways and were used conjointly to build association rules that should predict metabolic pathway types like biosynthesis or degradation.

**Conclusions:**

This representation of metabolism in a RMS network offers new insights to capture relevant metabolic contexts. Furthermore, along with genomic context methods, it should improve the detection of gene clusters corresponding to new metabolic pathways.

**Electronic supplementary material:**

The online version of this article (doi:10.1186/s12859-015-0809-4) contains supplementary material, which is available to authorized users.

## Background

In bioinformatics, metabolism is generally modeled by directed networks where nodes represent reactions and/or metabolites and edges the product/substrate exchanges between reactions [1]. Metabolic network reconstruction of a given organism generally starts with its genome annotation that predicts enzymatic activities from coding sequences and, therefore, the corresponding reactions and metabolites of the network. However, two main bottlenecks limit today this reconstruction by homology: the difficulty in associating correct functions to genes and the lack of experimental characterization of enzyme activities for which proteins are sometimes unknown, *i.e.* orphan enzymes [2].

Subgraphs of these networks are often used to represent metabolic pathways that group sets of connected reactions involved in a same biological process. Several hypotheses on the origin and evolution of metabolic pathways have been proposed, including patchwork evolution by enzyme recruitment in new metabolic pathways [3, 4], retrograde synthesis which postulates that metabolic pathways are constructed starting from the final metabolite [5], and the theory on metabolic pathway duplication [6]. Despite their differences, these hypotheses agree about the importance of enzyme promiscuity in the evolution of metabolic pathways, *i.e.* the capacity of enzymes to catalyze one or several types of reactions on more or less different substrates. A recent study in *Escherichia coli* successfully brings out this enzyme capacity to adapt themselves to new substrates [7].

In order to explore metabolic pathway conservation and divergence among organisms, previous studies were based on pathway alignment to find similar pathways within or between organisms using the Enzyme Commission (EC) numbers to define reaction similarities [8–11]. Due to limitations of the EC classification, the notion of reaction similarity for pathway alignment was improved using metabolite similarity [12] or substructure changes [13]. Another approach, that does not require predefined pathways, was based on the detection of motifs in a reaction network [14]. Few years ago, the concept of chemical transformation modules, also called reaction modules, was introduced by Muto *et al.* [15]. They correspond to sequences of chemical transformations which are conserved in metabolism. These modules capture the chemical logic of pathways that may correspond or not to conserved sets of enzymes. Muto *et al.* made a systematic analysis of the conservation of reaction modules by aligning metabolic pathways from KEGG [16] and used RClass (Reaction Class) [17] to group reactions having same patterns of chemical transformations. The same year, Barba *et al.* [18] published a study on the modularity of the purine and pyrimidine metabolism, which presents chemical reaction similarities, and also enriched the reaction module definition with the notion of enzyme homology.

In the present work, we propose a different formalism for the detection of reaction modules, although we use the same definition of modules as Muto *et al.* [15]. Instead of using pathway alignment, we adopt an innovative graph representation of the metabolism where the reaction network is reduced in a Reaction Molecular Signature (RMS) network. For that, RMS are automatically computed for all reactions and encode changes in atoms and bonds as described in [19]. Thereby, reactions sharing a same signature are grouped together. Paths in the RMS network are then explored to detect conserved modules. Furthermore, this graph-based formalism allows us to define several path scores reflecting different biological conservation meanings. These scores are finally analyzed for all possible paths in the network and for known metabolic ones and used to build association rules that should predict metabolic pathway types like metabolite biosynthesis or degradation.

## Methods

### Reaction network

Metabolic data was extracted from MetaCyc public database version 19.0 [20]. MetaCyc contains a large collection of curated metabolic pathways from all domains of life. In addition, metabolites, reactions, enzymes and genes are also listed. Metabolic pathways described in MetaCyc are generally short (4.3 reactions on average) and have been experimentally elucidated in at least one organism. A metabolic network was reconstructed using MetaCyc reactions as nodes. We linked two reactions by a directed edge when the product of one reaction is the substrate of the other one. However, to avoid the high connectivity problems that are common when building such metabolic networks, we limited shared compounds to “main compounds”, *i.e.* metabolites deemed biologically relevant to both reactions in at least one metabolic pathway. Only reactions that belong to a metabolic pathway were taken into account, as only these ones have distinction between main metabolites and co-substrates supporting the reaction such as water, ATP or NAD. Transport reactions, for which translocated substrate remains unchanged, were excluded from the network construction and from further analysis, *e.g.* ABC transporter ATPase reactions corresponding to 3.6.3.- EC class.

### Reaction molecular signatures

Reaction Molecular Signatures (RMS) were computed for all MetaCyc reactions, belonging or not to a metabolic pathway, as described in [19]. These signatures encode changes in atoms and bonds where the reaction is taking place. First, structures of all molecules involved in a reaction were downloaded from MetaCyc website in MDL Molfile format. Using ChemAxon MolConvert software [21], all molecules were standardized by adding implicit hydrogen atoms and applying aromatization when needed. Stereo signature molecular descriptors [22] were then computed for heights 1 and 2 with the MolSig software (http://molsig.sourceforge.net). These molecular signatures are encoded using SMILES-like strings [23] and the height parameter corresponds to a distance for the inclusion of neighbour atoms and bonds up from a given atom. Second, corresponding RMS were generated for each molecular signature height by calculating the difference between the signatures of the products and of the substrates. To obtain correct RMS, reaction equations have to be balanced with explicit compounds for which Molfile structures are available. It should be noticed that (i) for a given height, a reaction has only one RMS signature (ii) reactions sharing a same RMS have similar chemical transformations (iii) the higher the height value is more the signature is precise. RMS of height 1 (RMS-H1) capture the reaction center with atom and bond changes. To compute RMS of height 2 (RMS-H2), RMS-H1 were partitioned in sub-groups having similar signatures at height 2. Distances between signatures were computed using an approximate string matching algorithm [24]. Then, a hierarchical clustering was build on these distances using the Ward algorithm [25] and the tree was cut at a cophenetic distance threshold of 90. To deal with reaction directionality, RMS having strictly opposite signatures were merged in a single entry. Higher values of the height parameter were not used because they lead to too precise signatures with many describing only one reaction. The RMS classification of reactions is available in Additional file 1 and the source code for the RMS computation was deposited in GitHub (https://github.com/mSorok/createRMS.git). The RMS method has been chosen in this work as it guarantees that all reactions described by the same signature perform the same chemical transformation, making manual post-process unnecessary.

### RMS networks

**Fig. 1:**
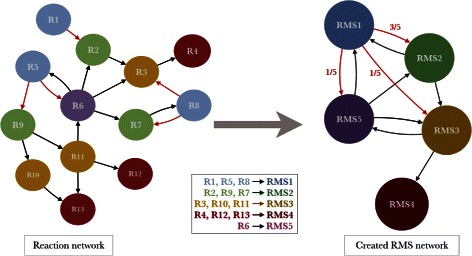
Reaction network to Reaction Molecular Signature network. This figure presents a toy example of the reduction of a reaction network in a RMS network. Reactions sharing a same reaction signature (same node color in the figure) are grouped in a single RMS node. Directed edges of the reaction network are also merged in the RMS network. Red edges illustrate the computation of Markov transition probabilities *P* *r*(*R* *M* *S*_2_∣*R* *M* *S*_1_), *P* *r*(*R* *M* *S*_3_∣*R* *M* *S*_1_) and *P* *r*(*R* *M* *S*_5_∣*R* *M* *S*_1_). They correspond to the proportion of reaction edges, among the five outgoing edges of RMS1 reactions (blue nodes), connecting RMS1 to RMS2, RMS3 and RMS5

The reaction network was reduced in a directed network of chemical transformations represented by RMS. As shown in Fig. 1, reactions signed by the same RMS are grouped in a single node. Two RMS are connected by a directed edge in the RMS network if there is at least one edge in the original reaction network linking reactions signed by the corresponding source and target RMS. For computational complexity reasons and the lack of explicit representation of repeated reactions in pathway databases, edges are not created if source and target RMS are identical (*i.e.* self-loops are avoided). This transformation was made for the two RMS heights and we obtained two networks called RMS-H1 and RMS-H2 networks. Furthermore, this graph reduction, which aggregates reaction nodes and edges, allowed us to define Markov chains transition probabilities of order 1 between connected RMS. $\text {Pr} \left (RMS_{j} \mid RMS_{i} \right)$ is calculated as the ratio of the number of outgoing reaction edges linking *R* *M* *S*_*i*_ to *R* *M* *S*_*j*_ among the total number of outgoing edges from reactions signed by *R* *M* *S*_*i*_.

### RMS node weighting

Several weights, reflecting different biological conservation meanings, have been computed on nodes of the RMS networks. The first weight, *wRea*, corresponds to the number of MetaCyc reactions associated to a given RMS, whether they are present or not in the initial reaction network. It gives a quantitative measure of the diversity of reactions represented by a RMS.

A second weight, *wPageRank*, is computed using PageRank algorithm [26] implemented in the Jung 2.0 Java library [27]. This topological weight is based on a network architecture exploration in order to locate influential nodes in the RMS network with the assumption that most important chemical transformations are likely to have more incoming links from other transformations.

The last weight, *wProt*, is an estimation of the number of proteins associated to a given RMS. Known protein/reaction associations were extracted directly from MetaCyc and from Swiss-Prot using EC numbers [28]. These associations were used to compute two ratios corresponding to the number of known proteins with the same Pfam domain composition [29] and associated to a given RMS $N_{p}(p \in RMS_{i} \:\bigcap \: p \in Dom_{j})$ divided by the total number of known proteins having the domains *N*_*p*_(*p*∈*D* *o* *m*_*j*_), for *d2r* ratio, or by the total number of known proteins associated to the RMS *N*_*p*_(*p*∈*R* *M* *S*_*i*_), for *r2d* ratio.

(1) $$\begin{array}{@{}rcl@{}} d2r(RMS_{i},Dom_{j})=\frac{N_{p}(\,p \in RMS_{i} \bigcap p \in Dom_{j})}{N_{p}(\,p \in Dom_{j})} \end{array} $$

(2) $$\begin{array}{@{}rcl@{}} r2d(RMS_{i},Dom_{j})=\frac{N_{p}(\,p \in RMS_{i} \bigcap p \in Dom_{j})}{N_{p}(\,p \in RMS_{i})} \end{array} $$

Next, the association score, *s* *c* *o* *r* *e*(*Dom,RMS*), was computed as the harmonic mean of *d2r* and *r2d* values. This score represents a trade-off between sensitivity and specificity to associate protein domains to chemical transformations and tends to be very low when domains or RMS are very frequent.

(3) $$ score(Dom_j,RMS_i) = \frac{2 \times d2r_{i,j} \times r2d_{i,j}}{d2r_{i,j} + r2d_{i,j}}  $$

Finally, *wProt* is, for each protein domain associated to the given RMS, the geometric mean of the total number of UniProt proteins associated to a domain multiplied by the *s* *c* *o* *r* *e*(*Dom,RMS*). Only proteins from UniProt reference proteomes [28] (version 2015_04 with 2,424 reference proteomes) were considered to provide broad coverage of the tree of life while reducing taxonomic over-representation.

(4) $$  wProt(RMS)=\sqrt[n]{\prod_{j=1}^{n}N_{p}(\,p \in Dom_{j}) \times score(Dom_{j},RMS)}  $$

This weight gives a quantitative measure of the diversity of enzymes associated to a RMS. High value of *wProt* may indicate that the chemical transformation is widely represented among organisms and/or that many enzymes catalyze this transformation because of many gene duplications or many enzyme families.

### RMS path enumeration and scoring

An enumeration of all paths of length 1 (one edge and two RMS nodes) to 4 (four edges and five nodes) was made in both RMS networks using the Grph Java library [30]. In this path enumeration, loops were not allowed (*i.e.* a node cannot be found more than once in a path). To make them comparable, metabolic pathways from MetaCyc were translated in overlapping RMS paths of the same length. In addition, a Pathway Conservation Index (*PCI*) was computed for each RMS path and represents the number of distinct corresponding reaction paths that are present in at least one MetaCyc pathway.

According to previously defined RMS weights, path conservation scores, named *scoreRea*, *scorePageRank* and *scoreProt*, were calculated as the geometrical means of path node weights multiplied by their probability of transition to the next node of the path. As an illustration, the formula of *scoreRea* is given in which *R* *M* *S*_*i*_ and *R* *M* *S*_*i*+1_ are two consecutive nodes and *n* is the path length.

(5) $$\begin{array}{@{}rcl@{}} &&scoreRea(RMS_{s}\rightarrow RMS_{n}) \\ &&\quad= \sqrt[n-1]{\prod_{i={s}}^{n-1} wRea(RMS_{i}) \times\ \text{Pr}\ \left(RMS_{i+1} \mid RMS_{i} \right)} \end{array} $$

*ScorePageRank* and *scoreProt* are computed in the same way using *wPageRank* and *wProt*, respectively.

## Results and discussion

### From reaction to RMS networks

Among the 12,377 MetaCyc reactions, RMS of of height 1 (RMS-H1) and 2 (RMS-H2) have been computed for 9,001 reactions excluding transport reactions and reactions without proper compound structures as described in the Methods section. As shown in Table 1, RMS-H1 gathers on average about two times more reactions than RMS-H2. Indeed, RMS-H2 signatures give more precision about the chemical transformations than RMS-H1 as they encode additional information about the neighborhood of the reaction center that may be important for the chemical reactivity.

**Table 1:** Reaction molecular signature statistics

	Height 1	Height 2
Number of RMS	2477	4775
Number of reactions by RMS		
Minimum	1	1
Average	3.63	1.89
Maximum	312	144

**Fig. 2:**
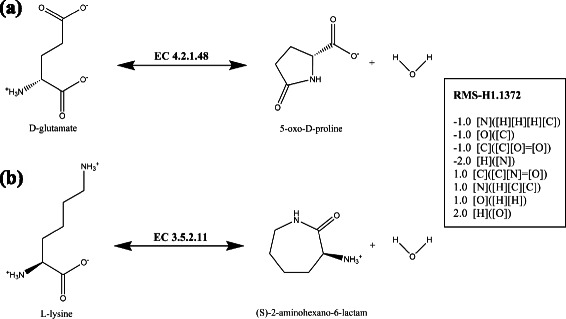
Example of reactions having a same RMS signature but classified in different EC classes. **a** D-glutamate cyclase reaction annotated with the EC 4.2.1.48. **b** L-lysine lactamase reaction annotated with EC 3.5.2.11. This both reactions make the same the chemical transformation represented by RMS-H1.1372, which encodes, in SMILES-like strings, the difference between the products and the substrates of atomic signatures of height 1

This fully automated chemical classification of reactions was compared with the Enzyme Commission (EC) classification which is a human expertise classification of enzymatic activities [31]. Even if efforts were made to automate the classification of new activities [17, 32, 33], the EC classification covers only half of all known enzymatic reactions. Among the 4,574 reactions linked both to an EC number and to a RMS, a simple similarity measure (Rand index) was computed between the third level sub-subclasses of EC numbers (179 classes) and the RMS-H1 (1,437 classes). We obtained a Rand index value of 97.68 % meaning, even if the RMS classification has a finer granularity, both classifications are thus similar (see Additional file 2 for detailed counts). Reactions classified in a same RMS tends to have the same third level EC class. Nevertheless, we found cases where the two classifications differs such as the example depicted in Fig. 2. From a chemical point of view, the D-glutamate cyclase and the L-lysine-lactamase reactions correspond to the formation or the hydrolysis of a lactam involving a primary amine and the carbon of the keto function of a carboxylic acid. These reactions are encoded by the same RMS but their EC classes differ: the D-glutamate cyclase is classified as a carbon-oxygen lyase (EC number 4.2.1.48), whereas the L-lysine-lactamase is a hydrolase acting on a carbon-nitrogen bond of a cyclic amide (EC number 3.5.2.11). These differences show that EC numbers are mainly focused on enzymatic activities and take in consideration the biological context to classify the reactions (*e.g.* the *in vivo* reaction directionality). These ambiguities, that are quite common between lyases and hydrolases or transferases, were also previously reported in other chemical classifications of reactions like MOLMAP [34].

Finally, an initial reaction network was established using metabolic pathway information from MetaCyc. It is made of 5,830 reaction nodes and 11,197 directed edges with an average node degree of 2.6. This graph was reduced in two RMS networks using RMS-H1 and H2 signatures. As summarized in Table 2, RMS networks are more compact than the reaction network: RMS-H1 and RMS-H2 networks contain a third and a half of nodes, respectively. By aggregating reactions in RMS nodes while preserving their initial connectivity, RMS graph structure should efficiently capture conserved paths of chemical reactions even for reactions not already associated to a metabolic pathway. Indeed, 2,278 reactions not included in the initial reaction network are linked to a chemical transformation context in the RMS networks since they are classified in the RMS networks with other reactions from known pathways.

**Table 2:** Statistics on reaction network and RMS networks

	Reaction	RMS-H1	RMS-H2
	network	network	network
Number of nodes	5830	1768	3365
Number of edges	11197	6107	8721
Average node degree	5.17	9.10	3.33
Average node out degree	2.60	4.36	2.99
Average node in degree	2.27	3.94	6.84
Node reduction rate	1	0.30	0.57

### Conserved RMS paths in metabolic pathways

**Fig. 3:**
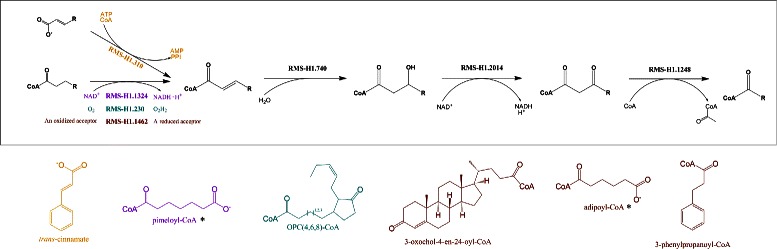
Conservation of *β*-oxidation module for non-fatty acid compounds. In addition to fatty acids, the *β*-oxidation module was found conserved for the transformation of 8 compounds represented in the figure. For the first step, we found 4 reaction variants encoded in different RMS of height 1: three RMS correspond to a dehydrogenation between the alpha and beta carbons but with different acceptors, another corresponds to a coenzyme A ligation. A color code indicates the corresponding substrates. Only molecules marked with an asterisk were also detected by Muto *et al.* (KEGG Reaction Module RM018)

**Fig. 4:**
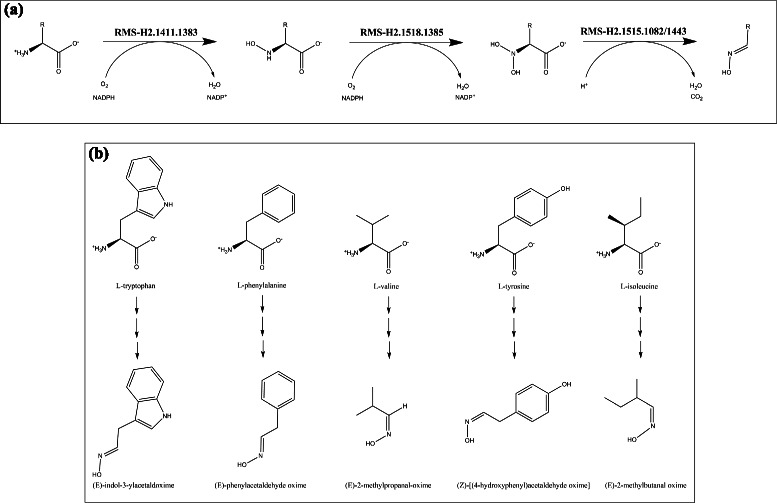
A conserved module for the biosynthesis of aldoximes from amino acids. **a** This module is made of three chemical transformations encoded by RMS-H2 signatures. It corresponds to the oxidative decarboxylation of an anmino acid to its aldoxime. **b** The module is conserved in different MetaCyc pathways for five distinct proteinogenic amino acids. Produced aldoximes are precursors of nitrogen-containing secondary metabolites in plants, like cyanogenic glycosides for seed germination and defense, or auxin phytohormones

An exploration of the RMS networks was conducted by an enumeration of all paths of length 1 (one edge, two RMS) to 4 (four edges, five RMS). To evaluate their conservation in the light of known metabolic pathways, a Pathway Conservation Index (PCI) was computed for each RMS path and corresponds to the number of distinct reaction paths present in MetaCyc pathways. The number of RMS paths with a PCI ≥2 is reported in Table 3 for each path length and for both signature heights. We found, for RMS-H1, between 117 and 600 conserved RMS paths depending of the path length and fewer paths (between 128 and 380) for RMS-H2 as they encode more precise signatures (see Additional file 3 for the complete list). They correspond to conserved chemical transformation modules, also named reaction modules in a previous study [15]. Indeed, Muto *et al.* obtained similar results but with a higher number of detected conserved paths (between 338 and 928 for the same path lengths). Although our results are not directly comparable to those of Muto *et al.* by the usage of different primary data sources (*i.e.* MetaCyc and KEGG, respectively), the RMS paths detected by our method can be directly considered as conserved modules whereas the paths obtained by Muto *et al.* need a manual examination to obtain conserved modules from them. In fact, they adopted a looser definition of chemical conservation without taking into account side compounds and using fingerprint similarities to group reactions without the constraint that the reactions perform the same chemical transformation. Only 34 reaction modules were finally confirmed by the authors [15]. Among the modules detected by our method, we found, for instance, that the *β*-oxidation pathway, that is well-known for fatty acid degradation, is also conserved for other molecule types (Fig. 3). This module, also detected by Muto *et al.* for a subset of compounds (two among eight), has four reaction variants in its first step. As another example, we detected a new three-step module for the biosynthesis of aldoximes from amino acids, which are notably precursors of several secondary metabolites produced by plants (Fig. 4). More generally, nearly half (48 %) of metabolic pathways contains at least one conserved module in the height 1 RMS network (see Table 4). Interestingly, pathways involved in the generation of precursor metabolites and energy (‘Energy’ type in Table 4) are the most conserved (78 % of them in RMS-H1 network). Besides, the proportion of conserved pathways involved in biosynthesis and degradation is also important and comparable for both types, 42 % and 47 % respectively.

**Table 3:** Number of conserved modules (*P* *C* *I*≥2)

Path length	RMS-H1 network	RMS-H2 network
1	600	380
2	365	214
3	212	141
4	117	128

**Table 4:** Number of pathways containing at least one conserved module (length 2, *P* *C* *I*≥2) classified by their type

Pathway type	RMS-H1 network	RMS-H2 network
Biosynthesis	263 (42 %)	154 (24 %)
Degradation	172 (47 %)	95 (25 %)
Detox	3 (27 %)	3 (23 %)
Energy	61 (78 %)	51 (65 %)
Other	19 (33 %)	10 (17 %)
All	518 (46 %)	313 (27 %)

### RMS path scoring and learning

**Fig. 5:**
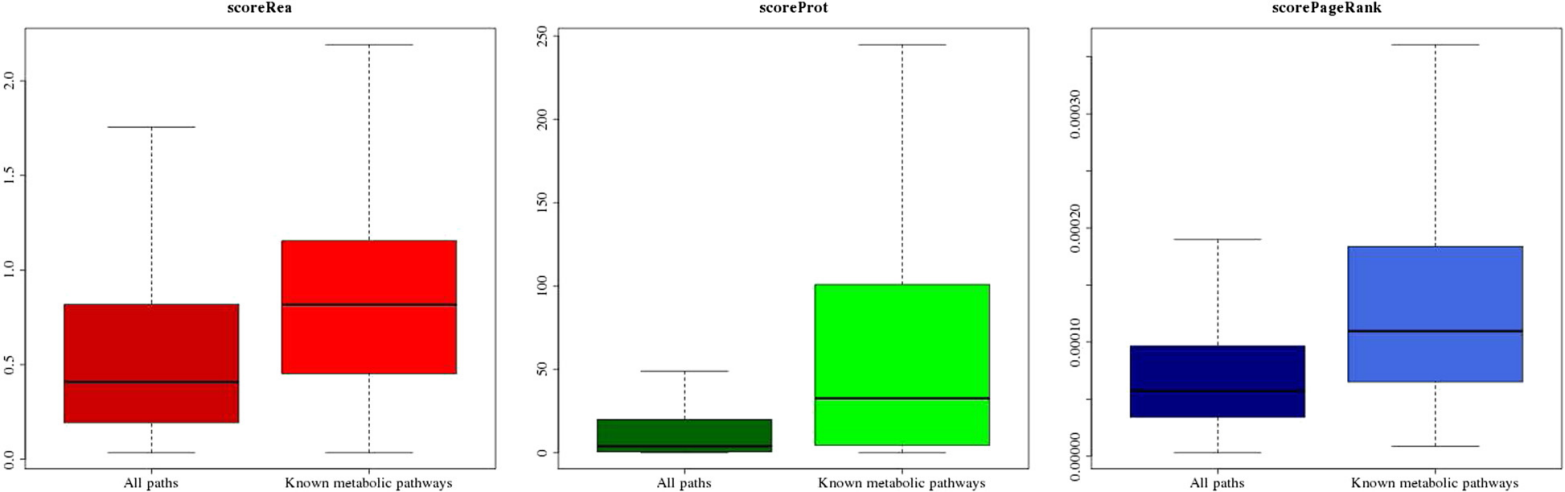
Boxplots of conservation scores for enumerated and known metabolic paths. For paths of length 2 (two edges and three nodes) in the RMS-H2 network, distributions of the three conservation scores (*i.e.* *scoreRea*, *scoreProt* and *scorePageRank*) are presented in all possible paths from the RMS network (identified as “All paths” in the figure) versus paths solely included in known metabolic pathways (“Known metabolic pathways”). The latter present significant higher scores (*p*-value <2*e* ^−16^ using Tukey’s HSD tests)

To go further, our method proposes an evaluation of chemical module conservation in the metabolism using three scores corresponding to different biological points of view. Indeed, *scoreRea* reflects the diversity of reactions performing the same chemical transformations on different substrates, *scoreProt* represents the conservation of enzymes performing these chemical transformations across the tree of life and *scorePageRank* shows the topological importance of the module in the network by highlighting chemical hubs. These scores were computed for all paths and analyzed more precisely for paths of length 2 in the RMS-H2 network (Table 5). It should be noticed that the *scoreProt* cannot be computed for about 20 % of paths as they contain at least one RMS without any known protein catalyzing the corresponding reactions, *i.e.* 30 % of the RMS-H2 correspond to orphan enzyme activities. As depicted in Fig. 5, paths from known metabolic pathways present statistically significant higher values for the three scores than in all possible paths computed from the RMS network (*p*-value <2*e* ^−16^ using Tukey’s HSD tests). Similar results were obtained for RMS-H1 network (see Additional file 4). These results confirm that the defined scores are useful to capture biologically relevant paths in the RMS network and should allow us to discover new metabolic modules. Furthermore, we found only a weak correlation between *scoreRea* and *scorePageRank* (Spearmans’ correlation coefficient of 0.66) and no correlation between other pairs of scores. Therefore, the proposed scores can be considered as rather independent and then used conjointly to explore the RMS network.

**Table 5:** Statistics on conservation scores for paths of length 2 in the RMS-H2 network

	ScoreRea	ScorePageRank	ScoreProt
All enumerated			
paths (*n* = 72173)			
Min score	0.04	3.32*e* ^−^6	4.39*e* ^−^4
Average score	0.61	7.69*e* ^−^5	25.17
Max score	17.58	1.20*e* ^−^3	3913.24
Paths in known			
pathways (*n* = 3001)			
Min score	0.04	8.63*e* ^−^6	7.81*e* ^−^4
Average score	1.07	1.55*e* ^−^4	118.57
Max score	17.58	1.20*e* ^−^3	3913.24

Next, these scores were analyzed in the light of MetaCyc pathway classification using five main types of biological processes: biosynthesis, degradation/utilization/assimilation, detoxification, generation of precursor metabolites and energy, and a last type, called “others”, that gathers other MetaCyc main pathway classes. By performing pairwise comparisons of pathway types (*i.e.* Kruskal-Wallis rank sum tests completed by *post-hoc* Tukey’s HSD tests, see Additional file 5), we found significant differences (*p*-values <0.05) among all pathway types for at least one of the three conservation scores. These results presume that pathway types could be predicted by machine learning using a combination of the three scores. Thus, pathway assignment rules were generated with the NNge algorithm [35, 36] implemented in Weka [37]. As the number of RMS paths per pathway type is very unbalanced (*e.g.* the “biosynthesis” class contains almost twice the number of paths than other types), classes were virtually balanced using resampling function of Weka. We successfully obtained rules that correctly classify RMS paths in pathway types with an accuracy greater than 89 % (see Additional file 6).

## Conclusions

We present here a novel metabolic network representation where nodes are chemical transformations depicted by reaction molecular signatures. This data model is particularly useful for finding conserved chemical transformation modules in metabolic pathways as they correspond to paths in the RMS network. An important number of modules was detected and could be integrated in metabolic databases, like KEGG [16] or MetaCyc [20], to help biologists looking for similar pathways. Furthermore, new metrics (*i.e.* *scoreRea*, *scoreProt* and *scorePageRank*) were introduced to evaluate module conservation according to different biological meanings. We show that known metabolic paths present higher score values than random ones and that the scores, used conjointly, may predict module pathway types. In terms of improvement of the graph reduction method, it may be of interest to dynamically adapt the precision of the reaction signatures when merging reaction nodes to take into account the local graph topology. This could be achieved taking inspiration from the method proposed by Xu *et al.* [38] in which the maximum entropy principle and the Markov chain model-reduction problem were applied. Finally, it should be highlighted that our method can be easily adapted to other types of reaction classifications based on chemical transformations.

Although its construction is based on an initial reaction network, the RMS network offers new insights into metabolism as it could capture relevant metabolic contexts even without precise definition of initial reaction sets or metabolite structures. Indeed, more than two thousand reactions lacking a metabolic pathway were integrated in the RMS network and now share common contexts with reactions from known pathways. Furthermore, considering that many orphan enzymes have network neighbours that are orphans themselves [2], computational tools [39, 40] have difficulties to find candidate genes for these missing enzymes by defining correct genomic contexts (*e.g.* chromosomal clusters, co-occurrence profiles) that include candidate proteins and known enzymes. As a perspective, one of the possible improvements of these methods could be the use of a RMS network instead of a reaction network as it may be easier to find proper genomic contexts using relaxed notions of metabolic context. This enhancement may also be applied in the discovery of gene clusters corresponding to new metabolic pathways.

## Additional files

Additional file 1
**Reaction molecular signature classification of reactions.** (XLSX 410 kb)

Additional file 2
**Comparison of RMS and enzyme commission reaction partitions.** (PDF 414 kb)

Additional file 3
**List of conserved chemical transformation modules.** They correspond to RMS paths present in known metabolic pathways with a PCI (Pathway Conservation Index) ≥2. (XLSX 76 kb)

Additional file 4
**Boxplots of conservation scores for enumerated and known metabolic paths of length 2 in the RMS-H1 network.** (PDF 306 kb)

Additional file 5
**Statistical analysis of path score distributions according to their pathway type.** Kruskal-Wallis and Tukey HSD statistical test results comparing scoreRea, scoreProt and scorePageRank distributions for paths in RMS-H1 and H2 networks belonging to at least one known metabolic pathway and depending on their pathway type. (PDF 317 kb)

Additional file 6
**Metabolic pathway type prediction rules generated by NNge algorithm.** NNge model and cross-validation results for pathway type prediction rules. (PDF 374 kb)
