# Supplementary material for: A new network representation of the metabolism to detect chemical transformation modules
Source: BMC Bioinformatics. 2015 Nov 14;16:385. doi: 10.1186/s12859-015-0809-4 (PMC4647279; doi:10.1186/s12859-015-0809-4)
Supplement: Additional file 2 — Comparison of RMS and enzyme commission reaction partitions. (PDF 414 kb) [file 12859_2015_809_MOESM2_ESM.pdf]

Additional file 2 – Comparison of Reaction Molecular Signature and Enzyme Commission reaction partition

- *a* is the number of reaction pairs that are in the same set in EC and in the same set in RMS = **73408**
- *b* is the number of reaction pairs that are in different sets in EC and in different sets in RMS = **10142098**
- *c* is the number of reaction pairs that are in the same set in EC and in different sets in RMS = **9946**
- *d* is the number of reaction pairs that are in different sets in EC and in the same set in RMS = **232984**

$$Rand\ Index = \frac{a+b}{a+b+c+d} = \frac{73408 + 10142098}{73408 + 10142098 + 9946 + 232984} = 0.976$$
