# Supplementary material for: A new network representation of the metabolism to detect chemical transformation modules
Source: BMC Bioinformatics. 2015 Nov 14;16:385. doi: 10.1186/s12859-015-0809-4 (PMC4647279; doi:10.1186/s12859-015-0809-4)

Additional file 4 – Boxplots of conservation scores for enumerated and known metabolic paths

For paths of length 2 (two edges and three nodes) in the RMS-H1 network, distributions of the three conservation scores (*i.e. scoreRea*, *scoreProt* and *scorePageRank*) are presented in all enumerated paths versus paths in known metabolic pathways.

The latter present significant higher scores (p-value <2e^-16 using Tukey's HSD tests)

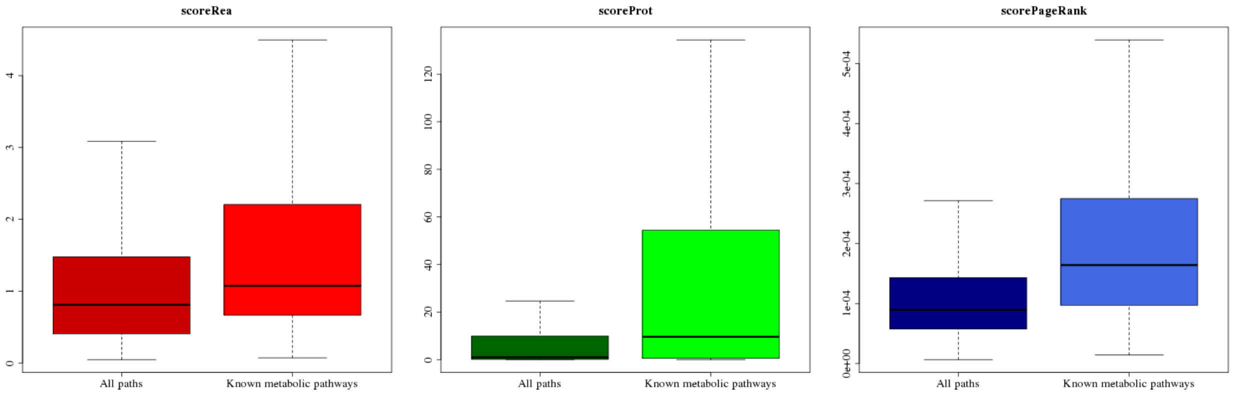

Supplement: Additional file 4 — Boxplots of conservation scores for enumerated and known metabolic paths of length 2 in the RMS-H1 network. (PDF 306 kb) [file 12859_2015_809_MOESM4_ESM.pdf]
