# Supplementary material for: A new network representation of the metabolism to detect chemical transformation modules
Source: BMC Bioinformatics. 2015 Nov 14;16:385. doi: 10.1186/s12859-015-0809-4 (PMC4647279; doi:10.1186/s12859-015-0809-4)
Supplement: Additional file 5 — Statistical analysis of path score distributions according to their pathway type. Kruskal-Wallis and Tukey HSD statistical test results comparing scoreRea, scoreProt and scorePageRank distributions for paths in RMS-H1 and H2 networks belonging to at least one known metabolic pathway and depending on their pathway type. (PDF 317 kb) [file 12859_2015_809_MOESM5_ESM.pdf]

Additional file 5 – Statistical analysis of conservation scores distributions according to the pathway type their paths are stemming from

Post-hoc analysis on metabolic pathway scores in order to determine if scores distributions are significantly different regarding the pathway type (biosynthesis, degradation, detoxification, energy or other). Are presented in following tables p-values from the Tukey HSD test for the three conservation scores (scoreRea, scorePageRank and scoreProt) for RMS paths from known metabolic pathways in height 2 RMS network.

Kruskal-Wallis rank sum tests for height 2 RMS network paths scores

H0: The distributions of path scores are identical regardless pathway type they are involved in

- **scoreRea** : Kruskal-Wallis chi-squared = 148.1694, df = 4, p-value < 2.2e-16
- **scoreProt** : Kruskal-Wallis chi-squared = 36.6593, df = 4, p-value = 2.117e-07
- **scorePageRank** : Kruskal-Wallis chi-squared = 66.2534, df = 4, p-value = 1.401e-13

Tukey HSD p-values for distribution comparison for height 2 RMS network paths of length 2.

Compared pathway types	scoreRea	scoreProt *(for all paths where scoreProt>0)*	scorePageRank
Degradation - Biosynthesis	0.05	0.03	0.000007
Detox – Biosynthesis	0.99	0.97	0.013
Energy – Biosynthesis	0	0.0001	0.55
Other – Biosynthesis	0.41	0.1	0.0005
Detox – Degradation	0.99	0.68	0.00005
Energy – Degradation	0.0000002	0.09	0.83
Other - Degradation	0.99	0.95	0.71
Energy – Detox	0.0067	0.14	0.0032
Other – Detox	0.98	0.53	0.000015
Other – Energy	0.0001	0.64	0.37
