# Supplementary material for: A new network representation of the metabolism to detect chemical transformation modules
Source: BMC Bioinformatics. 2015 Nov 14;16:385. doi: 10.1186/s12859-015-0809-4 (PMC4647279; doi:10.1186/s12859-015-0809-4)
Supplement: Additional file 6 — Metabolic pathway type prediction rules generated by NNge algorithm. NNge model and cross-validation results for pathway type prediction rules. (PDF 374 kb) [file 12859_2015_809_MOESM6_ESM.pdf]

## Additional file 6 – Metabolic pathway type prediction rules generated by NNge algorithm

```
Scheme:weka.classifiers.rules.NNge -G 20 -I 20
Attributes:   4
              scoreRea
              scoreProtTaxo
              scorePageRankTopoDiv
              t
Test mode:10-fold cross-validation
```

**=== Stratified cross-validation ===**
**=== Summary ===**

| | | |
|---|---|---|
| Correctly Classified Instances | 7822 | 94.7432 % |
| Incorrectly Classified Instances | 434 | 5.2568 % |
| Kappa statistic | 0.9076 | |
| Mean absolute error | 0.021 | |
| Root mean squared error | 0.145 | |
| Relative absolute error | 9.2047 % | |
| Root relative squared error | 42.9119 % | |
| Total Number of Instances | 8256 | |

=== Detailed Accuracy By Class ===

| | TP Rate | FP Rate | Precision | Recall | F-Measure | ROC Area | Class |
|---|---|---|---|---|---|---|---|
| | 0.922 | 0.028 | 0.927 | 0.922 | 0.925 | 0.947 | DEGRADATION |
| | 0.965 | 0.06 | 0.958 | 0.965 | 0.961 | 0.952 | BIOSYNTHESIS |
| | 0.929 | 0.003 | 0.947 | 0.929 | 0.938 | 0.963 | OTHER |
| | 0.869 | 0.001 | 0.926 | 0.869 | 0.897 | 0.934 | DETOX |
| | 0.935 | 0.004 | 0.939 | 0.935 | 0.937 | 0.966 | ENERGY |
| Weighted Avg. | 0.947 | 0.043 | 0.947 | 0.947 | 0.947 | 0.952 | |

=== Confusion Matrix ===

| a | b | c | d | e | <-- classified as |
|---|---|---|---|---|---|
| 2121 | 151 | 10 | 3 | 15 | a = DEGRADATION |
| 136 | 4672 | 16 | 6 | 13 | b = BIOSYNTHESIS |
| 13 | 22 | 469 | 0 | 1 | c = OTHER |
| 6 | 11 | 0 | 113 | 0 | d = DETOX |
| 11 | 20 | 0 | 0 | 447 | e = ENERGY |

**=== Classifier model (full training set) ===**

```
NNGE classifier

Rules generated :
        class ENERGY IF : 0.0944911182523068<=scoreRea<=0.11952286093343936 ^ 0.2380660236333224<=scoreProtTaxo<=2.467150522820092 ^ 3.9467331593969805E-
5<=scorePageRankTopoDiv<=8.222097127067186E-5  (19)
        class OTHER IF : 0.14824986333222023<=scoreRea<=0.23570226039551584 ^ 34.230955629673105<=scoreProtTaxo<=43.96658510801488 ^ 2.5624430194452117E-
5<=scorePageRankTopoDiv<=3.2215748110582064E-5  (25)
        class BIOSYNTHESIS IF : 1.3764944032233706<=scoreRea<=1.4142135623730951 ^ scoreProtTaxo=0.0 ^ 1.715924490479643E-4<=scorePageRankTopoDiv<=1.7442011676202887E-4
(9)
        class BIOSYNTHESIS IF : 0.5773502691896257<=scoreRea<=1.0 ^ scoreProtTaxo=0.0 ^ 2.635996114083793E-4<=scorePageRankTopoDiv<=2.6835762452210286E-4  (16)
        class BIOSYNTHESIS IF : 0.3333333333333333<=scoreRea<=0.3380617018914066 ^ scoreProtTaxo=0.0 ^ 1.7482405819301152E-4<=scorePageRankTopoDiv<=1.7796160572064972E-4
(8)
        class BIOSYNTHESIS IF : 0.6282808624375432<=scoreRea<=0.7071067811865476 ^ 137.05241439564665<=scoreProtTaxo<=187.6103739034471 ^ 8.941788011599709E-
5<=scorePageRankTopoDiv<=1.273031544422776E-4  (14)
```

```
        class BIOSYNTHESIS IF : 1.3844373104863457<=scoreRea<=1.4142135623730951 ^ 0.0<=scoreProtTaxo<=0.48131847175072956 ^ 1.2511393920411733E-4<=scorePageRankTopoDiv<=1.2782527724143538E-4  (12)
        class DEGRADATION IF : 0.5<=scoreRea<=1.0 ^ scoreProtTaxo=0.0 ^ 4.011826234288762E-5<=scorePageRankTopoDiv<=4.203593126592642E-5  (16)
        class DEGRADATION IF : scoreRea=0.46770717334674267 ^ scoreProtTaxo=0.9007059016979746 ^ scorePageRankTopoDiv=3.617856201725098E-5  (2)
        class BIOSYNTHESIS IF : 1.1547005383792515<=scoreRea<=2.5585578921327845 ^ 14.106547340156714<=scoreProtTaxo<=18.473453822095284 ^ 1.770564875558003E-4<=scorePageRankTopoDiv<=2.0789015753772057E-4  (13)
        class OTHER IF : scoreRea=0.7071067811865476 ^ scoreProtTaxo=0.0 ^ scorePageRankTopoDiv=1.9885864310046825E-4  (6)
        class BIOSYNTHESIS IF : 0.3535533905932738<=scoreRea<=0.408248290463863 ^ 0.12001422967741608<=scoreProtTaxo<=1.805290514655062 ^ 7.097145389737822E-5<=scorePageRankTopoDiv<=1.006856779682069E-4  (25)
        class ENERGY IF : scoreRea=0.24743582965269673 ^ scoreProtTaxo=45.5775940290842 ^ scorePageRankTopoDiv=6.303181454151838E-5  (3)
        class BIOSYNTHESIS IF : scoreRea=1.4142135623730951 ^ scoreProtTaxo=0.0 ^ scorePageRankTopoDiv=3.4486469678956296E-4  (3)
        class BIOSYNTHESIS IF : 2.3145502494313788<=scoreRea<=4.636809247747852 ^ 0.13557591986987977<=scoreProtTaxo<=40.39566525235376 ^ 6.279108186413624E-5<=scorePageRankTopoDiv<=1.1946954132219554E-4  (26)
        class BIOSYNTHESIS IF : 1.7320508075688772<=scoreRea<=3.055050463303893 ^ 98.40420334876411<=scoreProtTaxo<=378.82319006045105 ^ 1.2993501217585093E-4<=scorePageRankTopoDiv<=1.3143712413214126E-4  (6)
        class DEGRADATION IF : 0.7071067811865476<=scoreRea<=0.8320502943378437 ^ 20.577608238503228<=scoreProtTaxo<=70.40532050487963 ^ 1.4711128835871555E-4<=scorePageRankTopoDiv<=1.5582750065655076E-4  (6)
        class ENERGY IF : 0.14680505487867587<=scoreRea<=0.1749635530559413 ^ 1.5153219406847809<=scoreProtTaxo<=59.68013546214413 ^ 4.522582100052408E-5<=scorePageRankTopoDiv<=6.316112041496758E-5  (12)
        class BIOSYNTHESIS IF : 1.5811388300841898<=scoreRea<=3.3806170189140663 ^ 89.64616712273855<=scoreProtTaxo<=93.87272426424039 ^ 4.616620362534685E-4<=scorePageRankTopoDiv<=6.13379441752335E-4  (8)
        class BIOSYNTHESIS IF : 0.1714399631667259<=scoreRea<=0.75 ^ 0.35675283566636734<=scoreProtTaxo<=0.5896504247749174 ^ 2.5285969726396953E-5<=scorePageRankTopoDiv<=4.035096476002114E-5  (30)
        class BIOSYNTHESIS IF : 1.4675987714106857<=scoreRea<=1.5275252316519465 ^ scoreProtTaxo=0.0 ^ 1.4824279368028657E-4<=scorePageRankTopoDiv<=1.873432940507961E-4 (7)
        class DEGRADATION IF : 0.4330127018922193<=scoreRea<=0.6064172948423149 ^ 0.5629775241084929<=scoreProtTaxo<=36.00546680035225 ^ 4.043811427310258E-5<=scorePageRankTopoDiv<=4.107288757033667E-5  (17)
        class DEGRADATION IF : scoreRea=1.3363062095621219 ^ scoreProtTaxo=59.8217421667204 ^ scorePageRankTopoDiv=6.927263515039377E-5  (4)
        class BIOSYNTHESIS IF : scoreRea=1.0 ^ scoreProtTaxo=0.0 ^ 2.4385616569174256E-4<=scorePageRankTopoDiv<=2.4446999316006323E-4  (12)
        class BIOSYNTHESIS IF : 1.3693063937629153<=scoreRea<=2.4776781245530843 ^ 0.050541374706292774<=scoreProtTaxo<=40.716794145116985 ^ 4.4582055374839147E-4<=scorePageRankTopoDiv<=5.910409514342583E-4  (23)
        class BIOSYNTHESIS IF : 0.28867513459481287<=scoreRea<=0.6695340634119862 ^ 0.0<=scoreProtTaxo<=0.09750442980201487 ^ 2.3078302774459267E-5<=scorePageRankTopoDiv<=2.654390416091464E-5  (19)
        class DEGRADATION IF : 1.0<=scoreRea<=1.0933445471810679 ^ scoreProtTaxo=0.0 ^ 1.5119971642290792E-4<=scorePageRankTopoDiv<=1.5165145812969067E-4  (11)
        class DEGRADATION IF : 0.3535533905932738<=scoreRea<=0.408248290463863 ^ scoreProtTaxo=0.0 ^ 1.2760655041187276E-4<=scorePageRankTopoDiv<=1.8234241192197802E-4 (20)
        class BIOSYNTHESIS IF : 0.42491829279939874<=scoreRea<=0.4629100498862757 ^ 0.0019539003218056023<=scoreProtTaxo<=10.579425554589232 ^ 6.0762810298399056E-5<=scorePageRankTopoDiv<=6.0772374429749914E-5  (11)
        class BIOSYNTHESIS IF : 1.118033988749895<=scoreRea<=1.5491933384829668 ^ 28.3876733812448<=scoreProtTaxo<=171.2522187159527 ^ 1.214796345552975E-4<=scorePageRankTopoDiv<=1.339312706332986E-4  (38)
        class BIOSYNTHESIS IF : 0.16666666666666666<=scoreRea<=0.5477225575051662 ^ 211.6065492558517<=scoreProtTaxo<=354.05109611467765 ^ 2.4353028660813146E-5<=scorePageRankTopoDiv<=1.12430516234085E-4  (38)
        class DEGRADATION IF : 0.408248290463863<=scoreRea<=0.5443310539518174 ^ 0.05342416342328977<=scoreProtTaxo<=9.574954409747393 ^ 6.276174469714381E-5<=scorePageRankTopoDiv<=6.541810444814617E-5  (15)
        class DEGRADATION IF : scoreRea=0.5773502691896257 ^ scoreProtTaxo=150.93034502106704 ^ scorePageRankTopoDiv=1.3168334991139308E-4  (7)
        class DEGRADATION IF : 0.49507377148833714<=scoreRea<=0.6370220572706061 ^ 3.116903115717679<=scoreProtTaxo<=10.859160785877924 ^ 3.192381751198706E-5<=scorePageRankTopoDiv<=3.283073109307133E-5  (4)
        class DEGRADATION IF : 0.9848476085314292<=scoreRea<=1.311651671567906 ^ 0.0015315028922544994<=scoreProtTaxo<=0.012958469829493875 ^ 1.6285332302952283E-4<=scorePageRankTopoDiv<=1.6807726764800455E-4  (10)
        class DEGRADATION IF : 1.5146344928922038<=scoreRea<=1.5387160422974504 ^ 4.37231962355855<=scoreProtTaxo<=45.81247528729291 ^ 1.0060730209984355E-4<=scorePageRankTopoDiv<=1.0542570447093832E-4  (2)
        class DEGRADATION IF : scoreRea=1.4950900031928038 ^ scoreProtTaxo=23.643450906127864 ^ scorePageRankTopoDiv=1.0170788148269443E-4  (4)
        class DEGRADATION IF : 1.4038890593022617<=scoreRea<=1.5275252316519465 ^ 0.0<=scoreProtTaxo<=0.22494079551498097 ^ 1.0295047718367274E-4<=scorePageRankTopoDiv<=1.0359361723184283E-4  (11)
        class DEGRADATION IF : 1.5191090506255<=scoreRea<=1.632993161855452 ^ scoreProtTaxo=0.0 ^ 8.767676776912526E-5<=scorePageRankTopoDiv<=9.866342288397187E-5  (6)
        class DEGRADATION IF : 1.1473127431577863<=scoreRea<=1.695582495781317 ^ 0.4173866279031499<=scoreProtTaxo<=0.6904483523297835 ^ 8.283363561372424E-5<=scorePageRankTopoDiv<=1.1294034404114938E-4  (28)
        class BIOSYNTHESIS IF : scoreRea=1.495090003192804 ^ scoreProtTaxo=23.643450906127864 ^ scorePageRankTopoDiv=1.0170788148269442E-4  (2)
        class BIOSYNTHESIS IF : 0.1767766952966369<=scoreRea<=0.18257418583505536 ^ 1.4521302510479395<=scoreProtTaxo<=4.3847354266734015 ^ 1.872670084424061E-5<=scorePageRankTopoDiv<=2.0210433645597785E-5  (14)
        class DEGRADATION IF : 1.6519994452731097<=scoreRea<=1.7320508075688772 ^ 0.0<=scoreProtTaxo<=0.11859382190794628 ^ 1.659536769761936E-4<=scorePageRankTopoDiv<=2.1296498231818996E-4  (10)
        class DEGRADATION IF : 1.0<=scoreRea<=1.1547005383792515 ^ 0.0<=scoreProtTaxo<=0.5811794521613008 ^ 7.132515955266688E-5<=scorePageRankTopoDiv<=7.875167426911117E-5  (31)
        class DEGRADATION IF : 0.08006407690254357<=scoreRea<=0.08425254637422692 ^ 0.7466289594192045<=scoreProtTaxo<=10.949924907214125 ^ 2.839311395403713E-5<=scorePageRankTopoDiv<=3.3883828455200255E-5  (8)
        class BIOSYNTHESIS IF : scoreRea=0.25 ^ scoreProtTaxo=0.0 ^ scorePageRankTopoDiv=3.1827594371485E-5  (2)
```

class BIOSYNTHESIS IF : 0.30151134457776363<=scoreRea<=0.35805743701971643 ^ 33.13265144118489<=scoreProtTaxo<=165.40664584751835 ^ 2.1781192991062262E-5<=scorePageRankTopoDiv<=3.331904960288265E-5 (29)
class DEGRADATION IF : scoreRea=2.0 ^ scoreProtTaxo=52.67513222304043 ^ scorePageRankTopoDiv=2.4625803787683525E-4 (6)
class DEGRADATION IF : scoreRea=0.5 ^ scoreProtTaxo=51.0656694346673 ^ scorePageRankTopoDiv=6.415037616460833E-5 (7)
class BIOSYNTHESIS IF : scoreRea=0.7071067811865476 ^ scoreProtTaxo=0.0 ^ 1.7047280223499706E-4<=scorePageRankTopoDiv<=1.7681757651857256E-4 (23)
class BIOSYNTHESIS IF : 0.5<=scoreRea<=0.5345224838248488 ^ 92.58392887825794<=scoreProtTaxo<=645.2183120206236 ^ 1.8665619280014382E-4<=scorePageRankTopoDiv<=3.987942542101376E-4 (34)
class BIOSYNTHESIS IF : 0.816496580927726<=scoreRea<=1.6770509831248424 ^ 0.02414552317287656<=scoreProtTaxo<=60.881932950570835 ^ 2.8126292026083763E-5<=scorePageRankTopoDiv<=4.606334372579576E-5 (39)
class BIOSYNTHESIS IF : 0.8944271909999159<=scoreRea<=1.0053333711589283 ^ 17.297810329416045<=scoreProtTaxo<=71.7572486719615 ^ 6.17572370468687E-5<=scorePageRankTopoDiv<=6.430819501851288E-5 (26)
class BIOSYNTHESIS IF : 3.872983346207417<=scoreRea<=5.366563145999495 ^ 0.0<=scoreProtTaxo<=0.15260553574759061 ^ 1.400236268308033E-4<=scorePageRankTopoDiv<=4.1749250632250665E-4 (39)
class DEGRADATION IF : scoreRea=1.0 ^ scoreProtTaxo=0.0 ^ 1.915262928751791E-4<=scorePageRankTopoDiv<=1.9197527419422426E-4 (6)
class BIOSYNTHESIS IF : 1.0540925533894596<=scoreRea<=1.2909944487358058 ^ 27.91959473984153<=scoreProtTaxo<=59.62840121189813 ^ 1.7853466287135828E-4<=scorePageRankTopoDiv<=2.449337716498709E-4 (21)
class BIOSYNTHESIS IF : 0.7637626158259734<=scoreRea<=0.8944271909999159 ^ 222.78010111168732<=scoreProtTaxo<=260.81456976412017 ^ 7.669479555608071E-5<=scorePageRankTopoDiv<=1.253740888154235E-4 (14)
class DEGRADATION IF : scoreRea=0.4472135954999579 ^ scoreProtTaxo=0.0 ^ 1.2853202642214428E-4<=scorePageRankTopoDiv<=1.297143538338404E-4 (6)
class BIOSYNTHESIS IF : 1.741143000264028<=scoreRea<=1.9578900207451218 ^ 0.043526169552462184<=scoreProtTaxo<=0.4496737139612123 ^ 7.047799086950667E-5<=scorePageRankTopoDiv<=1.3754176330353984E-4 (9)
class BIOSYNTHESIS IF : 2.0<=scoreRea<=2.23606797749979 ^ scoreProtTaxo=0.0 ^ 1.3107866933148774E-4<=scorePageRankTopoDiv<=1.5035776480829834E-4 (16)
class BIOSYNTHESIS IF : 0.4330127018922193<=scoreRea<=0.7071067811865476 ^ 37.96986447132463<=scoreProtTaxo<=47.476763977188085 ^ 6.0575541963087874E-5<=scorePageRankTopoDiv<=8.184055624663284E-5 (38)
class BIOSYNTHESIS IF : 0.4216370213557839<=scoreRea<=0.6666666666666666 ^ 0.0<=scoreProtTaxo<=29.109935803778434 ^ 2.9791555817155357E-4<=scorePageRankTopoDiv<=4.7186282623621316E-4 (34)
class DEGRADATION IF : 0.12964074471043288<=scoreRea<=0.14625448482542613 ^ 11.675943716030119<=scoreProtTaxo<=12.568556538811487 ^ 5.304913067771752E-5<=scorePageRankTopoDiv<=5.665850725133843E-5 (3)
class DEGRADATION IF : scoreRea=0.10846522890932808 ^ scoreProtTaxo=0.0 ^ scorePageRankTopoDiv=5.484507680933927E-5 (6)
class DEGRADATION IF : 0.13693063937629155<=scoreRea<=0.16484511834894675 ^ 0.1642099863440641<=scoreProtTaxo<=1.4106555334621784 ^ 4.765343982056646E-5<=scorePageRankTopoDiv<=5.694991249826218E-5 (9)
class DEGRADATION IF : 1.6064386578049976<=scoreRea<=1.6449566416599486 ^ scoreProtTaxo=0.0 ^ 1.1726683964274192E-4<=scorePageRankTopoDiv<=1.449097451000517E-4 (5)
class BIOSYNTHESIS IF : 0.29277002188455997<=scoreRea<=0.5014598571212789 ^ 0.3353271363778662<=scoreProtTaxo<=24.040204420303578 ^ 5.49874466379079E-5<=scorePageRankTopoDiv<=5.58575845653719E-5 (8)
class DEGRADATION IF : scoreRea=1.4142135623730951 ^ scoreProtTaxo=0.0 ^ 1.744724650945507E-4<=scorePageRankTopoDiv<=2.034274877002086E-4 (15)
class BIOSYNTHESIS IF : 1.1881770515720091<=scoreRea<=1.5029382986043587 ^ 0.0<=scoreProtTaxo<=22.635275882667845 ^ 5.641772204858564E-5<=scorePageRankTopoDiv<=7.202261364213803E-5 (30)
class DEGRADATION IF : scoreRea=1.0 ^ scoreProtTaxo=0.0 ^ scorePageRankTopoDiv=9.184702952142395E-5 (2)
class DEGRADATION IF : scoreRea=0.9274777915203366 ^ scoreProtTaxo=0.5535860737834317 ^ scorePageRankTopoDiv=1.037932024148532E-4 (3)
class DEGRADATION IF : 0.50709255283711<=scoreRea<=1.1631599960755994 ^ 89.65243916336601<=scoreProtTaxo<=100.84577451615787 ^ 7.732571433805503E-5<=scorePageRankTopoDiv<=8.535041606710396E-5 (10)
class DEGRADATION IF : 1.224744871391589<=scoreRea<=1.3093073414159542 ^ scoreProtTaxo=0.0 ^ 7.82482349181678E-5<=scorePageRankTopoDiv<=9.00962259154721E-5 (5)
class DEGRADATION IF : 1.1547005383792515<=scoreRea<=1.2524485821702989 ^ 11.082570599674515<=scoreProtTaxo<=33.413640851598345 ^ 7.335639963672055E-5<=scorePageRankTopoDiv<=8.351353906970459E-5 (7)
class DEGRADATION IF : 0.8041247051007595<=scoreRea<=0.816496580927726 ^ 0.0<=scoreProtTaxo<=0.4896220574897665 ^ 9.817141430648813E-5<=scorePageRankTopoDiv<=1.0140742186772612E-4 (5)
class DEGRADATION IF : 0.7745966692414834<=scoreRea<=0.7905694150420949 ^ 14.979713836562523<=scoreProtTaxo<=62.92772912755101 ^ 9.188674496119563E-5<=scorePageRankTopoDiv<=9.344114513260257E-5 (9)
class DEGRADATION IF : 0.9354143466934853<=scoreRea<=1.0 ^ 28.41909081319826<=scoreProtTaxo<=81.7234468469862 ^ 7.509686129369359E-5<=scorePageRankTopoDiv<=8.22600297747681E-5 (10)
class DEGRADATION IF : scoreRea=0.8469010445797931 ^ scoreProtTaxo=29.848706892410423 ^ scorePageRankTopoDiv=9.674817445529926E-5 (2)
class DEGRADATION IF : scoreRea=1.0 ^ scoreProtTaxo=0.0 ^ 8.076306394889498E-5<=scorePageRankTopoDiv<=8.197307718578433E-5 (8)
class DEGRADATION IF : 0.7494854201795578<=scoreRea<=0.985184366143778 ^ 10.922282224695822<=scoreProtTaxo<=17.77683221349325 ^ 9.980433002573033E-5<=scorePageRankTopoDiv<=1.0634169150311942E-4 (10)
class DEGRADATION IF : 1.018350154434631<=scoreRea<=1.224744871391589 ^ 22.39494298336476<=scoreProtTaxo<=30.169323270783604 ^ 9.814808299652264E-5<=scorePageRankTopoDiv<=9.975494599478356E-5 (10)
class DEGRADATION IF : 1.0954451150103324<=scoreRea<=1.1547005383792515 ^ scoreProtTaxo=0.0 ^ 9.654374763933151E-5<=scorePageRankTopoDiv<=1.0165350230008272E-4 (5)
class DEGRADATION IF : scoreRea=0.9537823244295424 ^ scoreProtTaxo=0.0362368127852091 ^ scorePageRankTopoDiv=9.728342671754269E-5 (4)
class DEGRADATION IF : scoreRea=0.9428090415820634 ^ scoreProtTaxo=40.261490960005546 ^ scorePageRankTopoDiv=9.140981407470723E-5 (3)
class DEGRADATION IF : scoreRea=0.9274777915203366 ^ scoreProtTaxo=0.34489461142032407 ^ scorePageRankTopoDiv=8.803860903748013E-5 (6)
class DEGRADATION IF : scoreRea=1.0 ^ scoreProtTaxo=16.564961694015768 ^ scorePageRankTopoDiv=9.38994836757291E-5 (2)
class DEGRADATION IF : scoreRea=1.0 ^ scoreProtTaxo=0.0 ^ scorePageRankTopoDiv=8.952154062194338E-5 (8)
class DEGRADATION IF : 0.9746794344808964<=scoreRea<=1.0 ^ 2.460269816937521<=scoreProtTaxo<=4.622017759238929 ^ 7.201957047401327E-5<=scorePageRankTopoDiv<=1.0315296708591542E-4 (13)
class BIOSYNTHESIS IF : scoreRea=1.0 ^ scoreProtTaxo=0.0 ^ scorePageRankTopoDiv=9.069895169067138E-5 (3)

```
class DEGRADATION IF : 0.45374260648651504<=scoreRea<=0.4629100498862757 ^ 0.0<=scoreProtTaxo<=1.8771129021538737 ^ 9.224164089012893E-5<=scorePageRankTopoDiv<=1.0071405108661644E-4  (6)
class BIOSYNTHESIS IF : 0.5773502691896257<=scoreRea<=0.6708203932499369 ^ 274.70343271754246<=scoreProtTaxo<=766.5790693441894 ^ 4.5131690253895185E-5<=scorePageRankTopoDiv<=1.2501321987107045E-4  (26)
class BIOSYNTHESIS IF : 0.3779644730092272<=scoreRea<=0.5773502691896257 ^ 6.102381276116212<=scoreProtTaxo<=11.022017678245241 ^ 1.8888026238506796E-4<=scorePageRankTopoDiv<=2.0115792013336494E-4  (19)
class ENERGY IF : scoreRea=0.7150969419341943 ^ scoreProtTaxo=1.6971631401665692 ^ scorePageRankTopoDiv=1.0511897867639374E-4  (6)
class BIOSYNTHESIS IF : scoreRea=0.7071067811865476 ^ 52.65882180233855<=scoreProtTaxo<=797.4352638302629 ^ 1.8282788138177227E-4<=scorePageRankTopoDiv<=2.7540019598240703E-4  (35)
class DEGRADATION IF : 0.2581988897471611<=scoreRea<=2.6457513110645907 ^ 0.0<=scoreProtTaxo<=38.05578532590208 ^ 5.2686476747723835E-5<=scorePageRankTopoDiv<=5.302790721846163E-5  (18)
class DEGRADATION IF : scoreRea=1.0 ^ scoreProtTaxo=122.91212562233233 ^ scorePageRankTopoDiv=1.6215651469509557E-4  (7)
class ENERGY IF : 0.2637521893583148<=scoreRea<=0.3730019232961255 ^ 60.86198732982632<=scoreProtTaxo<=86.07184791481986 ^ 1.6848744964407823E-4<=scorePageRankTopoDiv<=2.3827723637630932E-4  (23)
class BIOSYNTHESIS IF : 1.0954451150103324<=scoreRea<=1.3228756555322954 ^ 82.12786352410112<=scoreProtTaxo<=119.36925641394684 ^ 1.0627502598211805E-4<=scorePageRankTopoDiv<=1.1655246691256627E-4  (9)
class BIOSYNTHESIS IF : 1.9148542155126762<=scoreRea<=2.1908902300206647 ^ scoreProtTaxo=0.0 ^ 1.64678663392129E-4<=scorePageRankTopoDiv<=2.181666849824038E-4  (29)
class BIOSYNTHESIS IF : 0.3939192985791677<=scoreRea<=0.7833494518006402 ^ 169.97802057365087<=scoreProtTaxo<=183.56764420773487 ^ 1.253685083966662E-5<=scorePageRankTopoDiv<=7.240151514526351E-5  (17)
class BIOSYNTHESIS IF : 0.7071067811865476<=scoreRea<=0.8498365855987975 ^ 0.0<=scoreProtTaxo<=0.2705728571551526 ^ 1.7988095066634002E-4<=scorePageRankTopoDiv<=1.9845237065041013E-4  (23)
class BIOSYNTHESIS IF : 0.7337993857053428<=scoreRea<=0.7947194142390263 ^ 45.944098229304075<=scoreProtTaxo<=81.91194155996973 ^ 7.613039874230699E-5<=scorePageRankTopoDiv<=7.666851418879933E-5  (16)
class BIOSYNTHESIS IF : 0.2581988897471611<=scoreRea<=0.5477225575051662 ^ 5.532750116334787<=scoreProtTaxo<=7.5348917327195295 ^ 4.1810101055858513E-5<=scorePageRankTopoDiv<=4.352867374835336E-5  (19)
class BIOSYNTHESIS IF : scoreRea=0.7071067811865476 ^ scoreProtTaxo=0.0 ^ 6.722085282994288E-5<=scorePageRankTopoDiv<=7.902934973945932E-5  (20)
class BIOSYNTHESIS IF : 0.6577935144802719<=scoreRea<=0.9428090415820634 ^ 22.113552769424796<=scoreProtTaxo<=135.82372101723521 ^ 9.796435133998824E-5<=scorePageRankTopoDiv<=1.0691434479612118E-4  (21)
class DEGRADATION IF : 0.6340037731068526<=scoreRea<=0.655825835783953 ^ 1.1516798782906763<=scoreProtTaxo<=1.4843251210366566 ^ 7.791134999632446E-5<=scorePageRankTopoDiv<=7.928770882931432E-5  (7)
class BIOSYNTHESIS IF : 0.30151134457776363<=scoreRea<=0.7905694150420949 ^ 17.01863620075732<=scoreProtTaxo<=19.559777958158104 ^ 1.319538661068268E-4<=scorePageRankTopoDiv<=1.9256485630141714E-4  (22)
class ENERGY IF : 0.18650096164806276<=scoreRea<=0.25 ^ 43.03592395740993<=scoreProtTaxo<=159.8937216743261 ^ 1.065214877529157E-4<=scorePageRankTopoDiv<=1.2057775407382344E-4  (14)
class BIOSYNTHESIS IF : 0.2041241452319315<=scoreRea<=0.8100925873009825 ^ 0.847877770446752<=scoreProtTaxo<=1.074881257772181 ^ 4.479895441755911E-5<=scorePageRankTopoDiv<=5.224748600927068E-5  (43)
class BIOSYNTHESIS IF : 1.4142135623730951<=scoreRea<=1.7320508075688772 ^ 0.0<=scoreProtTaxo<=0.0017025599442490314 ^ 2.2512882518055442E-4<=scorePageRankTopoDiv<=2.318761271239482E-4  (9)
class BIOSYNTHESIS IF : 1.0<=scoreRea<=1.741143000264028 ^ 0.9547413240085996<=scoreProtTaxo<=1.087186042233232 ^ 7.339287726861064E-5<=scorePageRankTopoDiv<=2.2506623584351968E-4  (14)
class BIOSYNTHESIS IF : 1.3764944032233706<=scoreRea<=1.8257418583505536 ^ 0.21419754504487418<=scoreProtTaxo<=0.9059002542530397 ^ 1.4570108837029384E-4<=scorePageRankTopoDiv<=2.873744946297966E-4  (13)
class BIOSYNTHESIS IF : scoreRea=1.0 ^ scoreProtTaxo=0.0 ^ 1.9671667363256823E-4<=scorePageRankTopoDiv<=1.9910561982231135E-4  (15)
class DEGRADATION IF : scoreRea=1.0 ^ 53.30721343159817<=scoreProtTaxo<=65.92162734927443 ^ 2.0561721671014314E-4<=scorePageRankTopoDiv<=2.1409527794787715E-4  (10)
class BIOSYNTHESIS IF : 0.5773502691896257<=scoreRea<=0.7071067811865476 ^ scoreProtTaxo=0.0 ^ 2.0636566208728405E-4<=scorePageRankTopoDiv<=2.0648542319897373E-4  (8)
class DEGRADATION IF : scoreRea=1.955384722187607 ^ scoreProtTaxo=74.18271953470759 ^ scorePageRankTopoDiv=8.258174752930541E-5  (3)
class ENERGY IF : 0.07715167498104596<=scoreRea<=0.12838814775327387 ^ 2.769418330851198<=scoreProtTaxo<=58.32783116309947 ^ 5.124932507561116E-5<=scorePageRankTopoDiv<=5.800682326550666E-5  (10)
class BIOSYNTHESIS IF : 0.22360679774997896<=scoreRea<=0.2672612419124244 ^ 1.141503527382941<=scoreProtTaxo<=13.075793923503037 ^ 2.623148740185707E-5<=scorePageRankTopoDiv<=2.9645682753051047E-5  (34)
class DEGRADATION IF : scoreRea=0.2587745847533828 ^ scoreProtTaxo=5.139256758115308 ^ scorePageRankTopoDiv=2.3863079676344778E-5  (4)
class DEGRADATION IF : 0.9428090415820634<=scoreRea<=1.0 ^ scoreProtTaxo=0.0 ^ 1.4502820216803015E-4<=scorePageRankTopoDiv<=1.4627385295981182E-4  (16)
class BIOSYNTHESIS IF : 2.860387767736777<=scoreRea<=3.4070284908193678 ^ 0.073153224734412<=scoreProtTaxo<=12.354434163067936 ^ 1.2498272102971447E-4<=scorePageRankTopoDiv<=2.4284228051064344E-4  (36)
class DEGRADATION IF : 0.2123976976214366<=scoreRea<=0.25 ^ scoreProtTaxo=0.0 ^ 3.586505967582538E-5<=scorePageRankTopoDiv<=4.13572229377226E-5  (3)
class OTHER IF : 0.16666666666666666<=scoreRea<=0.32163376045133846 ^ 50.57748410849425<=scoreProtTaxo<=60.41469432517077 ^ 9.017824067309039E-6<=scorePageRankTopoDiv<=1.9368859864889623E-5  (31)
class DEGRADATION IF : 0.4330127018922193<=scoreRea<=0.445545919354113 ^ 0.3873573198705962<=scoreProtTaxo<=6.126887661574918 ^ 8.022070904715247E-5<=scorePageRankTopoDiv<=8.382281480674639E-5  (5)
class DEGRADATION IF : 0.5773502691896257<=scoreRea<=0.5976143046671968 ^ scoreProtTaxo=0.0 ^ 9.293451231433748E-5<=scorePageRankTopoDiv<=9.543701626424988E-5  (5)
class DEGRADATION IF : scoreRea=0.408248290463863 ^ scoreProtTaxo=0.0 ^ scorePageRankTopoDiv=8.759460449880492E-5  (1)
class DEGRADATION IF : 0.49065338146265813<=scoreRea<=0.5 ^ scoreProtTaxo=0.0 ^ 8.136860247612824E-5<=scorePageRankTopoDiv<=8.749759780663291E-5  (8)
class DEGRADATION IF : 0.4264014327112209<=scoreRea<=0.4330127018922193 ^ 0.0<=scoreProtTaxo<=5.833138966641785 ^ 8.865063231330154E-5<=scorePageRankTopoDiv<=9.041648195074965E-5  (5)
```

class BIOSYNTHESIS IF : scoreRea=0.408248290463863 ^ scoreProtTaxo=0.0 ^ scorePageRankTopoDiv=1.0024486181235397E-4  (3)
class BIOSYNTHESIS IF : scoreRea=0.5 ^ scoreProtTaxo=0.0 ^ 9.144319250957409E-5<=scorePageRankTopoDiv<=1.0459222146356342E-4  (17)
class BIOSYNTHESIS IF : 0.48507125007266594<=scoreRea<=0.7559289460184544 ^ 14.876157414391628<=scoreProtTaxo<=63.77848346900098 ^ 1.1719585732214434E-4<=scorePageRankTopoDiv<=1.2423485591910442E-4  (30)
class BIOSYNTHESIS IF : 1.35400640077266<=scoreRea<=3.289913283319897 ^ 602.4307334503723<=scoreProtTaxo<=970.2809711123874 ^ 3.2452727807493777E-4<=scorePageRankTopoDiv<=3.6817602382859356E-4  (8)
class BIOSYNTHESIS IF : 0.8637684928578397<=scoreRea<=1.2433397443204184 ^ 0.7182944373783682<=scoreProtTaxo<=0.9403608156667465 ^ 7.247323472250873E-5<=scorePageRankTopoDiv<=9.936154209859977E-5  (14)
class BIOSYNTHESIS IF : 0.6324555320336759<=scoreRea<=1.224744871391589 ^ 122.60072594474337<=scoreProtTaxo<=200.61081401361804 ^ 2.9188234495044796E-4<=scorePageRankTopoDiv<=3.3305101431035033E-4  (17)
class BIOSYNTHESIS IF : 0.18257418583505536<=scoreRea<=0.2041241452319315 ^ 14.689588149434279<=scoreProtTaxo<=29.97574019102781 ^ 4.0274853010570464E-5<=scorePageRankTopoDiv<=4.312415052681157E-5  (9)
class BIOSYNTHESIS IF : 1.23418791690791<=scoreRea<=1.3113263207824053 ^ 0.06597101090331349<=scoreProtTaxo<=0.3271897037728704 ^ 8.513140588286736E-5<=scorePageRankTopoDiv<=1.0676933935888684E-4  (9)
class DEGRADATION IF : 0.09166984970282113<=scoreRea<=0.11065666703449763 ^ 8.256138978357354<=scoreProtTaxo<=12.978394627069473 ^ 4.006361468932858E-5<=scorePageRankTopoDiv<=4.0878463928647076E-5  (5)
class BIOSYNTHESIS IF : 0.8528028654224418<=scoreRea<=1.9094065395649336 ^ 40.57530437366926<=scoreProtTaxo<=108.87019465254258 ^ 7.171085307698987E-5<=scorePageRankTopoDiv<=7.423092402198566E-5  (14)
class BIOSYNTHESIS IF : 0.40451991747794525<=scoreRea<=0.5 ^ 115.83158598876034<=scoreProtTaxo<=207.2058350485641 ^ 7.608576765126553E-5<=scorePageRankTopoDiv<=1.0389569195115525E-4  (29)
class BIOSYNTHESIS IF : 1.0<=scoreRea<=1.4142135623730951 ^ 3.8990465632701667<=scoreProtTaxo<=10.098108594172102 ^ 1.9631626055723037E-4<=scorePageRankTopoDiv<=3.1452413833726796E-4  (22)
class BIOSYNTHESIS IF : 1.0<=scoreRea<=2.390457218668787 ^ 49.98394804062424<=scoreProtTaxo<=64.39521773703277 ^ 4.2983140265073273E-4<=scorePageRankTopoDiv<=6.314128497589322E-4  (27)
class BIOSYNTHESIS IF : 0.5773502691896257<=scoreRea<=0.6324555320336759 ^ 541.6808091786036<=scoreProtTaxo<=768.5042916331304 ^ 1.5393073014984863E-4<=scorePageRankTopoDiv<=1.886876260565613E-4  (5)
class DETOX IF : scoreRea=1.0 ^ 377.5158590055317<=scoreProtTaxo<=491.60960120855555 ^ 4.210951572447113E-4<=scorePageRankTopoDiv<=4.456494538717275E-4  (8)
class DEGRADATION IF : scoreRea=0.6546536707079771 ^ scoreProtTaxo=0.0 ^ scorePageRankTopoDiv=9.671766544956472E-5  (1)
class DEGRADATION IF : scoreRea=0.5 ^ scoreProtTaxo=0.0 ^ 1.0859097090239445E-4<=scorePageRankTopoDiv<=1.7901793321744563E-4  (14)
class DEGRADATION IF : 0.692820323027551<=scoreRea<=0.7071067811865476 ^ scoreProtTaxo=0.0 ^ 1.072557301339922E-4<=scorePageRankTopoDiv<=1.131934522697916E-4  (6)
class DEGRADATION IF : 0.5773502691896257<=scoreRea<=0.7071067811865476 ^ 80.70483034769947<=scoreProtTaxo<=119.06929966952241 ^ 8.838725777845112E-5<=scorePageRankTopoDiv<=9.297206458788736E-5  (4)
class DEGRADATION IF : scoreRea=0.6546536707079771 ^ scoreProtTaxo=0.0 ^ scorePageRankTopoDiv=8.766640028603569E-5  (5)
class DEGRADATION IF : scoreRea=0.7071067811865476 ^ scoreProtTaxo=0.0 ^ scorePageRankTopoDiv=9.757823187181929E-5  (2)
class DEGRADATION IF : 0.6324555320336759<=scoreRea<=0.6340037731068526 ^ 0.893383640077447<=scoreProtTaxo<=3.502222811304394 ^ 8.188483602428256E-5<=scorePageRankTopoDiv<=9.560425960465856E-5  (6)
class DEGRADATION IF : scoreRea=0.6546536707079771 ^ scoreProtTaxo=0.0 ^ scorePageRankTopoDiv=9.671766544956472E-5  (1)
class DEGRADATION IF : scoreRea=0.6546536707079771 ^ scoreProtTaxo=0.0 ^ scorePageRankTopoDiv=9.671766544956472E-5  (1)
class DEGRADATION IF : scoreRea=0.6546536707079771 ^ scoreProtTaxo=0.0 ^ scorePageRankTopoDiv=9.671766544956472E-5  (1)
class DEGRADATION IF : scoreRea=0.6546536707079771 ^ scoreProtTaxo=0.0 ^ scorePageRankTopoDiv=9.671766544956472E-5  (1)
class DEGRADATION IF : scoreRea=0.6546536707079771 ^ scoreProtTaxo=0.0 ^ scorePageRankTopoDiv=9.671766544956472E-5  (1)
class DEGRADATION IF : scoreRea=0.6546536707079771 ^ scoreProtTaxo=0.0 ^ scorePageRankTopoDiv=9.671766544956472E-5  (1)
class ENERGY IF : scoreRea=0.380058475033046 ^ scoreProtTaxo=0.010500103322752346 ^ scorePageRankTopoDiv=8.047646361772154E-5  (4)
class BIOSYNTHESIS IF : 1.0954451150103324<=scoreRea<=1.4142135623730951 ^ 70.22530852940199<=scoreProtTaxo<=244.7833036744955 ^ 1.4704044926818562E-4<=scorePageRankTopoDiv<=2.4042379903186468E-4  (94)
class BIOSYNTHESIS IF : scoreRea=1.0 ^ 138.75068644666334<=scoreProtTaxo<=144.39855030102657 ^ 8.670863232003228E-5<=scorePageRankTopoDiv<=9.025333891358375E-5 (5)
class BIOSYNTHESIS IF : 1.0<=scoreRea<=1.0327955589886444 ^ 30.727284397150104<=scoreProtTaxo<=129.72023262904227 ^ 9.184702952142395E-5<=scorePageRankTopoDiv<=9.207609793394737E-5  (18)
class DETOX IF : 1.1547005383792515<=scoreRea<=1.2374368670764582 ^ 79.79045207230868<=scoreProtTaxo<=103.88592820586676 ^ 2.9939658600643723E-4<=scorePageRankTopoDiv<=3.192543023177126E-4  (10)
class BIOSYNTHESIS IF : scoreRea=0.7071067811865476 ^ scoreProtTaxo=0.0 ^ 8.00447471683044E-5<=scorePageRankTopoDiv<=9.744629831611159E-5  (31)
class ENERGY IF : scoreRea=0.7071067811865476 ^ scoreProtTaxo=0.0 ^ scorePageRankTopoDiv=2.0800095601150362E-4  (4)
class BIOSYNTHESIS IF : 0.8451542547285166<=scoreRea<=0.8498365855987975 ^ 0.0163659094191017<=scoreProtTaxo<=132.37326865320478 ^ 5.50176610343286E-5<=scorePageRankTopoDiv<=5.878118824107198E-5  (9)
class BIOSYNTHESIS IF : 1.1547005383792515<=scoreRea<=1.2018504251546631 ^ scoreProtTaxo=0.0 ^ 1.3380877114798815E-4<=scorePageRankTopoDiv<=1.92725864781061E-4 (22)
class BIOSYNTHESIS IF : 1.1514524291646675<=scoreRea<=1.766966877363964 ^ 2.735928597404479<=scoreProtTaxo<=3.227339672091711 ^ 9.279920632191633E-5<=scorePageRankTopoDiv<=1.5383984974384253E-4  (13)
class BIOSYNTHESIS IF : scoreRea=1.0 ^ 47.56580340961178<=scoreProtTaxo<=65.97520195284082 ^ 2.246824997052055E-4<=scorePageRankTopoDiv<=2.631955395236231E-4 (17)
class DEGRADATION IF : scoreRea=1.0 ^ scoreProtTaxo=0.0 ^ 3.041377624978212E-4<=scorePageRankTopoDiv<=3.298008762231907E-4  (14)
class BIOSYNTHESIS IF : 0.816496580927726<=scoreRea<=2.14201664188625 ^ 15.742075762667008<=scoreProtTaxo<=86.84275672670222 ^ 9.077163834529936E-5<=scorePageRankTopoDiv<=9.118516690309108E-5  (11)
class DEGRADATION IF : 0.8041247051007595<=scoreRea<=0.816496580927726 ^ 0.0<=scoreProtTaxo<=0.5190213244719686 ^ 1.1821542163722823E-4<=scorePageRankTopoDiv<=1.2267757994319142E-4  (16)
class DEGRADATION IF : 0.6009252125773316<=scoreRea<=0.7071067811865476 ^ 21.637137435383995<=scoreProtTaxo<=34.47987352068098 ^ 7.300051546818665E-5<=scorePageRankTopoDiv<=7.991160054209813E-5  (14)

class DEGRADATION IF : 0.8966167345234256<=scoreRea<=1.4142135623730951 ^ 2.4095196561177734<=scoreProtTaxo<=6.510136530437825 ^ 1.844630092358027E-4<=scorePageRankTopoDiv<=1.9559114106102574E-4 (8)
class DEGRADATION IF : scoreRea=1.854955583040673 ^ scoreProtTaxo=1.1895184214110706 ^ scorePageRankTopoDiv=1.9876999520340293E-4 (3)
class DEGRADATION IF : scoreRea=1.0 ^ scoreProtTaxo=0.0 ^ 2.0021218094743186E-4<=scorePageRankTopoDiv<=2.014356600793569E-4 (6)
class DEGRADATION IF : scoreRea=1.224744871391589 ^ scoreProtTaxo=0.0 ^ 1.4558263294901127E-4<=scorePageRankTopoDiv<=1.6056093176895363E-4 (4)
class DEGRADATION IF : scoreRea=1.0 ^ scoreProtTaxo=35.474289812226985 ^ scorePageRankTopoDiv=1.708260808783563E-4 (4)
class DEGRADATION IF : scoreRea=1.0 ^ scoreProtTaxo=0.0 ^ scorePageRankTopoDiv=1.9567744754793968E-4 (4)
class DEGRADATION IF : scoreRea=1.0 ^ scoreProtTaxo=0.0 ^ 1.6993287011232575E-4<=scorePageRankTopoDiv<=1.7453643114258828E-4 (9)
class DEGRADATION IF : scoreRea=1.0 ^ scoreProtTaxo=0.0 ^ scorePageRankTopoDiv=1.8935139453691235E-4 (3)
class DEGRADATION IF : scoreRea=1.0 ^ scoreProtTaxo=0.0 ^ 1.796932685997016E-4<=scorePageRankTopoDiv<=1.8150128145194246E-4 (9)
class DEGRADATION IF : scoreRea=1.4142135623730951 ^ scoreProtTaxo=0.0 ^ 1.5263370408995772E-4<=scorePageRankTopoDiv<=1.6334478940908766E-4 (9)
class DEGRADATION IF : 1.713913650100261<=scoreRea<=2.0 ^ 6.790781028238273<=scoreProtTaxo<=7.123150982108755 ^ 1.8279934571149197E-4<=scorePageRankTopoDiv<=2.2805075653909617E-4 (11)
class DEGRADATION IF : scoreRea=1.4142135623730951 ^ scoreProtTaxo=0.0 ^ scorePageRankTopoDiv=1.7016811839786362E-4 (4)
class DEGRADATION IF : 1.0<=scoreRea<=1.2649110640673518 ^ 67.41088419467627<=scoreProtTaxo<=69.59562376658221 ^ 1.8621323435720702E-4<=scorePageRankTopoDiv<=1.8902202999632244E-4 (5)
class DEGRADATION IF : 1.224744871391589<=scoreRea<=1.2747548783981961 ^ scoreProtTaxo=0.0 ^ 1.7356471165330003E-4<=scorePageRankTopoDiv<=1.869441828479762E-4 (12)
class DEGRADATION IF : scoreRea=1.0954451150103324 ^ 102.16537911750638<=scoreProtTaxo<=119.36925641394684 ^ 1.1655246691256629E-4<=scorePageRankTopoDiv<=1.2415402697963014E-4 (4)
class DEGRADATION IF : scoreRea=1.0 ^ scoreProtTaxo=0.0 ^ scorePageRankTopoDiv=2.2488847230861333E-4 (2)
class BIOSYNTHESIS IF : scoreRea=1.0 ^ 203.22660501475306<=scoreProtTaxo<=497.4811236160592 ^ scorePageRankTopoDiv=9.184702952142395E-5 (15)
class DEGRADATION IF : scoreRea=0.7071067811865476 ^ scoreProtTaxo=0.0 ^ scorePageRankTopoDiv=1.6567412817495268E-4 (5)
class BIOSYNTHESIS IF : 0.31622776601683794<=scoreRea<=0.4714045207910317 ^ 78.57142496387931<=scoreProtTaxo<=107.58985969873252 ^ 3.8284518337852805E-5<=scorePageRankTopoDiv<=4.917246794022648E-5 (37)
class BIOSYNTHESIS IF : 1.1881770515720091<=scoreRea<=1.224744871391589 ^ scoreProtTaxo=0.0 ^ 9.421736842897945E-5<=scorePageRankTopoDiv<=1.1442075717380128E-4 (10)
class DEGRADATION IF : scoreRea=1.0 ^ scoreProtTaxo=0.0 ^ scorePageRankTopoDiv=4.574313867155294E-4 (2)
class BIOSYNTHESIS IF : 0.34050261230349943<=scoreRea<=0.7977240352174656 ^ 3.0602949519508496<=scoreProtTaxo<=10.185522301794693 ^ 1.715229030060865E-5<=scorePageRankTopoDiv<=3.147447837678314E-5 (22)
class ENERGY IF : 0.14744195615489714<=scoreRea<=0.32732683535398854 ^ 1.0121983138172692<=scoreProtTaxo<=1.9978761838910137 ^ 1.2608413347989437E-4<=scorePageRankTopoDiv<=1.890485460452404E-4 (11)
class BIOSYNTHESIS IF : scoreRea=1.4960909156069733 ^ scoreProtTaxo=0.13138491890319684 ^ scorePageRankTopoDiv=1.0996690323666944E-4 (4)
class BIOSYNTHESIS IF : 0.8498365855987975<=scoreRea<=1.0741723110591495 ^ 0.3514065322154995<=scoreProtTaxo<=0.49235956602335174 ^ 8.767676776912525E-5<=scorePageRankTopoDiv<=1.3469499829656941E-4 (18)
class DEGRADATION IF : 0.16666666666666666<=scoreRea<=0.3535533905932738 ^ 120.44252652744328<=scoreProtTaxo<=169.67173104287173 ^ 9.357090688646382E-5<=scorePageRankTopoDiv<=1.008230936271939E-4 (7)
class BIOSYNTHESIS IF : 0.8660254037844386<=scoreRea<=1.2649110640673518 ^ 683.722406473152<=scoreProtTaxo<=2285.021645022659 ^ 9.184702952142395E-5<=scorePageRankTopoDiv<=3.3611575163561925E-4 (43)
class BIOSYNTHESIS IF : 0.9274777915203366<=scoreRea<=1.0954451150103324 ^ 0.0<=scoreProtTaxo<=0.3136103074603237 ^ 8.476168618352132E-5<=scorePageRankTopoDiv<=8.770529561401202E-5 (22)
class BIOSYNTHESIS IF : 0.7001400420140049<=scoreRea<=0.7745966692414834 ^ 0.0<=scoreProtTaxo<=0.011411785960777153 ^ 1.1399210988284985E-4<=scorePageRankTopoDiv<=1.2369853738259412E-4 (38)
class BIOSYNTHESIS IF : 0.8498365855987975<=scoreRea<=0.9274777915203366 ^ 0.0<=scoreProtTaxo<=0.17684850252398204 ^ 1.1884249895025044E-4<=scorePageRankTopoDiv<=1.336146798704194E-4 (11)
class DEGRADATION IF : 1.889822365046136<=scoreRea<=2.0 ^ scoreProtTaxo=0.0 ^ 1.2475533424711406E-4<=scorePageRankTopoDiv<=1.280016077304211E-4 (2)
class BIOSYNTHESIS IF : 0.31622776601683794<=scoreRea<=0.6183469424008422 ^ 10.926420578880442<=scoreProtTaxo<=12.156635704251286 ^ 2.5481468240134802E-5<=scorePageRankTopoDiv<=3.212687966439753E-5 (13)
class BIOSYNTHESIS IF : scoreRea=1.4142135623730951 ^ scoreProtTaxo=0.0 ^ 9.184702952142395E-5<=scorePageRankTopoDiv<=1.0101442288769912E-4 (5)
class BIOSYNTHESIS IF : scoreRea=1.4142135623730951 ^ scoreProtTaxo=35.24228074716164 ^ scorePageRankTopoDiv=9.184702952142395E-5 (5)
class BIOSYNTHESIS IF : scoreRea=1.3981241472851111 ^ scoreProtTaxo=21.378116572226567 ^ scorePageRankTopoDiv=9.381569842146049E-5 (2)
class BIOSYNTHESIS IF : 0.11826247919781653<=scoreRea<=2.0225995873897267 ^ 10.776152121488197<=scoreProtTaxo<=105.49746842477356 ^ 9.396036002192474E-5<=scorePageRankTopoDiv<=9.557427168280585E-5 (19)
class BIOSYNTHESIS IF : 0.4472135954999579<=scoreRea<=1.651445647689541 ^ 15.874507866387544<=scoreProtTaxo<=80.22722087675474 ^ 8.395834738030331E-5<=scorePageRankTopoDiv<=9.05851900355019E-5 (56)
class DEGRADATION IF : scoreRea=1.2747548783981961 ^ scoreProtTaxo=0.0 ^ scorePageRankTopoDiv=1.0154910594416097E-4 (4)
class BIOSYNTHESIS IF : 0.5773502691896257<=scoreRea<=1.0 ^ 202.1185360063624<=scoreProtTaxo<=212.76058532225056 ^ 4.679126315010273E-5<=scorePageRankTopoDiv<=6.290816188871419E-5 (10)
class BIOSYNTHESIS IF : 0.2231780211373287<=scoreRea<=0.5686020318963798 ^ 0.7364232486787674<=scoreProtTaxo<=131.3492737973759 ^ 5.735621962844952E-5<=scorePageRankTopoDiv<=5.839220869253309E-5 (29)
class DEGRADATION IF : 0.7453559924999298<=scoreRea<=0.7453559924999299 ^ 38.17176001418311<=scoreProtTaxo<=42.46577637766384 ^ 5.201708028526716E-5<=scorePageRankTopoDiv<=6.246401784808223E-5 (7)
class BIOSYNTHESIS IF : scoreRea=1.0 ^ 105.57777543909937<=scoreProtTaxo<=196.07813179808738 ^ 1.695742324714891E-4<=scorePageRankTopoDiv<=2.0341900322563264E-4 (17)
class DEGRADATION IF : 0.23570226039551584<=scoreRea<=0.2581988897471611 ^ 0.0<=scoreProtTaxo<=15.067688831635602 ^ 1.1838338946480182E-4<=scorePageRankTopoDiv<=1.2540296301974467E-4 (11)
class BIOSYNTHESIS IF : scoreRea=0.08770580193070292 ^ scoreProtTaxo=0.0 ^ scorePageRankTopoDiv=1.0377721218307198E-4 (4)
class OTHER IF : scoreRea=1.7320508075688772 ^ scoreProtTaxo=113.37384803240559 ^ scorePageRankTopoDiv=9.025333891358375E-5 (2)

class BIOSYNTHESIS IF : scoreRea=0.4472135954999579 ^ scoreProtTaxo=0.0 ^ 8.060115160269311E-5<=scorePageRankTopoDiv<=9.008636540561074E-5 (7)
class DEGRADATION IF : scoreRea=1.0 ^ 459.13672922216<=scoreProtTaxo<=481.6843064871512 ^ 1.7909922888682906E-4<=scorePageRankTopoDiv<=3.40464408328133E-4 (6)
class BIOSYNTHESIS IF : 1.8257418583505536<=scoreRea<=3.265986323710904 ^ 33.12248386674865<=scoreProtTaxo<=136.42726041965906 ^ 2.1269607686288917E-4<=scorePageRankTopoDiv<=2.3234515563282242E-4 (20)
class BIOSYNTHESIS IF : 0.7745966692414834<=scoreRea<=0.8177595307636584 ^ 0.0<=scoreProtTaxo<=2.037106752619959 ^ 5.453708571594084E-5<=scorePageRankTopoDiv<=5.7664954295475665E-5 (10)
class ENERGY IF : 0.08099238707340584<=scoreRea<=0.14201432049934526 ^ 22.740488618689128<=scoreProtTaxo<=74.32767683331349 ^ 3.515238536038338E-5<=scorePageRankTopoDiv<=4.331378370561842E-5 (32)
class ENERGY IF : 0.4714045207910317<=scoreRea<=0.7071067811865476 ^ 5.131234358813483<=scoreProtTaxo<=6.047393765526435 ^ 7.701995272183148E-5<=scorePageRankTopoDiv<=1.2947129383306984E-4 (14)
class ENERGY IF : 0.1851640199545103<=scoreRea<=0.21908902300206645 ^ 1.0815175480053552<=scoreProtTaxo<=1.3687047831034047 ^ 3.912445526789275E-5<=scorePageRankTopoDiv<=4.770101382830417E-5 (7)
class ENERGY IF : scoreRea=0.23735633163877065 ^ scoreProtTaxo=8.146624267430086 ^ scorePageRankTopoDiv=7.71820203608201E-5 (2)
class ENERGY IF : 0.14638501094227999<=scoreRea<=0.3039305703971088 ^ 0.005236462280125203<=scoreProtTaxo<=0.041096692455940075 ^ 7.542658385848098E-5<=scorePageRankTopoDiv<=8.811181365903088E-5 (9)
class ENERGY IF : scoreRea=0.24618298195866548 ^ scoreProtTaxo=5.306184827620615 ^ scorePageRankTopoDiv=5.090704620784785E-5 (8)
class ENERGY IF : 0.16666666666666666<=scoreRea<=0.20701966780270625 ^ 0.058119499839866515<=scoreProtTaxo<=0.16287691064839444 ^ 7.259786429147831E-5<=scorePageRankTopoDiv<=1.0666929785613536E-4 (15)
class ENERGY IF : scoreRea=0.37399695868987537 ^ scoreProtTaxo=2.176539670611231 ^ scorePageRankTopoDiv=6.928775513126102E-5 (3)
class ENERGY IF : 0.39749048208014426<=scoreRea<=0.49065338146265813 ^ 0.01202320036306871<=scoreProtTaxo<=0.7345465850667467 ^ 6.0236816739474416E-5<=scorePageRankTopoDiv<=6.0598885144221155E-5 (8)
class ENERGY IF : scoreRea=0.3370999312316211 ^ scoreProtTaxo=0.0 ^ scorePageRankTopoDiv=6.778651896076734E-5 (4)
class ENERGY IF : scoreRea=0.19364916731037085 ^ scoreProtTaxo=4.743266578047228 ^ scorePageRankTopoDiv=6.766148967933868E-5 (2)
class ENERGY IF : scoreRea=0.2683281572999748 ^ scoreProtTaxo=1.3245830702395656 ^ scorePageRankTopoDiv=5.84215720463948E-5 (4)
class ENERGY IF : 0.14285714285714285<=scoreRea<=0.21320071635561044 ^ 0.4767035715081458<=scoreProtTaxo<=0.7353385010338678 ^ 6.663890312950396E-5<=scorePageRankTopoDiv<=9.013481199149459E-5 (6)
class ENERGY IF : scoreRea=0.20100756305184242 ^ scoreProtTaxo=0.0 ^ scorePageRankTopoDiv=6.164581976670261E-5 (2)
class ENERGY IF : 0.11826247919781653<=scoreRea<=0.1889822365046136 ^ 35.1112974762364<=scoreProtTaxo<=84.87073599036444 ^ 6.42247008249192E-5<=scorePageRankTopoDiv<=8.085551988877341E-5 (29)
class ENERGY IF : 0.18569533817705186<=scoreRea<=0.20788767860257112 ^ 14.185160517004936<=scoreProtTaxo<=56.017774202359035 ^ 5.557479307499183E-5<=scorePageRankTopoDiv<=6.377510375064118E-5 (7)
class BIOSYNTHESIS IF : scoreRea=0.24743582965269675 ^ scoreProtTaxo=45.577594029084196 ^ scorePageRankTopoDiv=6.303181454151838E-5 (4)
class DEGRADATION IF : 0.7071067811865476<=scoreRea<=1.0 ^ 333.2922381910363<=scoreProtTaxo<=335.05701833979776 ^ 1.2080198612630699E-4<=scorePageRankTopoDiv<=1.4530708930785835E-4 (9)
class DEGRADATION IF : 1.0<=scoreRea<=1.0954451150103321 ^ 54.927732910784854<=scoreProtTaxo<=69.7429349678668 ^ 1.0547963298888047E-4<=scorePageRankTopoDiv<=1.1900372627817683E-4 (6)
class DEGRADATION IF : scoreRea=0.5 ^ scoreProtTaxo=71.88664045309517 ^ scorePageRankTopoDiv=5.046510182258221E-5 (5)
class OTHER IF : scoreRea=1.0 ^ scoreProtTaxo=0.0 ^ scorePageRankTopoDiv=1.2181717835020451E-4 (3)
class BIOSYNTHESIS IF : 0.4330127018922193<=scoreRea<=0.7071067811865476 ^ 2.368391006896105<=scoreProtTaxo<=45.87562987521707 ^ 3.4589070071606805E-5<=scorePageRankTopoDiv<=3.918752838690245E-5 (21)
class DEGRADATION IF : scoreRea=0.5773502691896257 ^ 16.241979271931093<=scoreProtTaxo<=16.967711271902253 ^ 1.3994040384206641E-4<=scorePageRankTopoDiv<=1.4353561834606595E-4 (9)
class DEGRADATION IF : 0.5773502691896257<=scoreRea<=0.7071067811865476 ^ 53.13945655838685<=scoreProtTaxo<=156.685537438235 ^ 1.7250657726510578E-4<=scorePageRankTopoDiv<=1.7700450746818247E-4 (11)
class BIOSYNTHESIS IF : 0.816496580927726<=scoreRea<=0.8563488385776752 ^ scoreProtTaxo=0.0 ^ 1.1588081705557356E-4<=scorePageRankTopoDiv<=1.1676393832346643E-4 (9)
class BIOSYNTHESIS IF : 0.8660254037844386<=scoreRea<=1.0 ^ 55.5178349721961<=scoreProtTaxo<=165.08662498808434 ^ 1.1966643069700278E-4<=scorePageRankTopoDiv<=1.276882886463082E-4 (12)
class BIOSYNTHESIS IF : 1.5491933384829668<=scoreRea<=3.7416573867739413 ^ 103.17385445279729<=scoreProtTaxo<=1032.1323526036228 ^ 1.6550584019747294E-4<=scorePageRankTopoDiv<=2.0794659757250114E-4 (35)
class DEGRADATION IF : 0.09421114395319916<=scoreRea<=0.2401922307076307 ^ 105.54140231826986<=scoreProtTaxo<=158.4040314229278 ^ 1.85814553170896E-5<=scorePageRankTopoDiv<=4.761594272264702E-5 (16)
class BIOSYNTHESIS IF : 0.816496580927726<=scoreRea<=2.6111648393354674 ^ 0.0<=scoreProtTaxo<=207.6349165468604 ^ 4.100990388848989E-4<=scorePageRankTopoDiv<=4.219107144029883E-4 (32)
class OTHER IF : 0.15075567228888181<=scoreRea<=0.2041241452319315 ^ 7.058491402900568<=scoreProtTaxo<=24.85219968434863 ^ 7.863745812978191E-5<=scorePageRankTopoDiv<=9.135602271849298E-5 (3)
class DEGRADATION IF : 0.816496580927726<=scoreRea<=0.8320502943378437 ^ 54.93483103796865<=scoreProtTaxo<=159.97631877899414 ^ 1.7376808566874698E-4<=scorePageRankTopoDiv<=1.81989932974737E-4 (20)
class BIOSYNTHESIS IF : 0.5720775535473553<=scoreRea<=1.6858544608470492 ^ 1.2185788425532535<=scoreProtTaxo<=1.254188385269777 ^ 1.3565745805312304E-4<=scorePageRankTopoDiv<=1.5188589615391725E-4 (16)
class BIOSYNTHESIS IF : 0.30151134457776363<=scoreRea<=0.48507125007266594 ^ 43.97644267557982<=scoreProtTaxo<=237.94152843872973 ^ 1.5181458419799012E-4<=scorePageRankTopoDiv<=1.622561555051185E-4 (24)
class OTHER IF : scoreRea=0.2773500981126146 ^ scoreProtTaxo=10.349540531628243 ^ scorePageRankTopoDiv=4.731675223938819E-5 (7)
class BIOSYNTHESIS IF : scoreRea=1.1677484162422844 ^ scoreProtTaxo=0.0 ^ scorePageRankTopoDiv=8.279653168918027E-5 (5)
class DEGRADATION IF : 0.18257418583505536<=scoreRea<=0.2581988897471611 ^ 20.29655143121609<=scoreProtTaxo<=27.408620731817063 ^ 6.379348993329522E-5<=scorePageRankTopoDiv<=6.679800841717476E-5 (11)
class BIOSYNTHESIS IF : 0.4264014327112209<=scoreRea<=0.42857142857142855 ^ 0.0<=scoreProtTaxo<=16.493671914133294 ^ 1.3819175744626106E-4<=scorePageRankTopoDiv<=1.5471251659915627E-4 (9)

```
class BIOSYNTHESIS IF : scoreRea=0.3535533905932738 ^ scoreProtTaxo=22.65668793082446 ^ scorePageRankTopoDiv=1.6950971007434574E-4  (13)
class OTHER IF : scoreRea=0.816496580927726 ^ scoreProtTaxo=109.62502934472009 ^ scorePageRankTopoDiv=3.6792614690010723E-4  (4)
class ENERGY IF : 0.31622776601683794<=scoreRea<=0.32714432804381227 ^ 0.6004119665829322<=scoreProtTaxo<=4.484080035378623 ^ 3.226244283908063E-5<=scorePageRankTopoDiv<=3.72149218380753E-5  (9)
class DEGRADATION IF : 0.07147416898918632<=scoreRea<=0.5052911526399113 ^ scoreProtTaxo=0.0 ^ 1.7203438390870207E-5<=scorePageRankTopoDiv<=2.2300141489819972E-5 (22)
class OTHER IF : scoreRea=1.0 ^ 294.5535714285243<=scoreProtTaxo<=325.270349094411 ^ 2.0341900322563264E-4<=scorePageRankTopoDiv<=2.0507683798991577E-4  (10)
class BIOSYNTHESIS IF : 1.1952286093343936<=scoreRea<=1.61245154965971 ^ 0.16194510804587167<=scoreProtTaxo<=54.44846840950304 ^ 3.3376497294104843E-4<=scorePageRankTopoDiv<=3.5209617300788025E-4  (15)
class BIOSYNTHESIS IF : 0.18257418583505536<=scoreRea<=0.2041241452319315 ^ 0.7532020994315557<=scoreProtTaxo<=32.6965054097352 ^ 2.312808880264647E-5<=scorePageRankTopoDiv<=3.4896462935919714E-5  (23)
class BIOSYNTHESIS IF : 1.0954451150103321<=scoreRea<=1.0954451150103324 ^ scoreProtTaxo=47.31651754796645 ^ scorePageRankTopoDiv=1.1230007606831941E-4  (5)
class BIOSYNTHESIS IF : 0.3535533905932738<=scoreRea<=1.0 ^ 35.772357723564<=scoreProtTaxo<=322.6091560103118 ^ 1.3198585967944532E-4<=scorePageRankTopoDiv<=1.3306746816423162E-4  (18)
class BIOSYNTHESIS IF : scoreRea=0.7071067811865476 ^ scoreProtTaxo=0.0 ^ 1.293202030337335E-4<=scorePageRankTopoDiv<=1.333663591583185E-4  (14)
class BIOSYNTHESIS IF : 0.7071067811865476<=scoreRea<=0.8451542547285166 ^ 9.263985137001498<=scoreProtTaxo<=24.028327953004528 ^ 2.136764660064425E-4<=scorePageRankTopoDiv<=2.5281930371988685E-4  (9)
class BIOSYNTHESIS IF : 0.23570226039551584<=scoreRea<=0.25 ^ 0.0<=scoreProtTaxo<=0.8988650644845613 ^ 1.0711977779640557E-4<=scorePageRankTopoDiv<=1.1719969391414448E-4  (7)
class DEGRADATION IF : 0.04879500364742666<=scoreRea<=0.09258200997725514 ^ 25.472692692038926<=scoreProtTaxo<=108.39077309645593 ^ 1.806864911473614E-5<=scorePageRankTopoDiv<=2.7793795106385645E-5  (25)
class OTHER IF : 0.8528028654224418<=scoreRea<=1.0 ^ 10.482301979492092<=scoreProtTaxo<=28.17578747790379 ^ 9.184702952142395E-5<=scorePageRankTopoDiv<=9.351198002934252E-5  (11)
class BIOSYNTHESIS IF : 0.5773502691896257<=scoreRea<=0.7071067811865476 ^ 0.0<=scoreProtTaxo<=30.48079304932964 ^ 5.3154500055403816E-5<=scorePageRankTopoDiv<=5.5882189113535004E-5  (15)
class BIOSYNTHESIS IF : 0.6793662204867574<=scoreRea<=0.775791113542719 ^ 0.0<=scoreProtTaxo<=0.0709936559865042 ^ 1.3585363833144514E-4<=scorePageRankTopoDiv<=1.419800023207035E-4  (24)
class BIOSYNTHESIS IF : scoreRea=1.0 ^ scoreProtTaxo=0.0 ^ 1.33480015484179E-4<=scorePageRankTopoDiv<=1.3671629379591788E-4  (8)
class BIOSYNTHESIS IF : scoreRea=1.0 ^ scoreProtTaxo=0.0 ^ 1.6552917690418017E-4<=scorePageRankTopoDiv<=1.6968382380836306E-4  (15)
class BIOSYNTHESIS IF : 0.7071067811865476<=scoreRea<=0.816496580927726 ^ 78.02205121755242<=scoreProtTaxo<=310.4692444030603 ^ 1.4006546779844433E-4<=scorePageRankTopoDiv<=1.5967022554404776E-4  (10)
class BIOSYNTHESIS IF : scoreRea=1.0 ^ scoreProtTaxo=27.6271696741116 ^ scorePageRankTopoDiv=1.630244314637863E-4  (4)
class BIOSYNTHESIS IF : 0.8498365855987975<=scoreRea<=0.8819171036881968 ^ 0.0<=scoreProtTaxo<=0.02987286337893175 ^ 1.6014420706100203E-4<=scorePageRankTopoDiv<=1.7597335020488872E-4  (6)
class BIOSYNTHESIS IF : scoreRea=1.0 ^ scoreProtTaxo=0.0 ^ scorePageRankTopoDiv=1.413963764486109E-4  (1)
class BIOSYNTHESIS IF : 0.7071067811865476<=scoreRea<=0.816496580927726 ^ 3.6899216336007026<=scoreProtTaxo<=4.0834889947534485 ^ 8.267082416947604E-5<=scorePageRankTopoDiv<=2.7107121994508987E-4  (7)
class BIOSYNTHESIS IF : scoreRea=1.0 ^ scoreProtTaxo=0.0 ^ 1.4183238468692228E-4<=scorePageRankTopoDiv<=1.4208373799633385E-4  (7)
class BIOSYNTHESIS IF : 0.6123724356957945<=scoreRea<=0.8498365855987975 ^ 2.1684886029206134<=scoreProtTaxo<=2.2017607556010725 ^ 1.518898979278972E-4<=scorePageRankTopoDiv<=2.6238879696150873E-4  (7)
class BIOSYNTHESIS IF : scoreRea=0.8660254037844386 ^ scoreProtTaxo=0.0 ^ 1.4138985419279236E-4<=scorePageRankTopoDiv<=1.5691800610686908E-4  (16)
class BIOSYNTHESIS IF : scoreRea=1.0 ^ scoreProtTaxo=0.0 ^ 1.426170215875551E-4<=scorePageRankTopoDiv<=1.4491894953559909E-4  (6)
class BIOSYNTHESIS IF : 0.7071067811865476<=scoreRea<=1.0 ^ scoreProtTaxo=0.0 ^ 1.570005579549546E-4<=scorePageRankTopoDiv<=1.5739447872831032E-4  (8)
class BIOSYNTHESIS IF : 0.8660254037844386<=scoreRea<=1.0 ^ 12.33487673829909<=scoreProtTaxo<=220.12790894432126 ^ 1.4036841679306012E-4<=scorePageRankTopoDiv<=1.60770434204676E-4  (27)
class DEGRADATION IF : 0.7001400420140049<=scoreRea<=0.9309493362512627 ^ 12.36247558554131<=scoreProtTaxo<=116.8689224308081 ^ 1.1254972809590294E-4<=scorePageRankTopoDiv<=1.1715656138055222E-4  (34)
class DEGRADATION IF : 0.3723646897664626<=scoreRea<=0.5345224838248488 ^ scoreProtTaxo=0.0 ^ 5.9327432063423745E-5<=scorePageRankTopoDiv<=6.385494276414651E-5 (19)
class OTHER IF : scoreRea=0.7071067811865476 ^ scoreProtTaxo=0.0 ^ scorePageRankTopoDiv=5.673579070415349E-5  (1)
class OTHER IF : scoreRea=0.18156825980064073 ^ scoreProtTaxo=21.804187367254194 ^ scorePageRankTopoDiv=4.6880309815237896E-5  (2)
class OTHER IF : scoreRea=1.0801234497346432 ^ scoreProtTaxo=80.10792330008738 ^ scorePageRankTopoDiv=7.039467061986714E-5  (1)
class OTHER IF : scoreRea=0.5 ^ scoreProtTaxo=193.6710726321274 ^ scorePageRankTopoDiv=5.787400974552743E-5  (9)
class OTHER IF : scoreRea=0.4472135954999579 ^ scoreProtTaxo=0.0 ^ scorePageRankTopoDiv=7.735966100425115E-5  (3)
class OTHER IF : 0.3396831102433787<=scoreRea<=0.3922322702763681 ^ 36.03337000244279<=scoreProtTaxo<=64.44969605773143 ^ 3.8086671000353744E-5<=scorePageRankTopoDiv<=3.8884620735136614E-5  (7)
class OTHER IF : scoreRea=0.6590820436573076 ^ scoreProtTaxo=16.279956356046515 ^ scorePageRankTopoDiv=7.885620403142671E-5  (1)
class OTHER IF : scoreRea=0.7745966692414834 ^ scoreProtTaxo=53.734496423617 ^ scorePageRankTopoDiv=7.774312429831847E-5  (3)
class OTHER IF : scoreRea=1.044465935734187 ^ scoreProtTaxo=0.0 ^ scorePageRankTopoDiv=6.556201102852384E-5  (3)
class OTHER IF : scoreRea=0.6546536707079771 ^ scoreProtTaxo=0.0 ^ scorePageRankTopoDiv=7.51875193871095E-5  (3)
class OTHER IF : scoreRea=0.7071067811865476 ^ scoreProtTaxo=158.79296259232737 ^ scorePageRankTopoDiv=5.673579070415349E-5  (3)
class OTHER IF : 0.3922322702763681<=scoreRea<=0.408248290463863 ^ 8.609939537894114<=scoreProtTaxo<=23.547981101223865 ^ 3.8884620735136614E-5<=scorePageRankTopoDiv<=4.7523107196888775E-5  (9)
class OTHER IF : 0.9554425212028317<=scoreRea<=1.044465935734187 ^ 0.0<=scoreProtTaxo<=0.0875166975021412 ^ 6.728558346708853E-5<=scorePageRankTopoDiv<=6.881127796865302E-5  (12)
class OTHER IF : 0.3872983346207417<=scoreRea<=0.408248290463863 ^ 40.06437409876283<=scoreProtTaxo<=47.54718111935941 ^ 6.086380563487903E-5<=scorePageRankTopoDiv<=7.323460746215443E-5  (7)
class OTHER IF : scoreRea=0.40206235255037975 ^ scoreProtTaxo=0.18178615015434169 ^ scorePageRankTopoDiv=5.203961345268193E-5  (2)
```

```
        class OTHER IF : scoreRea=0.5462951617043315 ^ scoreProtTaxo=0.5947363999761335 ^ scorePageRankTopoDiv=4.583609822462573E-5  (4)
        class OTHER IF : 0.32025630761017426<=scoreRea<=0.49507377148833714 ^ 39.60578401929289<=scoreProtTaxo<=96.40149730579888 ^ 4.960844882259927E-
5<=scorePageRankTopoDiv<=5.273409131160857E-5  (6)
        class OTHER IF : scoreRea=0.4714045207910317 ^ scoreProtTaxo=18.82846753284995 ^ scorePageRankTopoDiv=5.638905553981655E-5  (2)
        class OTHER IF : scoreRea=0.8770580193070292 ^ scoreProtTaxo=61.90623858885454 ^ scorePageRankTopoDiv=5.637178992061003E-5  (3)
        class OTHER IF : 0.25677629550654774<=scoreRea<=0.5773502691896257 ^ 30.835777491294994<=scoreProtTaxo<=32.56157357141713 ^ 6.448584985880469E-
5<=scorePageRankTopoDiv<=6.629876994896196E-5  (16)
        class DEGRADATION IF : scoreRea=0.7071067811865476 ^ scoreProtTaxo=0.08182885492431484 ^ scorePageRankTopoDiv=5.673579070415349E-5  (1)
        class BIOSYNTHESIS IF : scoreRea=1.0 ^ scoreProtTaxo=0.0 ^ scorePageRankTopoDiv=2.0507683798991577E-4  (2)
        class OTHER IF : 0.24397501823713327<=scoreRea<=0.44781107551989907 ^ 29.521443347764954<=scoreProtTaxo<=38.509613026640984 ^ 2.0674138746251432E-
5<=scorePageRankTopoDiv<=2.152043334018977E-5  (18)
        class BIOSYNTHESIS IF : scoreRea=0.5547001962252291 ^ scoreProtTaxo=27.102885552202117 ^ scorePageRankTopoDiv=8.236502969785701E-5  (2)
        class DEGRADATION IF : 0.28867513459481287<=scoreRea<=0.2988071523335984 ^ 21.952190576265732<=scoreProtTaxo<=30.177599308096063 ^ 2.4269456232019226E-
5<=scorePageRankTopoDiv<=4.027485301057047E-5  (18)
        class BIOSYNTHESIS IF : 0.5504818825631803<=scoreRea<=0.7933470778425575 ^ 10.84536134295424<=scoreProtTaxo<=81.22294317160403 ^ 4.197659114448056E-
5<=scorePageRankTopoDiv<=5.060159247463918E-5  (37)
        class DETOX IF : scoreRea=0.7071067811865476 ^ scoreProtTaxo=0.0 ^ scorePageRankTopoDiv=6.49456574064399E-5  (1)
        class DETOX IF : scoreRea=0.4714045207910317 ^ scoreProtTaxo=5.626160592721068 ^ scorePageRankTopoDiv=1.8579819527032382E-4  (3)
        class DETOX IF : scoreRea=0.5773502691896257 ^ scoreProtTaxo=63.12025864186631 ^ scorePageRankTopoDiv=1.7909317517514394E-4  (3)
        class DETOX IF : scoreRea=1.224744871391589 ^ scoreProtTaxo=0.0 ^ scorePageRankTopoDiv=1.7121731471953552E-4  (2)
        class DETOX IF : scoreRea=1.4142135623730951 ^ scoreProtTaxo=0.0 ^ scorePageRankTopoDiv=1.653816457352169E-4  (3)
        class DETOX IF : scoreRea=1.0 ^ scoreProtTaxo=0.0 ^ scorePageRankTopoDiv=1.5033597960863803E-4  (3)
        class DETOX IF : scoreRea=1.5 ^ scoreProtTaxo=0.0 ^ scorePageRankTopoDiv=1.3793280935725012E-4  (3)
        class DETOX IF : scoreRea=0.3333333333333333 ^ scoreProtTaxo=3.978296307157593 ^ scorePageRankTopoDiv=1.313791638078683E-4  (3)
        class DETOX IF : scoreRea=0.21821789023599236 ^ scoreProtTaxo=0.0 ^ scorePageRankTopoDiv=1.1724400452715474E-4  (6)
        class DETOX IF : scoreRea=1.4142135623730951 ^ scoreProtTaxo=0.0 ^ scorePageRankTopoDiv=1.0815609884967573E-4  (5)
        class DETOX IF : 0.28867513459481287<=scoreRea<=0.3086066999241838 ^ scoreProtTaxo=0.0 ^ 1.1754985763092038E-4<=scorePageRankTopoDiv<=2.2198230760370834E-4  (13)
        class DETOX IF : scoreRea=1.1677484162422844 ^ scoreProtTaxo=0.0 ^ scorePageRankTopoDiv=1.0311728461534989E-4  (2)
        class DETOX IF : scoreRea=1.5491933384829668 ^ scoreProtTaxo=0.0 ^ scorePageRankTopoDiv=6.927764921033681E-5  (1)
        class DETOX IF : scoreRea=0.816496580927726 ^ scoreProtTaxo=136.4722494436797 ^ scorePageRankTopoDiv=9.957483086475267E-5  (2)
        class DETOX IF : scoreRea=1.0274023338281626 ^ scoreProtTaxo=35.59127779829795 ^ scorePageRankTopoDiv=9.314989437145699E-5  (2)
        class DETOX IF : scoreRea=1.1649647450214349 ^ scoreProtTaxo=145.869357387843 ^ scorePageRankTopoDiv=5.289018487006144E-5  (2)
        class DETOX IF : scoreRea=0.21821789023599236 ^ scoreProtTaxo=0.0 ^ scorePageRankTopoDiv=6.327543175025379E-5  (6)
        class DETOX IF : scoreRea=0.8660254037844386 ^ scoreProtTaxo=0.0 ^ scorePageRankTopoDiv=8.677801489810921E-5  (4)
        class DETOX IF : scoreRea=1.1547005383792515 ^ scoreProtTaxo=10.736953861194982 ^ scorePageRankTopoDiv=6.592735397720618E-5  (2)
        class DETOX IF : scoreRea=0.9428090415820634 ^ scoreProtTaxo=28.33725463058259 ^ scorePageRankTopoDiv=7.382789095532152E-5  (4)
        class DETOX IF : scoreRea=0.8257228238447705 ^ scoreProtTaxo=0.0 ^ scorePageRankTopoDiv=7.291493120905716E-5  (2)
        class BIOSYNTHESIS IF : 0.6488856845230502<=scoreRea<=0.7071067811865476 ^ scoreProtTaxo=0.0 ^ 6.164120344039986E-5<=scorePageRankTopoDiv<=6.350873347874623E-5
(19)
        class BIOSYNTHESIS IF : scoreRea=1.0 ^ scoreProtTaxo=0.0 ^ scorePageRankTopoDiv=2.515433611321339E-4  (6)
        class OTHER IF : scoreRea=2.1032382440206603 ^ scoreProtTaxo=0.032200144283896934 ^ scorePageRankTopoDiv=2.089653430203537E-4  (6)
        class BIOSYNTHESIS IF : 3.618347271170902<=scoreRea<=6.323973992579629 ^ 4.17119000018096<=scoreProtTaxo<=29.932079089972547 ^ 2.7861408614411143E-
4<=scorePageRankTopoDiv<=4.491850260334036E-4  (11)
        class BIOSYNTHESIS IF : scoreRea=3.7859388972001824 ^ scoreProtTaxo=1.5438918689156014 ^ scorePageRankTopoDiv=5.0759790640253544E-5  (7)
        class BIOSYNTHESIS IF : 0.6454972243679028<=scoreRea<=1.860521018838127 ^ 1.786383293240843<=scoreProtTaxo<=2.1278986237028517 ^ 1.1603744675636554E-
4<=scorePageRankTopoDiv<=1.221292855312842E-4  (26)
        class DEGRADATION IF : 0.4472135954999579<=scoreRea<=1.0 ^ 86.77558163807879<=scoreProtTaxo<=152.79187765288137 ^ 1.3520119512554292E-
4<=scorePageRankTopoDiv<=1.399925842034762E-4  (20)
        class BIOSYNTHESIS IF : scoreRea=0.7071067811865476 ^ scoreProtTaxo=168.14999256616102 ^ scorePageRankTopoDiv=1.3114233478901078E-4  (7)
        class DEGRADATION IF : scoreRea=0.5773502691896257 ^ 35.276659918992415<=scoreProtTaxo<=40.259317459798886 ^ 1.1001563219750556E-
4<=scorePageRankTopoDiv<=1.1076060971949355E-4  (12)
        class DEGRADATION IF : scoreRea=0.7071067811865476 ^ scoreProtTaxo=0.0 ^ scorePageRankTopoDiv=1.3535170583744646E-4  (1)
        class DEGRADATION IF : scoreRea=1.0 ^ scoreProtTaxo=0.0 ^ scorePageRankTopoDiv=1.3276998503471255E-4  (4)
        class DEGRADATION IF : scoreRea=1.0 ^ scoreProtTaxo=0.0 ^ scorePageRankTopoDiv=1.378098766816995E-4  (4)
        class DEGRADATION IF : scoreRea=0.7071067811865476 ^ scoreProtTaxo=0.0 ^ 1.4943057764010143E-4<=scorePageRankTopoDiv<=1.5357082380142837E-4  (9)
        class DEGRADATION IF : scoreRea=0.7071067811865476 ^ scoreProtTaxo=3.675466178295334 ^ scorePageRankTopoDiv=1.216405836434982E-4  (5)
        class DEGRADATION IF : scoreRea=0.6454972243679028 ^ scoreProtTaxo=0.08101161375304392 ^ scorePageRankTopoDiv=1.474440601213266E-4  (4)
        class DEGRADATION IF : scoreRea=0.9274777915203366 ^ scoreProtTaxo=0.9481027057697574 ^ scorePageRankTopoDiv=1.3124640696624947E-4  (2)
        class DEGRADATION IF : scoreRea=0.6236095644623235 ^ scoreProtTaxo=4.634759947408514 ^ scorePageRankTopoDiv=1.4429449547506584E-4  (2)
        class DEGRADATION IF : scoreRea=0.816496580927726 ^ scoreProtTaxo=37.27112086645609 ^ scorePageRankTopoDiv=1.422812003992657E-4  (1)
        class DEGRADATION IF : scoreRea=0.7071067811865476 ^ scoreProtTaxo=0.0 ^ scorePageRankTopoDiv=1.437745131890555E-4  (3)
        class DEGRADATION IF : scoreRea=0.7071067811865476 ^ scoreProtTaxo=0.0 ^ scorePageRankTopoDiv=1.270951021637754E-4  (5)
        class DEGRADATION IF : 0.6859943405700354<=scoreRea<=0.816496580927726 ^ 37.27112086645608<=scoreProtTaxo<=192.08347951924614 ^ 1.275528766157473E-
4<=scorePageRankTopoDiv<=1.3077444691159042E-4  (11)
        class DEGRADATION IF : scoreRea=0.816496580927726 ^ scoreProtTaxo=0.0 ^ 1.2621347934763986E-4<=scorePageRankTopoDiv<=1.5558559912676252E-4  (7)
        class DEGRADATION IF : 0.5773502691896257<=scoreRea<=0.6030226891555273 ^ scoreProtTaxo=0.0 ^ 1.253709265304216E-4<=scorePageRankTopoDiv<=1.4445450974778128E-4
(7)
        class BIOSYNTHESIS IF : scoreRea=0.6605782590758164 ^ scoreProtTaxo=10.60549955225613 ^ scorePageRankTopoDiv=1.163644044771755E-4  (3)
```

```
class BIOSYNTHESIS IF : 1.0<=scoreRea<=1.5491933384829668 ^ scoreProtTaxo=0.0 ^ 2.5468431845195196E-4<=scorePageRankTopoDiv<=2.5693283752350506E-4  (9)
class BIOSYNTHESIS IF : 0.8498365855987975<=scoreRea<=0.8944271909999159 ^ 0.18874604428679911<=scoreProtTaxo<=0.9389651216804074 ^ 1.6829596743622756E-4<=scorePageRankTopoDiv<=1.6920558443768528E-4  (3)
class BIOSYNTHESIS IF : 1.0377490433255416<=scoreRea<=1.1547005383792515 ^ 0.0<=scoreProtTaxo<=0.006194274740573478 ^ 1.0214790864270169E-4<=scorePageRankTopoDiv<=1.1856194866072717E-4  (22)
class DEGRADATION IF : scoreRea=0.8660254037844386 ^ scoreProtTaxo=149.76606980270952 ^ scorePageRankTopoDiv=2.018023903939492E-4  (4)
class DEGRADATION IF : 0.15836151649297917<=scoreRea<=0.49507377148833714 ^ 0.0<=scoreProtTaxo<=0.0314917783188618 ^ 3.247282870321995E-5<=scorePageRankTopoDiv<=3.507847056660574E-5  (33)
class BIOSYNTHESIS IF : 1.118033988749895<=scoreRea<=2.2771001702132443 ^ 222.4609932657902<=scoreProtTaxo<=539.0003923984657 ^ 7.809608686909931E-5<=scorePageRankTopoDiv<=1.257860095616677E-4  (26)
class BIOSYNTHESIS IF : 2.727851988394575<=scoreRea<=6.2341841409373036 ^ 41.81775198112704<=scoreProtTaxo<=426.4726970783042 ^ 2.572389005929887E-4<=scorePageRankTopoDiv<=4.229094722569427E-4  (30)
class DEGRADATION IF : 0.75<=scoreRea<=1.0 ^ 20.96265560719317<=scoreProtTaxo<=42.330049501311514 ^ 6.446530518817172E-5<=scorePageRankTopoDiv<=6.94933160911338E-5  (12)
class DEGRADATION IF : 0.6009252125773316<=scoreRea<=0.9393364366277243 ^ 0.07514655700527341<=scoreProtTaxo<=0.6377796746836621 ^ 6.672413144499774E-5<=scorePageRankTopoDiv<=6.833813604255634E-5  (18)
class DEGRADATION IF : 0.8744746321952062<=scoreRea<=0.9258200997725514 ^ scoreProtTaxo=0.0 ^ 5.557122140811065E-5<=scorePageRankTopoDiv<=6.655225016107375E-5  (8)
class BIOSYNTHESIS IF : 0.18490006540840973<=scoreRea<=0.28867513459481287 ^ 4.060796026089901<=scoreProtTaxo<=37.18965662133416 ^ 1.8669203833170315E-4<=scorePageRankTopoDiv<=2.482079768896534E-4  (14)
class BIOSYNTHESIS IF : 0.12403473458920845<=scoreRea<=0.2581988897471611 ^ 0.0<=scoreProtTaxo<=0.08306969993895892 ^ 1.258378581959181E-4<=scorePageRankTopoDiv<=1.586938187803185E-4  (22)
class OTHER IF : scoreRea=1.4142135623730951 ^ scoreProtTaxo=50.01269874088847 ^ scorePageRankTopoDiv=1.715924490479643E-4  (3)
class DEGRADATION IF : 0.816496580927726<=scoreRea<=1.0 ^ scoreProtTaxo=0.0 ^ 3.504179915896579E-4<=scorePageRankTopoDiv<=3.5357794900295656E-4  (4)
class BIOSYNTHESIS IF : scoreRea=1.0 ^ scoreProtTaxo=0.0 ^ 1.750635775572479E-4<=scorePageRankTopoDiv<=1.7605299533700306E-4  (4)
class BIOSYNTHESIS IF : scoreRea=1.4142135623730951 ^ 7.411702458499891<=scoreProtTaxo<=23.626209496421488 ^ 1.6607858601140786E-4<=scorePageRankTopoDiv<=1.7412280277644346E-4  (7)
class BIOSYNTHESIS IF : scoreRea=0.408248290463863 ^ scoreProtTaxo=0.0 ^ 7.845604803753864E-5<=scorePageRankTopoDiv<=7.922169601627488E-5  (8)
class BIOSYNTHESIS IF : 0.31622776601683794<=scoreRea<=0.7071067811865476 ^ 108.98440840078962<=scoreProtTaxo<=139.00552347161903 ^ 6.0036908760882205E-5<=scorePageRankTopoDiv<=7.51563674818164E-5  (16)
class BIOSYNTHESIS IF : scoreRea=1.0 ^ 70.03289743182262<=scoreProtTaxo<=158.40387360617618 ^ 3.480039079140226E-4<=scorePageRankTopoDiv<=3.815772077000262E-4  (15)
class BIOSYNTHESIS IF : scoreRea=1.4142135623730951 ^ 872.5390659413733<=scoreProtTaxo<=1247.2783381883148 ^ 2.2070202400956655E-4<=scorePageRankTopoDiv<=2.6174888156318006E-4  (6)
class ENERGY IF : 1.288181280536124<=scoreRea<=1.3184955758367207 ^ 12.81443337256947<=scoreProtTaxo<=19.90789234210125 ^ 2.2606890757750802E-4<=scorePageRankTopoDiv<=2.518505177794346E-4  (5)
class DEGRADATION IF : 0.125<=scoreRea<=0.18257418583505536 ^ 0.0<=scoreProtTaxo<=43.07890918143643 ^ 4.442793668447999E-5<=scorePageRankTopoDiv<=4.5095808332954006E-5  (14)
class BIOSYNTHESIS IF : scoreRea=1.0 ^ 53.43201654584469<=scoreProtTaxo<=53.61838684574613 ^ 1.8222582181223228E-4<=scorePageRankTopoDiv<=1.8377567901230646E-4  (2)
class OTHER IF : scoreRea=1.4142135623730951 ^ scoreProtTaxo=0.0 ^ scorePageRankTopoDiv=1.4179066386677404E-4  (1)
class OTHER IF : 0.8498365855987975<=scoreRea<=0.9258200997725514 ^ 0.0<=scoreProtTaxo<=0.02635667702794845 ^ 1.0144989631180699E-4<=scorePageRankTopoDiv<=1.1424636450965728E-4  (13)
class OTHER IF : 0.816496580927726<=scoreRea<=1.0 ^ 50.01269874088847<=scoreProtTaxo<=78.24924280788699 ^ 1.715924490479643E-4<=scorePageRankTopoDiv<=1.7327229527089061E-4  (4)
class OTHER IF : scoreRea=0.7171371656006362 ^ scoreProtTaxo=39.11883789500987 ^ scorePageRankTopoDiv=1.4131650372210225E-4  (2)
class OTHER IF : 0.7071067811865476<=scoreRea<=0.816496580927726 ^ scoreProtTaxo=0.0 ^ 1.2496190361871147E-4<=scorePageRankTopoDiv<=1.2604521257776215E-4  (5)
class OTHER IF : scoreRea=1.3764944032233706 ^ scoreProtTaxo=4.672811180653771 ^ scorePageRankTopoDiv=1.7450034043602316E-4  (2)
class OTHER IF : 1.3093073414159542<=scoreRea<=1.5275252316519465 ^ 33.27864608928911<=scoreProtTaxo<=54.765275877937235 ^ 1.1076165501706806E-4<=scorePageRankTopoDiv<=1.1133601723393347E-4  (10)
class OTHER IF : scoreRea=1.1881770515720091 ^ scoreProtTaxo=35.97190533881746 ^ scorePageRankTopoDiv=1.1442075717380128E-4  (1)
class OTHER IF : scoreRea=0.8660254037844386 ^ scoreProtTaxo=0.0 ^ scorePageRankTopoDiv=1.3438022261259893E-4  (1)
class OTHER IF : scoreRea=1.0 ^ scoreProtTaxo=0.0 ^ scorePageRankTopoDiv=1.5263370408995772E-4  (2)
class OTHER IF : 1.8708286933869707<=scoreRea<=2.0207259421636903 ^ 1.587883023989004<=scoreProtTaxo<=22.943877550201837 ^ 1.116493007709621E-4<=scorePageRankTopoDiv<=1.376020875302525E-4  (6)
class OTHER IF : scoreRea=1.5430334996209192 ^ scoreProtTaxo=0.0 ^ scorePageRankTopoDiv=1.1966632255606324E-4  (1)
class OTHER IF : 0.7745966692414834<=scoreRea<=1.0 ^ 1.7452983457025155<=scoreProtTaxo<=2.1645385819414065 ^ 1.506377396134267E-4<=scorePageRankTopoDiv<=1.82903042849188E-4  (11)
class OTHER IF : 1.3719886811400708<=scoreRea<=1.4752866122334647 ^ 8.009880206310035<=scoreProtTaxo<=29.305596285694808 ^ 1.0730303042438511E-4<=scorePageRankTopoDiv<=1.1945951991812146E-4  (7)
class OTHER IF : scoreRea=1.1359236684941296 ^ 0.036214922507728946<=scoreProtTaxo<=0.911596007038787 ^ 1.09640490508312E-4<=scorePageRankTopoDiv<=1.36630095633726E-4  (8)
class OTHER IF : scoreRea=1.3334899657372021 ^ scoreProtTaxo=0.786336046600834 ^ scorePageRankTopoDiv=1.5245768517501833E-4  (2)
class OTHER IF : 1.4142135623730951<=scoreRea<=1.5078740698501039 ^ 0.0<=scoreProtTaxo<=0.6787384779869023 ^ 1.2954758572089752E-4<=scorePageRankTopoDiv<=1.3162423737102732E-4  (7)
class OTHER IF : scoreRea=1.4142135623730951 ^ scoreProtTaxo=0.0 ^ scorePageRankTopoDiv=1.518647951744723E-4  (2)
class OTHER IF : 0.8660254037844386<=scoreRea<=1.3093073414159542 ^ 8.424649067330424<=scoreProtTaxo<=10.204146751821096 ^ 1.3370027787848343E-4<=scorePageRankTopoDiv<=1.885248244530818E-4  (5)
```

class DEGRADATION IF : scoreRea=1.4142135623730951 ^ scoreProtTaxo=0.0 ^ scorePageRankTopoDiv=1.48335511616314E-4  (5)
class DEGRADATION IF : scoreRea=1.4142135623730951 ^ scoreProtTaxo=0.0 ^ scorePageRankTopoDiv=1.3842061663477223E-4  (3)
class DETOX IF : scoreRea=0.25 ^ scoreProtTaxo=1.7854366557743602 ^ scorePageRankTopoDiv=1.9902855514769744E-4  (3)
class DEGRADATION IF : scoreRea=1.8516401995451028 ^ scoreProtTaxo=0.0 ^ scorePageRankTopoDiv=5.9109305162889634E-5  (3)
class DEGRADATION IF : 1.0<=scoreRea<=1.247219128924647 ^ 26.286231011957284<=scoreProtTaxo<=38.476103319852186 ^ 2.5414532404779787E-
4<=scorePageRankTopoDiv<=2.7478026043357365E-4  (11)
class DEGRADATION IF : 1.0<=scoreRea<=1.3228756555322954 ^ 0.0<=scoreProtTaxo<=44.43519300996254 ^ 4.6324579126172384E-
5<=scorePageRankTopoDiv<=4.7327178179514555E-5  (15)
class BIOSYNTHESIS IF : 0.7905694150420949<=scoreRea<=0.816496580927726 ^ 42.46502705676863<=scoreProtTaxo<=71.02648797843077 ^ 5.8031652298977115E-
5<=scorePageRankTopoDiv<=6.022372792345972E-5  (6)
class BIOSYNTHESIS IF : scoreRea=1.4142135623730951 ^ 30.982865119868915<=scoreProtTaxo<=69.47147410476717 ^ 1.9700594225831525E-
4<=scorePageRankTopoDiv<=2.4114374672442945E-4  (24)
class BIOSYNTHESIS IF : 1.0<=scoreRea<=1.035098339013531 ^ 28.753814293592814<=scoreProtTaxo<=32.55759793194711 ^ 3.4092995079697936E-
4<=scorePageRankTopoDiv<=3.739122664785864E-4  (7)
class BIOSYNTHESIS IF : scoreRea=1.4142135623730951 ^ scoreProtTaxo=1.46897744599614 ^ scorePageRankTopoDiv=7.20783349400754E-5  (6)
class BIOSYNTHESIS IF : 0.8451542547285166<=scoreRea<=1.0 ^ scoreProtTaxo=0.0 ^ 2.157200922979042E-4<=scorePageRankTopoDiv<=2.218837803860846E-4  (19)
class BIOSYNTHESIS IF : scoreRea=0.6340037731068526 ^ scoreProtTaxo=0.0 ^ scorePageRankTopoDiv=7.369165963961018E-5  (4)
class BIOSYNTHESIS IF : 0.23570226039551584<=scoreRea<=0.25 ^ 16.82062389998916<=scoreProtTaxo<=118.97441201481755 ^ 1.2244885107621335E-
4<=scorePageRankTopoDiv<=1.7973368048266475E-4  (14)
class BIOSYNTHESIS IF : 0.14213381090374028<=scoreRea<=0.2041241452319315 ^ 0.0<=scoreProtTaxo<=0.5325943121119973 ^ 4.67243619701902E-
5<=scorePageRankTopoDiv<=4.7480361500771855E-5  (12)
class BIOSYNTHESIS IF : scoreRea=0.7071067811865476 ^ scoreProtTaxo=0.0 ^ 2.2017809896165218E-4<=scorePageRankTopoDiv<=2.2430370788519718E-4  (10)
class BIOSYNTHESIS IF : 1.0<=scoreRea<=1.2792042981336627 ^ 75.06075604111761<=scoreProtTaxo<=126.37589020471343 ^ 3.69476477757441E-
5<=scorePageRankTopoDiv<=6.820390724750919E-5  (26)
class BIOSYNTHESIS IF : 0.4803844614152614<=scoreRea<=0.7385489458759964 ^ 11.049887980839541<=scoreProtTaxo<=36.19851154731422 ^ 1.9549578836361636E-
5<=scorePageRankTopoDiv<=2.395797125964908E-5  (14)
class ENERGY IF : scoreRea=1.0 ^ scoreProtTaxo=0.0 ^ scorePageRankTopoDiv=1.4166556864442256E-4  (5)
class BIOSYNTHESIS IF : scoreRea=2.057983021710106 ^ scoreProtTaxo=16.820212169959394 ^ scorePageRankTopoDiv=1.2409622007166675E-4  (3)
class BIOSYNTHESIS IF : scoreRea=1.1094003924504583 ^ scoreProtTaxo=0.0 ^ scorePageRankTopoDiv=3.773137898809049E-5  (2)
class ENERGY IF : scoreRea=0.988495919569998 ^ scoreProtTaxo=0.07850958084016749 ^ scorePageRankTopoDiv=8.177462725052139E-5  (2)
class BIOSYNTHESIS IF : 2.623303343135812<=scoreRea<=2.6568446566202857 ^ 6.485298193530774<=scoreProtTaxo<=33.036183566237966 ^ 3.176417486336401E-
4<=scorePageRankTopoDiv<=4.5982431233079004E-4  (9)
class BIOSYNTHESIS IF : 2.3326329481056884<=scoreRea<=2.743189677316311 ^ 0.0<=scoreProtTaxo<=11.502680546417647 ^ 1.2048544308923464E-
4<=scorePageRankTopoDiv<=1.9781045464983038E-4  (36)
class DEGRADATION IF : scoreRea=1.0 ^ scoreProtTaxo=0.0 ^ scorePageRankTopoDiv=5.673579070415349E-5  (5)
class BIOSYNTHESIS IF : 0.408248290463863<=scoreRea<=0.7071067811865476 ^ 0.0<=scoreProtTaxo<=0.02110942242487886 ^ 4.2470681528740396E-
5<=scorePageRankTopoDiv<=4.3178120633386797E-5  (12)
class OTHER IF : scoreRea=0.3872983346207417 ^ scoreProtTaxo=0.0 ^ scorePageRankTopoDiv=1.0030074501858312E-4  (7)
class BIOSYNTHESIS IF : 0.5707817929853929<=scoreRea<=0.6454972243679028 ^ 3.93137454201378<=scoreProtTaxo<=10.240405284120385 ^ 7.14878342041647E-
5<=scorePageRankTopoDiv<=7.631946035900328E-5  (13)
class BIOSYNTHESIS IF : 0.31622776601683794<=scoreRea<=0.5 ^ 0.0<=scoreProtTaxo<=0.11747829627593917 ^ 1.8641638185916442E-
4<=scorePageRankTopoDiv<=2.465952387977148E-4  (19)
class DEGRADATION IF : 0.408248290463863<=scoreRea<=0.4472135954999579 ^ 0.0<=scoreProtTaxo<=25.4524903361019 ^ 1.1233582394563083E-
4<=scorePageRankTopoDiv<=1.2710021931276782E-4  (7)
class BIOSYNTHESIS IF : 1.5191090506255<=scoreRea<=4.780914437337574 ^ 34.09388015135132<=scoreProtTaxo<=83.23689560348458 ^ 1.6410648387959895E-
4<=scorePageRankTopoDiv<=1.992035753298273E-4  (24)
class DEGRADATION IF : scoreRea=1.0 ^ scoreProtTaxo=0.0 ^ 2.6929902625606435E-4<=scorePageRankTopoDiv<=2.695305089690564E-4  (8)
class BIOSYNTHESIS IF : 0.21081851067789195<=scoreRea<=0.26967994498529685 ^ scoreProtTaxo=0.0 ^ 1.6946786127234814E-4<=scorePageRankTopoDiv<=1.746618148365526E-4
(5)
class ENERGY IF : 0.4387634544762784<=scoreRea<=0.7006490497453708 ^ 2.3996744093115354<=scoreProtTaxo<=4.109994975148521 ^ 1.3155681634273485E-
4<=scorePageRankTopoDiv<=1.8211138918884125E-4  (3)
class ENERGY IF : 0.19069251784911848<=scoreRea<=0.3464101615137755 ^ scoreProtTaxo=0.0 ^ 8.86812523631926E-5<=scorePageRankTopoDiv<=9.967564087338181E-5  (9)
class ENERGY IF : 0.2738612787525831<=scoreRea<=0.31622776601683794 ^ 6.471473698427908<=scoreProtTaxo<=7.995814409831503 ^ 9.568779635488795E-
5<=scorePageRankTopoDiv<=1.0824895564286868E-4  (11)
class ENERGY IF : 0.5<=scoreRea<=0.5869391856534222 ^ 59.0592922409336<=scoreProtTaxo<=78.80633749366822 ^ 1.510386644478927E-
4<=scorePageRankTopoDiv<=1.602622308140349E-4  (3)
class ENERGY IF : scoreRea=0.17541160386140583 ^ scoreProtTaxo=54.74892009226977 ^ scorePageRankTopoDiv=1.2315241317049314E-4  (1)
class ENERGY IF : 0.7071067811865476<=scoreRea<=0.7200822998230955 ^ 40.0<=scoreProtTaxo<=49.278964507472764 ^ 1.4008561574545367E-
4<=scorePageRankTopoDiv<=1.441165393286038E-4  (3)
class ENERGY IF : scoreRea=0.2672612419124244 ^ scoreProtTaxo=0.0 ^ scorePageRankTopoDiv=1.3980167757492946E-4  (2)
class ENERGY IF : 0.36313651960128146<=scoreRea<=0.37947331922020555 ^ 1.8732433424225876<=scoreProtTaxo<=9.792832691040099 ^ 8.262057952316842E-
5<=scorePageRankTopoDiv<=1.4041321871684098E-4  (11)
class DEGRADATION IF : scoreRea=2.1908902300206647 ^ scoreProtTaxo=744.0707051693515 ^ scorePageRankTopoDiv=2.956950506156661E-4  (3)
class DEGRADATION IF : 0.23570226039551584<=scoreRea<=0.3713906763541037 ^ scoreProtTaxo=0.0 ^ 9.986780345653794E-5<=scorePageRankTopoDiv<=1.053375883940584E-4
(3)
class DEGRADATION IF : scoreRea=0.3535533905932738 ^ scoreProtTaxo=6.037872455605616 ^ scorePageRankTopoDiv=1.0011139754082549E-4  (1)
class DEGRADATION IF : 1.3826657968874303<=scoreRea<=2.0 ^ 33.08715911819257<=scoreProtTaxo<=147.41252166934837 ^ 5.8394113680207267E-
5<=scorePageRankTopoDiv<=6.228331214240753E-5  (12)

class BIOSYNTHESIS IF : 0.9992257064266816<=scoreRea<=1.0709591052033318 ^ 0.007362727567874315<=scoreProtTaxo<=0.05059346213529292 ^ 9.374543842992507E-5<=scorePageRankTopoDiv<=1.2410510066503347E-4 (8)
class OTHER IF : 0.6030226891555273<=scoreRea<=0.7745966692414834 ^ 4.14886668904373<=scoreProtTaxo<=5.02223911598203 ^ 1.0751660382843966E-4<=scorePageRankTopoDiv<=1.0974209762183784E-4 (6)
class BIOSYNTHESIS IF : 0.3779644730092272<=scoreRea<=0.48507125007266594 ^ 2.5047877479323812<=scoreProtTaxo<=5.073680764092357 ^ 1.8312929137206794E-4<=scorePageRankTopoDiv<=2.433875122349829E-4 (17)
class BIOSYNTHESIS IF : scoreRea=1.525660753153479 ^ scoreProtTaxo=1.3487912944653087 ^ scorePageRankTopoDiv=1.1111324093273548E-4 (4)
class BIOSYNTHESIS IF : 0.5107539184552492<=scoreRea<=0.7071067811865476 ^ 6.516959502501294<=scoreProtTaxo<=14.399886431850499 ^ 1.2814039822525947E-4<=scorePageRankTopoDiv<=1.5623558428847628E-4 (14)
class BIOSYNTHESIS IF : 0.6009252125773316<=scoreRea<=0.6324555320336759 ^ 0.0<=scoreProtTaxo<=0.012258083904586227 ^ 1.0735501366193442E-4<=scorePageRankTopoDiv<=1.081273666574102E-4 (11)
class BIOSYNTHESIS IF : 0.14824986333222023<=scoreRea<=0.4803844614152614 ^ 0.0<=scoreProtTaxo<=25.596212983965433 ^ 1.1909069617416628E-5<=scorePageRankTopoDiv<=1.713687157423472E-5 (27)
class DEGRADATION IF : 0.21437323142813605<=scoreRea<=0.25 ^ 1.9837456301647234<=scoreProtTaxo<=13.726780403849244 ^ 3.441799469223626E-5<=scorePageRankTopoDiv<=4.6315447962956124E-5 (15)
class BIOSYNTHESIS IF : scoreRea=0.23570226039551584 ^ scoreProtTaxo=0.0 ^ 6.426013578233957E-5<=scorePageRankTopoDiv<=6.739248125225802E-5 (15)
class BIOSYNTHESIS IF : 0.5686020318963798<=scoreRea<=0.5773502691896257 ^ 0.0<=scoreProtTaxo<=0.20442280262248982 ^ 5.80121844508716E-5<=scorePageRankTopoDiv<=6.55979874717408E-5 (22)
class BIOSYNTHESIS IF : 0.5393598899705937<=scoreRea<=0.7935838209659245 ^ 0.0<=scoreProtTaxo<=0.1563812696229734 ^ 2.9144627530234042E-5<=scorePageRankTopoDiv<=3.1995246100241945E-5 (2)
class BIOSYNTHESIS IF : 0.6000000000000001<=scoreRea<=0.6324555320336759 ^ 89.25049222649379<=scoreProtTaxo<=135.17946844016356 ^ 2.8122177261014897E-5<=scorePageRankTopoDiv<=3.481353559664781E-5 (6)
class BIOSYNTHESIS IF : scoreRea=1.0 ^ scoreProtTaxo=0.0 ^ 1.019778459386172E-4<=scorePageRankTopoDiv<=1.061427001844723E-4 (10)
class OTHER IF : scoreRea=1.224744871391589 ^ scoreProtTaxo=69.47865726904281 ^ scorePageRankTopoDiv=1.9161987328237336E-4 (3)
class BIOSYNTHESIS IF : 0.3786412228313765<=scoreRea<=0.4472135954999579 ^ 0.0<=scoreProtTaxo<=32.23146137542037 ^ 6.642179634668017E-5<=scorePageRankTopoDiv<=6.979292587183943E-5 (26)
class ENERGY IF : scoreRea=0.8498365855987975 ^ scoreProtTaxo=0.037976995945837595 ^ scorePageRankTopoDiv=1.750613415112989E-4 (7)
class BIOSYNTHESIS IF : 1.1952286093343936<=scoreRea<=1.4638501094227998 ^ 28.77526436321188<=scoreProtTaxo<=40.66403414395631 ^ scorePageRankTopoDiv=7.669823584620337E-4 (6)
class BIOSYNTHESIS IF : 1.5275252316519465<=scoreRea<=2.5495097567963922 ^ 19.949670566707734<=scoreProtTaxo<=342.0920172399326 ^ 2.4781928015212094E-4<=scorePageRankTopoDiv<=2.9525891501065975E-4 (26)
class BIOSYNTHESIS IF : scoreRea=0.3370999312316211 ^ scoreProtTaxo=125.49902487565595 ^ scorePageRankTopoDiv=8.511191847200069E-5 (3)
class BIOSYNTHESIS IF : 0.4425306015783918<=scoreRea<=1.643989873053573 ^ 0.22814918814676075<=scoreProtTaxo<=0.532040781604257 ^ 4.973927196739342E-5<=scorePageRankTopoDiv<=5.106292680762229E-5 (22)
class BIOSYNTHESIS IF : scoreRea=1.0 ^ 0.0<=scoreProtTaxo<=5.949875623489494 ^ 4.588402872559206E-4<=scorePageRankTopoDiv<=5.701127800920489E-4 (29)
class DEGRADATION IF : 3.1826736817817<=scoreRea<=6.207868749305365 ^ 0.5951374046456729<=scoreProtTaxo<=3.9579657727849646 ^ 2.723813599834274E-4<=scorePageRankTopoDiv<=3.655805459617726E-4 (7)
class DEGRADATION IF : 0.48989794855663565<=scoreRea<=0.546672273590534 ^ 87.1278616789283<=scoreProtTaxo<=100.47307971677706 ^ 4.145231565697189E-5<=scorePageRankTopoDiv<=4.728125419946526E-5 (10)
class DEGRADATION IF : 0.3535533905932738<=scoreRea<=0.816496580927726 ^ scoreProtTaxo=0.0 ^ 3.6222678185925705E-5<=scorePageRankTopoDiv<=3.826109067452951E-5 (20)
class OTHER IF : scoreRea=2.086370335408023 ^ scoreProtTaxo=2.613965019897625 ^ scorePageRankTopoDiv=1.0319430370482758E-4 (2)
class BIOSYNTHESIS IF : 0.11065666703449763<=scoreRea<=0.13093073414159542 ^ 33.85577672978209<=scoreProtTaxo<=74.37073696766814 ^ 2.645619643482403E-5<=scorePageRankTopoDiv<=3.1295818290857415E-5 (11)
class DEGRADATION IF : 0.2672612419124244<=scoreRea<=0.31622776601683794 ^ 6.695548428892289<=scoreProtTaxo<=7.793743442645217 ^ 1.3196199246283668E-4<=scorePageRankTopoDiv<=1.4224012941568427E-4 (10)
class BIOSYNTHESIS IF : scoreRea=2.0 ^ scoreProtTaxo=0.0 ^ 9.350711532682093E-5<=scorePageRankTopoDiv<=1.1747951603456698E-4 (16)
class BIOSYNTHESIS IF : scoreRea=0.7905694150420949 ^ scoreProtTaxo=84.81420871526848 ^ scorePageRankTopoDiv=9.260615655659085E-5 (9)
class DEGRADATION IF : 0.9258200997725514<=scoreRea<=1.2649110640673518 ^ scoreProtTaxo=0.0 ^ 2.450964554055782E-4<=scorePageRankTopoDiv<=2.473229582541308E-4 (11)
class DEGRADATION IF : 1.0<=scoreRea<=1.7566201313073597 ^ 29.36328778093912<=scoreProtTaxo<=52.214331017798195 ^ 1.1457931062108002E-4<=scorePageRankTopoDiv<=1.1885155162372301E-4 (5)
class ENERGY IF : 2.0<=scoreRea<=2.280350850198276 ^ scoreProtTaxo=0.0 ^ 3.642818610374926E-4<=scorePageRankTopoDiv<=3.957519348612055E-4 (11)
class DEGRADATION IF : 0.816496580927726<=scoreRea<=1.0 ^ 117.68533432023266<=scoreProtTaxo<=268.8896708114055 ^ 7.025164265842389E-5<=scorePageRankTopoDiv<=7.397333394659904E-5 (7)
class BIOSYNTHESIS IF : 0.655825835783953<=scoreRea<=0.9274777915203366 ^ 0.8159397521989812<=scoreProtTaxo<=1.629651606740295 ^ 1.0610145577243671E-4<=scorePageRankTopoDiv<=1.1495607808269751E-4 (10)
class ENERGY IF : 0.08559209850218258<=scoreRea<=0.10041928905068677 ^ 0.40713606842348365<=scoreProtTaxo<=18.732065380818764 ^ 2.617285533452988E-5<=scorePageRankTopoDiv<=3.3995659231272465E-5 (8)
class DETOX IF : scoreRea=1.0 ^ scoreProtTaxo=76.23179487402706 ^ scorePageRankTopoDiv=2.61377610435628E-4 (3)
class BIOSYNTHESIS IF : scoreRea=0.75955452531275 ^ 103.81745827343019<=scoreProtTaxo<=103.8174582734302 ^ 2.0693136818277977E-4<=scorePageRankTopoDiv<=2.069313681827798E-4 (7)
class DEGRADATION IF : 0.19611613513818404<=scoreRea<=0.2721655269759087 ^ 32.32421212596552<=scoreProtTaxo<=53.712253801759694 ^ 8.048526050907137E-5<=scorePageRankTopoDiv<=8.527890121925288E-5 (7)
class BIOSYNTHESIS IF : 1.0<=scoreRea<=4.330127018922194 ^ 0.0<=scoreProtTaxo<=0.3025798391224467 ^ 7.607171232442491E-4<=scorePageRankTopoDiv<=0.0011982853264696502 (17)
class BIOSYNTHESIS IF : scoreRea=0.5773502691896257 ^ scoreProtTaxo=0.0 ^ 8.103062978938698E-5<=scorePageRankTopoDiv<=8.184055624663284E-5 (10)

```
class BIOSYNTHESIS IF : 0.4472135954999579<=scoreRea<=0.4714045207910317 ^ scoreProtTaxo=0.0 ^ 4.543426893811937E-5<=scorePageRankTopoDiv<=4.5710147296428555E-5 (16)
class DEGRADATION IF : 0.49507377148833714<=scoreRea<=1.224744871391589 ^ scoreProtTaxo=0.0 ^ 4.937666043503516E-5<=scorePageRankTopoDiv<=5.12001351746207E-5 (16)
class BIOSYNTHESIS IF : 0.2672612419124244<=scoreRea<=0.3670310333590629 ^ 0.0<=scoreProtTaxo<=0.11087798752759649 ^ 2.9726589915500942E-5<=scorePageRankTopoDiv<=3.0254070680625004E-5  (11)
class BIOSYNTHESIS IF : scoreRea=0.15781069215799975 ^ scoreProtTaxo=60.81790855857412 ^ scorePageRankTopoDiv=4.061440716194523E-5  (3)
class BIOSYNTHESIS IF : scoreRea=1.0 ^ scoreProtTaxo=370.8806689330153 ^ scorePageRankTopoDiv=2.5005781477850783E-4  (4)
class ENERGY IF : 1.0<=scoreRea<=1.247219128924647 ^ 0.0<=scoreProtTaxo<=64.47592897360235 ^ 3.945430212065747E-4<=scorePageRankTopoDiv<=3.961479461719977E-4  (3)
class BIOSYNTHESIS IF : scoreRea=1.7320508075688772 ^ scoreProtTaxo=0.0 ^ scorePageRankTopoDiv=4.02643735176089E-4  (2)
class BIOSYNTHESIS IF : 2.0<=scoreRea<=2.898275349237888 ^ 571.296896181361<=scoreProtTaxo<=668.0528309119322 ^ 2.3706178167107297E-4<=scorePageRankTopoDiv<=2.3877773348760504E-4  (6)
class DEGRADATION IF : scoreRea=1.0 ^ scoreProtTaxo=0.0 ^ scorePageRankTopoDiv=1.4244265517510647E-4  (3)
class BIOSYNTHESIS IF : 0.8660254037844386<=scoreRea<=1.0 ^ 0.0<=scoreProtTaxo<=38.00445116638788 ^ 4.3575656373539553E-4<=scorePageRankTopoDiv<=4.5685229637384094E-4  (14)
class OTHER IF : scoreRea=0.7745966692414834 ^ scoreProtTaxo=113.05081279896491 ^ scorePageRankTopoDiv=1.173629918315806E-4  (3)
class OTHER IF : scoreRea=1.0 ^ scoreProtTaxo=0.0 ^ scorePageRankTopoDiv=2.3139856423900248E-4  (4)
class DEGRADATION IF : scoreRea=0.5773502691896257 ^ scoreProtTaxo=0.0 ^ scorePageRankTopoDiv=1.802341309156001E-4  (3)
class BIOSYNTHESIS IF : 1.8049705127885605<=scoreRea<=2.2188007849009166 ^ 0.764578403234384<=scoreProtTaxo<=5.384824552790179 ^ 6.709039841234334E-5<=scorePageRankTopoDiv<=9.583471926338623E-5  (11)
class BIOSYNTHESIS IF : scoreRea=1.0 ^ scoreProtTaxo=0.0 ^ 9.402345660909281E-5<=scorePageRankTopoDiv<=9.926965555736558E-5  (15)
class BIOSYNTHESIS IF : 2.23606797749979<=scoreRea<=7.656929634557929 ^ 829.709602515744<=scoreProtTaxo<=838.1623692966704 ^ 5.044682265188456E-4<=scorePageRankTopoDiv<=6.285287540492742E-4  (5)
class OTHER IF : scoreRea=0.2672612419124244 ^ scoreProtTaxo=15.042734593454881 ^ scorePageRankTopoDiv=2.8520335842968908E-5  (4)
class BIOSYNTHESIS IF : scoreRea=0.7071067811865476 ^ scoreProtTaxo=0.0 ^ 9.998233662201861E-5<=scorePageRankTopoDiv<=1.0691434479612118E-4  (15)
class DEGRADATION IF : 0.40206235255037975<=scoreRea<=0.4472135954999579 ^ 0.0<=scoreProtTaxo<=3.6283306109744204 ^ 5.334210778502402E-5<=scorePageRankTopoDiv<=5.489064449797292E-5  (21)
class OTHER IF : scoreRea=0.4472135954999579 ^ scoreProtTaxo=2.2071721740805414 ^ scorePageRankTopoDiv=9.480868540751403E-5  (1)
class OTHER IF : scoreRea=1.0910894511799618 ^ scoreProtTaxo=0.0 ^ scorePageRankTopoDiv=9.05851900355019E-5  (1)
class OTHER IF : scoreRea=1.0 ^ scoreProtTaxo=94.50752811024493 ^ scorePageRankTopoDiv=9.350711532682093E-5  (7)
class OTHER IF : scoreRea=0.816496580927726 ^ scoreProtTaxo=0.0 ^ scorePageRankTopoDiv=1.1357442447585103E-4  (7)
class OTHER IF : scoreRea=0.3333333333333333 ^ scoreProtTaxo=173.14680927853354 ^ scorePageRankTopoDiv=9.530736098891785E-5  (3)
class OTHER IF : scoreRea=0.6666666666666666 ^ scoreProtTaxo=32.70406736719196 ^ scorePageRankTopoDiv=1.0853718564050874E-4  (3)
class OTHER IF : 0.7359800721939872<=scoreRea<=0.7698907906800384 ^ 0.01197185426793928<=scoreProtTaxo<=0.4539913281784976 ^ 8.765155464331727E-5<=scorePageRankTopoDiv<=8.802148557582406E-5  (4)
class OTHER IF : scoreRea=0.5477225575051662 ^ scoreProtTaxo=67.24186839160802 ^ scorePageRankTopoDiv=1.0356937510804866E-4  (1)
class DEGRADATION IF : scoreRea=0.34641016151377546 ^ scoreProtTaxo=1.954747307721167 ^ scorePageRankTopoDiv=2.923556711062316E-5  (7)
class BIOSYNTHESIS IF : scoreRea=0.5940885257860046 ^ scoreProtTaxo=31.050431547115572 ^ scorePageRankTopoDiv=5.9365156338514805E-5  (2)
class BIOSYNTHESIS IF : 0.408248290463863<=scoreRea<=0.5535807194106618 ^ 1.886492471379138<=scoreProtTaxo<=4.371680783476537 ^ 4.325486328638982E-5<=scorePageRankTopoDiv<=5.251282995844052E-5  (8)
class BIOSYNTHESIS IF : 0.3131121455425747<=scoreRea<=0.6755998857764941 ^ 0.0019887525736357443<=scoreProtTaxo<=0.11342319195338982 ^ 4.757809234567175E-5<=scorePageRankTopoDiv<=5.0072739563688465E-5  (14)
class DEGRADATION IF : scoreRea=0.408248290463863 ^ scoreProtTaxo=30.77857385829824 ^ scorePageRankTopoDiv=5.145063627474733E-5  (2)
class BIOSYNTHESIS IF : 1.0<=scoreRea<=1.2060453783110545 ^ 190.02809180660955<=scoreProtTaxo<=197.63897849209104 ^ 7.706175889745562E-5<=scorePageRankTopoDiv<=1.085444445793927E-4  (13)
class DEGRADATION IF : 0.4703170810017723<=scoreRea<=0.6340037731068526 ^ 0.0<=scoreProtTaxo<=21.10622305327305 ^ 6.840979034833586E-5<=scorePageRankTopoDiv<=6.990584825898163E-5  (24)
class BIOSYNTHESIS IF : 0.28867513459481287<=scoreRea<=0.31562138431599956 ^ 1.3035718214630367<=scoreProtTaxo<=19.54048218001416 ^ 6.880899824267984E-5<=scorePageRankTopoDiv<=7.486846635608594E-5  (19)
class BIOSYNTHESIS IF : scoreRea=1.0 ^ 77.49602114288327<=scoreProtTaxo<=196.6616989567411 ^ 2.31633426121055E-4<=scorePageRankTopoDiv<=2.864973939882795E-4  (33)
class BIOSYNTHESIS IF : 0.7905694150420949<=scoreRea<=0.816496580927726 ^ scoreProtTaxo=0.0 ^ 6.518594657127604E-5<=scorePageRankTopoDiv<=7.234054889925801E-5 (17)
class BIOSYNTHESIS IF : scoreRea=0.8498365855987975 ^ scoreProtTaxo=0.09349905172422704 ^ scorePageRankTopoDiv=1.985144240910256E-4  (1)
class BIOSYNTHESIS IF : scoreRea=1.0 ^ scoreProtTaxo=0.0 ^ scorePageRankTopoDiv=1.949995348430214E-4  (1)
class BIOSYNTHESIS IF : scoreRea=1.0 ^ scoreProtTaxo=0.0 ^ scorePageRankTopoDiv=1.949995348430214E-4  (1)
class BIOSYNTHESIS IF : scoreRea=1.0 ^ scoreProtTaxo=0.0 ^ scorePageRankTopoDiv=1.949995348430214E-4  (1)
class BIOSYNTHESIS IF : scoreRea=1.0 ^ scoreProtTaxo=0.0 ^ scorePageRankTopoDiv=1.949995348430214E-4  (1)
class BIOSYNTHESIS IF : scoreRea=1.0 ^ scoreProtTaxo=0.0 ^ scorePageRankTopoDiv=1.949995348430214E-4  (1)
class BIOSYNTHESIS IF : scoreRea=1.0 ^ scoreProtTaxo=0.0 ^ scorePageRankTopoDiv=1.9531762510013157E-4  (1)
class BIOSYNTHESIS IF : scoreRea=1.0 ^ scoreProtTaxo=0.0 ^ scorePageRankTopoDiv=1.9531762510013157E-4  (1)
class BIOSYNTHESIS IF : scoreRea=1.0 ^ scoreProtTaxo=0.0 ^ scorePageRankTopoDiv=1.9531762510013157E-4  (1)
class BIOSYNTHESIS IF : 0.816496580927726<=scoreRea<=1.0 ^ 47.76450951875451<=scoreProtTaxo<=50.98652966122344 ^ 2.1185263033224013E-4<=scorePageRankTopoDiv<=2.1600534257846586E-4  (8)
class BIOSYNTHESIS IF : scoreRea=1.0 ^ 36.540114942347955<=scoreProtTaxo<=82.17086870774837 ^ 1.937781347734718E-4<=scorePageRankTopoDiv<=1.9884189741881033E-4 (5)
class BIOSYNTHESIS IF : scoreRea=1.0 ^ scoreProtTaxo=0.0 ^ scorePageRankTopoDiv=2.0858327937595459E-4  (3)
class DEGRADATION IF : 0.5<=scoreRea<=0.6666666666666666 ^ 0.0<=scoreProtTaxo<=0.23909335856687908 ^ 5.14245539920205E-5<=scorePageRankTopoDiv<=5.2248192997044404E-5  (2)
```

class DEGRADATION IF : scoreRea=0.5773502691896257 ^ scoreProtTaxo=10.708252269475352 ^ scorePageRankTopoDiv=4.3863073235157044E-5  (4)
class DEGRADATION IF : scoreRea=0.5204164998665332 ^ scoreProtTaxo=0.016552321931456043 ^ scorePageRankTopoDiv=5.8244475092978616E-5  (3)
class DEGRADATION IF : 0.4815434123430768<=scoreRea<=0.5270462766947299 ^ 5.302581851067899<=scoreProtTaxo<=26.991510355854377 ^ 4.416873060053648E-5<=scorePageRankTopoDiv<=4.553895986069906E-5  (8)
class DEGRADATION IF : scoreRea=0.40206235255037975 ^ scoreProtTaxo=0.13622903541171683 ^ scorePageRankTopoDiv=4.997238646394681E-5  (2)
class DEGRADATION IF : 0.3660422490704798<=scoreRea<=0.4009791936316524 ^ scoreProtTaxo=0.0 ^ 4.527498930521919E-5<=scorePageRankTopoDiv<=5.232264527040059E-5 (9)
class DEGRADATION IF : scoreRea=0.5773502691896257 ^ scoreProtTaxo=14.091571161109593 ^ scorePageRankTopoDiv=5.12001351746207E-5  (3)
class DEGRADATION IF : scoreRea=0.49507377148833714 ^ scoreProtTaxo=15.88328747496267 ^ scorePageRankTopoDiv=4.960844882259927E-5  (2)
class DEGRADATION IF : 1.4142135623730951<=scoreRea<=1.479019945774904 ^ 111.3342879535984<=scoreProtTaxo<=166.54816786762024 ^ 7.25833440866257E-5<=scorePageRankTopoDiv<=1.0975966596067927E-4  (10)
class BIOSYNTHESIS IF : 1.5075567228888183<=scoreRea<=2.603165493823874 ^ 5.269272036115494<=scoreProtTaxo<=119.16506409178824 ^ 2.3669319923014094E-4<=scorePageRankTopoDiv<=2.437145494220669E-4  (14)
class DEGRADATION IF : 1.5811388300841898<=scoreRea<=2.6781362536479447 ^ 584.5628440521533<=scoreProtTaxo<=666.740154779605 ^ 1.157368098759784E-4<=scorePageRankTopoDiv<=1.225025970323636E-4  (10)
class BIOSYNTHESIS IF : 1.632993161855452<=scoreRea<=1.6803361008336117 ^ 20.78385916888681<=scoreProtTaxo<=44.42351344438902 ^ 1.0010568571973802E-4<=scorePageRankTopoDiv<=1.1442075717380128E-4  (6)
class DEGRADATION IF : 1.118033988749895<=scoreRea<=1.1547005383792515 ^ 0.0<=scoreProtTaxo<=14.184817258713263 ^ 6.002402324748907E-5<=scorePageRankTopoDiv<=6.562770578714856E-5  (8)
class BIOSYNTHESIS IF : 0.8451542547285166<=scoreRea<=1.0 ^ 0.30623781212204<=scoreProtTaxo<=11.104941597822085 ^ 3.200660241923604E-4<=scorePageRankTopoDiv<=3.2727400494463895E-4  (16)
class OTHER IF : scoreRea=1.1547005383792515 ^ scoreProtTaxo=155.03320327485167 ^ scorePageRankTopoDiv=5.203261468978073E-4  (4)
class ENERGY IF : scoreRea=1.5275252316519465 ^ scoreProtTaxo=78.96656333862784 ^ scorePageRankTopoDiv=4.8518016538646547E-4  (6)
class BIOSYNTHESIS IF : scoreRea=1.0 ^ scoreProtTaxo=98.47761194044129 ^ scorePageRankTopoDiv=1.957910023569396E-4  (4)
class OTHER IF : scoreRea=1.6733200530681511 ^ scoreProtTaxo=134.6667040504943 ^ scorePageRankTopoDiv=2.477764007401312E-4  (4)
class DEGRADATION IF : scoreRea=0.7071067811865476 ^ scoreProtTaxo=182.37761461514177 ^ scorePageRankTopoDiv=1.8050963412305737E-4  (5)
class BIOSYNTHESIS IF : 2.0<=scoreRea<=3.599307891947848 ^ 24.143490363208702<=scoreProtTaxo<=30.982865119868915 ^ 1.4079240203545475E-4<=scorePageRankTopoDiv<=1.837625287804995E-4  (15)
class BIOSYNTHESIS IF : scoreRea=1.0 ^ scoreProtTaxo=0.0 ^ 2.7176809938656905E-4<=scorePageRankTopoDiv<=2.78362307436891E-4  (13)
class BIOSYNTHESIS IF : 0.8660254037844386<=scoreRea<=0.875 ^ 24.173221809594498<=scoreProtTaxo<=97.10127929827577 ^ 1.8046478519252122E-4<=scorePageRankTopoDiv<=1.893923822891509E-4  (10)
class BIOSYNTHESIS IF : scoreRea=1.0 ^ scoreProtTaxo=62.08 ^ scorePageRankTopoDiv=1.7565831571757722E-4  (1)
class BIOSYNTHESIS IF : scoreRea=1.0 ^ scoreProtTaxo=62.08 ^ scorePageRankTopoDiv=1.7565831571757722E-4  (1)
class BIOSYNTHESIS IF : scoreRea=1.0 ^ scoreProtTaxo=62.08 ^ scorePageRankTopoDiv=1.7565831571757722E-4  (1)
class BIOSYNTHESIS IF : scoreRea=1.0 ^ scoreProtTaxo=62.08 ^ scorePageRankTopoDiv=1.7565831571757722E-4  (1)
class BIOSYNTHESIS IF : scoreRea=1.0 ^ scoreProtTaxo=62.08 ^ scorePageRankTopoDiv=1.7565831571757722E-4  (1)
class BIOSYNTHESIS IF : scoreRea=1.0 ^ scoreProtTaxo=62.08 ^ scorePageRankTopoDiv=1.7565831571757722E-4  (1)
class DEGRADATION IF : 1.0<=scoreRea<=1.2747548783981961 ^ 323.06434708214323<=scoreProtTaxo<=348.6018716603964 ^ 2.2611343905604424E-4<=scorePageRankTopoDiv<=2.394123836587127E-4  (12)
class ENERGY IF : scoreRea=0.5499938233462375 ^ scoreProtTaxo=0.24390229293905932 ^ scorePageRankTopoDiv=9.226288738251106E-5  (2)
class DEGRADATION IF : 1.0<=scoreRea<=2.0 ^ scoreProtTaxo=0.0 ^ 2.3317278855135453E-4<=scorePageRankTopoDiv<=2.3500344066533635E-4  (10)
class BIOSYNTHESIS IF : scoreRea=1.4142135623730951 ^ scoreProtTaxo=133.96708783565958 ^ scorePageRankTopoDiv=6.738461316833523E-5  (4)
class BIOSYNTHESIS IF : 1.4832396974191324<=scoreRea<=1.5811388300841898 ^ 59.895785832823506<=scoreProtTaxo<=95.77418042847671 ^ 7.888130487513908E-5<=scorePageRankTopoDiv<=8.290489402761552E-5  (6)
class BIOSYNTHESIS IF : 0.8498365855987975<=scoreRea<=0.9409062968745655 ^ 0.0016810934937112564<=scoreProtTaxo<=0.0687171056235235 7 ^ 6.784842619768464E-5<=scorePageRankTopoDiv<=8.338034364305354E-5  (9)
class BIOSYNTHESIS IF : scoreRea=0.8660254037844386 ^ scoreProtTaxo=0.0 ^ scorePageRankTopoDiv=4.579874297896567E-4  (5)
class BIOSYNTHESIS IF : scoreRea=0.8944271909999159 ^ scoreProtTaxo=0.0 ^ scorePageRankTopoDiv=7.360444663747284E-5  (1)
class BIOSYNTHESIS IF : scoreRea=1.0 ^ scoreProtTaxo=0.0 ^ scorePageRankTopoDiv=8.003254587501279E-5  (2)
class BIOSYNTHESIS IF : 0.7559289460184544<=scoreRea<=0.7745966692414834 ^ 12.539028237715506<=scoreProtTaxo<=44.35291965001249 ^ 7.829635104071791E-5<=scorePageRankTopoDiv<=8.138212095481503E-5  (6)
class BIOSYNTHESIS IF : scoreRea=1.0 ^ scoreProtTaxo=0.0 ^ scorePageRankTopoDiv=6.909731573460145E-5  (2)
class BIOSYNTHESIS IF : 0.5686020318963798<=scoreRea<=1.0 ^ 1.17006376814294<=scoreProtTaxo<=15.116611197217916 ^ 6.222498549850633E-5<=scorePageRankTopoDiv<=6.792037491680598E-5  (25)
class BIOSYNTHESIS IF : 0.7745966692414834<=scoreRea<=0.8401680504168059 ^ 0.0<=scoreProtTaxo<=0.02560781728536047 ^ 7.752753433104671E-5<=scorePageRankTopoDiv<=8.090769330609419E-5  (17)
class BIOSYNTHESIS IF : 0.9746794344808964<=scoreRea<=1.0677078252031313 ^ 14.589835855947749<=scoreProtTaxo<=23.80429742569377 ^ 7.06951028591058E-5<=scorePageRankTopoDiv<=8.300133176747531E-5  (12)
class BIOSYNTHESIS IF : scoreRea=0.8498365855987975 ^ scoreProtTaxo=0.177013407153926 ^ scorePageRankTopoDiv=7.63505306242672E-5  (4)
class BIOSYNTHESIS IF : 0.8660254037844386<=scoreRea<=0.9337945803757379 ^ 1.6061868465479219<=scoreProtTaxo<=4.494491622781928 ^ 7.529262716106472E-5<=scorePageRankTopoDiv<=8.297530221176349E-5  (15)
class BIOSYNTHESIS IF : 0.9128709291752768<=scoreRea<=0.9393364366277243 ^ scoreProtTaxo=0.0 ^ 6.770404143475374E-5<=scorePageRankTopoDiv<=8.093676806635258E-5 (15)
class BIOSYNTHESIS IF : scoreRea=0.8944271909999159 ^ scoreProtTaxo=0.0 ^ scorePageRankTopoDiv=7.360444663747284E-5  (1)
class BIOSYNTHESIS IF : scoreRea=0.8944271909999159 ^ scoreProtTaxo=0.0 ^ scorePageRankTopoDiv=7.360444663747284E-5  (1)
class BIOSYNTHESIS IF : scoreRea=0.8944271909999159 ^ scoreProtTaxo=0.0 ^ scorePageRankTopoDiv=7.360444663747284E-5  (1)
class BIOSYNTHESIS IF : scoreRea=0.8944271909999159 ^ scoreProtTaxo=0.0 ^ scorePageRankTopoDiv=7.360444663747284E-5  (1)
class BIOSYNTHESIS IF : scoreRea=0.8944271909999159 ^ scoreProtTaxo=0.0 ^ scorePageRankTopoDiv=7.360444663747284E-5  (1)

```
        class DEGRADATION IF : 1.632993161855452<=scoreRea<=2.091650066335189 ^ 191.5456894304651<=scoreProtTaxo<=210.79414812610932 ^ 4.456873199567818E-5<=scorePageRankTopoDiv<=1.0264834960969904E-4  (13)
        class DEGRADATION IF : scoreRea=0.816496580927726 ^ scoreProtTaxo=0.0 ^ scorePageRankTopoDiv=1.1563460165761201E-4  (8)
        class BIOSYNTHESIS IF : scoreRea=0.816496580927726 ^ scoreProtTaxo=0.0 ^ 1.0779997582493334E-4<=scorePageRankTopoDiv<=1.088867514795E-4  (8)
        class DEGRADATION IF : scoreRea=0.408248290463863 ^ 693.9891871031006<=scoreProtTaxo<=693.9891871031007 ^ scorePageRankTopoDiv=3.965053058062261E-5  (5)
        class BIOSYNTHESIS IF : scoreRea=1.3764944032233706 ^ scoreProtTaxo=0.0 ^ scorePageRankTopoDiv=1.297047059087296E-4  (3)
        class BIOSYNTHESIS IF : 2.0816659994661326<=scoreRea<=2.3717082451262845 ^ scoreProtTaxo=0.0 ^ 5.9536362836406984E-5<=scorePageRankTopoDiv<=6.25878900045181E-5 (7)
        class DEGRADATION IF : scoreRea=1.0 ^ scoreProtTaxo=0.0 ^ 1.586188952919566E-4<=scorePageRankTopoDiv<=1.6139523269247927E-4  (9)
        class DEGRADATION IF : scoreRea=0.7071067811865476 ^ scoreProtTaxo=0.0 ^ scorePageRankTopoDiv=1.600797153717709E-4  (6)
        class OTHER IF : scoreRea=0.5773502691896257 ^ scoreProtTaxo=273.0486322170335 ^ scorePageRankTopoDiv=1.231543277680165E-4  (4)
        class OTHER IF : scoreRea=0.5773502691896257 ^ scoreProtTaxo=0.0 ^ scorePageRankTopoDiv=1.563974202385334E-4  (3)
        class DEGRADATION IF : scoreRea=1.0 ^ scoreProtTaxo=0.0 ^ 1.2335250775941675E-4<=scorePageRankTopoDiv<=1.265765156839868E-4  (16)
        class DEGRADATION IF : 0.3279129178919765<=scoreRea<=0.3472581680740104 ^ 0.7273447634270425<=scoreProtTaxo<=8.65324902488168 ^ 3.5399426680855433E-5<=scorePageRankTopoDiv<=3.606821312576429E-5  (8)
        class DEGRADATION IF : 0.05455447255899809<=scoreRea<=0.2932942300427066 ^ 0.25293043138551174<=scoreProtTaxo<=0.33751706318369323 ^ 3.2713651128993574E-5<=scorePageRankTopoDiv<=3.28222925273825E-5  (6)
        class DEGRADATION IF : 0.19851666679418606<=scoreRea<=0.23186944788008415 ^ 0.6566412741523572<=scoreProtTaxo<=0.7369488592380905 ^ 2.59483006037133E-5<=scorePageRankTopoDiv<=4.8209446960439775E-5  (8)
        class BIOSYNTHESIS IF : 1.0<=scoreRea<=1.2680075462137053 ^ 4.603361687122849<=scoreProtTaxo<=41.2899157526239 ^ 1.0821870668621276E-4<=scorePageRankTopoDiv<=1.1191554303089209E-4  (15)
        class ENERGY IF : scoreRea=1.0 ^ 1.9240469741764585<=scoreProtTaxo<=3.0367993898542833 ^ 2.795286934123438E-4<=scorePageRankTopoDiv<=2.802591027677582E-4  (11)
        class BIOSYNTHESIS IF : 0.8660254037844386<=scoreRea<=1.0 ^ 497.4811236160592<=scoreProtTaxo<=602.306197130625 ^ 1.541920177321397E-4<=scorePageRankTopoDiv<=1.8564108971871575E-4  (16)
        class BIOSYNTHESIS IF : scoreRea=0.8451542547285166 ^ scoreProtTaxo=56.932828304728304 ^ scorePageRankTopoDiv=2.157200922979042E-4  (8)
        class DEGRADATION IF : scoreRea=0.5 ^ 57.502569112561936<=scoreProtTaxo<=121.94027845951078 ^ 1.128535739524047E-4<=scorePageRankTopoDiv<=1.1651066441705652E-4 (12)
        class ENERGY IF : scoreRea=1.0 ^ scoreProtTaxo=0.0 ^ scorePageRankTopoDiv=2.541628528851764E-4  (5)
        class BIOSYNTHESIS IF : 0.14907119849998599<=scoreRea<=0.19069251784911848 ^ scoreProtTaxo=0.0 ^ 1.1983187389885847E-4<=scorePageRankTopoDiv<=1.2350455368527547E-4  (17)
        class BIOSYNTHESIS IF : 1.0<=scoreRea<=1.1547005383792515 ^ 341.9994152038561<=scoreProtTaxo<=432.5671693840806 ^ 1.3935397686345004E-4<=scorePageRankTopoDiv<=2.0186555161189252E-4  (13)
        class DEGRADATION IF : scoreRea=1.0 ^ scoreProtTaxo=49.001253116811654 ^ scorePageRankTopoDiv=2.1604982948739978E-4  (4)
        class BIOSYNTHESIS IF : scoreRea=2.1821789023599236 ^ 39.831974039532746<=scoreProtTaxo<=179.69948040487637 ^ 1.2335020656697353E-4<=scorePageRankTopoDiv<=1.2529655370089888E-4  (9)
        class DEGRADATION IF : 0.3779644730092272<=scoreRea<=2.8284271247461903 ^ 177.27383616031477<=scoreProtTaxo<=803.3713373151944 ^ 4.885814936234887E-4<=scorePageRankTopoDiv<=9.091077403187453E-4  (24)
        class BIOSYNTHESIS IF : 1.6803361008336117<=scoreRea<=1.7320508075688772 ^ scoreProtTaxo=0.0 ^ 8.121377612071065E-5<=scorePageRankTopoDiv<=1.603705539214256E-4 (27)
        class BIOSYNTHESIS IF : scoreRea=0.28867513459481287 ^ scoreProtTaxo=0.0 ^ scorePageRankTopoDiv=4.585763562318558E-5  (5)
        class BIOSYNTHESIS IF : scoreRea=1.0 ^ scoreProtTaxo=121.67338448569586 ^ scorePageRankTopoDiv=3.04041959804783E-4  (1)
        class BIOSYNTHESIS IF : scoreRea=1.4142135623730951 ^ scoreProtTaxo=0.0 ^ scorePageRankTopoDiv=2.986663014466144E-4  (4)
        class BIOSYNTHESIS IF : 1.4719601443879744<=scoreRea<=2.3664319132398464 ^ 45.53429513784011<=scoreProtTaxo<=932.2968192705277 ^ 3.044045057770998E-4<=scorePageRankTopoDiv<=3.140651876434628E-4  (10)
        class BIOSYNTHESIS IF : scoreRea=0.5773502691896257 ^ 78.53198537713274<=scoreProtTaxo<=225.0807262591772 ^ 2.937946919422816E-4<=scorePageRankTopoDiv<=2.942659660207085E-4  (3)
        class BIOSYNTHESIS IF : scoreRea=1.0 ^ scoreProtTaxo=285.99815137299225 ^ scorePageRankTopoDiv=2.923036083262068E-4  (1)
        class BIOSYNTHESIS IF : 0.5773502691896257<=scoreRea<=0.7071067811865476 ^ 26.41275546024839<=scoreProtTaxo<=35.66680826927385 ^ 1.6856849441304446E-4<=scorePageRankTopoDiv<=2.937946919422816E-4  (12)
        class BIOSYNTHESIS IF : scoreRea=1.0 ^ scoreProtTaxo=0.0 ^ 2.852833775240388E-4<=scorePageRankTopoDiv<=2.928124535837693E-4  (8)
        class BIOSYNTHESIS IF : 0.5773502691896257<=scoreRea<=1.0 ^ 45.09288417624395<=scoreProtTaxo<=54.4827917683569 ^ 2.8113486581252486E-4<=scorePageRankTopoDiv<=2.932166491447716E-4  (4)
        class BIOSYNTHESIS IF : 0.816496580927726<=scoreRea<=1.0 ^ 219.6139314557716<=scoreProtTaxo<=313.8967414084758 ^ 3.0475815198214423E-4<=scorePageRankTopoDiv<=4.330581559433888E-4  (14)
        class BIOSYNTHESIS IF : scoreRea=1.0 ^ scoreProtTaxo=121.67338448569586 ^ scorePageRankTopoDiv=3.04041959804783E-4  (1)
        class BIOSYNTHESIS IF : scoreRea=1.0 ^ scoreProtTaxo=121.67338448569586 ^ scorePageRankTopoDiv=3.04041959804783E-4  (1)
        class BIOSYNTHESIS IF : scoreRea=1.0 ^ scoreProtTaxo=121.67338448569586 ^ scorePageRankTopoDiv=3.04041959804783E-4  (1)
        class BIOSYNTHESIS IF : scoreRea=1.0 ^ scoreProtTaxo=121.67338448569586 ^ scorePageRankTopoDiv=3.04041959804783E-4  (1)
        class BIOSYNTHESIS IF : scoreRea=1.0 ^ scoreProtTaxo=121.67338448569586 ^ scorePageRankTopoDiv=3.04041959804783E-4  (1)
        class BIOSYNTHESIS IF : scoreRea=1.0 ^ scoreProtTaxo=121.67338448569586 ^ scorePageRankTopoDiv=3.04041959804783E-4  (1)
        class BIOSYNTHESIS IF : scoreRea=1.0627378626481143 ^ scoreProtTaxo=14.486564680584186 ^ scorePageRankTopoDiv=9.949604816272946E-5  (2)
        class BIOSYNTHESIS IF : 1.5491933384829668<=scoreRea<=2.1821789023599236 ^ 142.8863813467606<=scoreProtTaxo<=185.9283985016919 ^ 7.862328457920213E-5<=scorePageRankTopoDiv<=9.674459453709227E-5  (12)
        class BIOSYNTHESIS IF : scoreRea=1.5811388300841898 ^ scoreProtTaxo=0.0 ^ 1.0832471275904642E-4<=scorePageRankTopoDiv<=2.1066550166109774E-4  (10)
        class BIOSYNTHESIS IF : 1.0<=scoreRea<=1.4638501094227998 ^ 12.14962939259461<=scoreProtTaxo<=108.73214139078968 ^ 1.0053122452362036E-4<=scorePageRankTopoDiv<=1.0546017914666305E-4  (21)
        class BIOSYNTHESIS IF : 0.2721655269759087<=scoreRea<=0.3333333333333333 ^ 0.05036843346547486<=scoreProtTaxo<=13.077240249309709 ^ 8.138689745012818E-5<=scorePageRankTopoDiv<=8.319487741294502E-5  (5)
```

```
        class DEGRADATION IF : scoreRea=0.7071067811865476 ^ scoreProtTaxo=682.9083793205016 ^ scorePageRankTopoDiv=2.872901893809181E-4  (3)
        class BIOSYNTHESIS IF : scoreRea=1.0 ^ scoreProtTaxo=0.0 ^ scorePageRankTopoDiv=1.3216355016187466E-4  (3)
        class BIOSYNTHESIS IF : scoreRea=1.0 ^ scoreProtTaxo=0.0 ^ scorePageRankTopoDiv=1.1483565662018686E-4  (1)
        class BIOSYNTHESIS IF : scoreRea=1.0 ^ scoreProtTaxo=0.0 ^ scorePageRankTopoDiv=1.1483565662018686E-4  (1)
        class BIOSYNTHESIS IF : scoreRea=1.0 ^ scoreProtTaxo=0.0 ^ scorePageRankTopoDiv=1.1483565662018686E-4  (1)
        class BIOSYNTHESIS IF : scoreRea=1.0 ^ scoreProtTaxo=0.0 ^ scorePageRankTopoDiv=1.1483565662018686E-4  (1)
        class BIOSYNTHESIS IF : 0.9860132971832692<=scoreRea<=1.1881770515720091 ^ 7.361536602560318<=scoreProtTaxo<=25.652671227146858 ^ 1.1411806238879287E-
4<=scorePageRankTopoDiv<=1.1821792846611631E-4  (12)
        class BIOSYNTHESIS IF : scoreRea=1.0 ^ scoreProtTaxo=0.0 ^ scorePageRankTopoDiv=1.1999325120544981E-4  (1)
        class BIOSYNTHESIS IF : scoreRea=1.0 ^ scoreProtTaxo=0.0 ^ scorePageRankTopoDiv=1.1999325120544981E-4  (1)
        class BIOSYNTHESIS IF : scoreRea=1.0 ^ scoreProtTaxo=0.0 ^ scorePageRankTopoDiv=1.1999325120544981E-4  (1)
        class BIOSYNTHESIS IF : scoreRea=0.30151134457776363 ^ scoreProtTaxo=2.507459960924724 ^ scorePageRankTopoDiv=1.315802702417396E-4  (3)
        class BIOSYNTHESIS IF : scoreRea=0.4519967646631616 ^ scoreProtTaxo=0.0 ^ scorePageRankTopoDiv=3.5221281088250244E-5  (4)
        class DEGRADATION IF : scoreRea=1.4142135623730951 ^ scoreProtTaxo=0.0 ^ scorePageRankTopoDiv=3.3482641633356434E-4  (3)
        class DEGRADATION IF : scoreRea=0.7071067811865476 ^ scoreProtTaxo=0.0 ^ scorePageRankTopoDiv=2.531695890641126E-4  (2)
        class BIOSYNTHESIS IF : scoreRea=0.6488856845230502 ^ scoreProtTaxo=0.0 ^ scorePageRankTopoDiv=7.864916424241607E-5  (2)
        class BIOSYNTHESIS IF : scoreRea=0.5865884600854132 ^ scoreProtTaxo=0.016846010778338756 ^ scorePageRankTopoDiv=9.209075396229983E-5  (6)
        class DEGRADATION IF : scoreRea=1.0 ^ scoreProtTaxo=0.0 ^ scorePageRankTopoDiv=2.609763911820804E-4  (1)
        class DEGRADATION IF : scoreRea=1.4142135623730951 ^ scoreProtTaxo=0.0 ^ scorePageRankTopoDiv=2.2111995434628557E-4  (2)
        class DEGRADATION IF : 0.816496580927726<=scoreRea<=1.0 ^ scoreProtTaxo=0.0 ^ 2.1095525286325407E-4<=scorePageRankTopoDiv<=2.156046925054501E-4  (8)
        class DEGRADATION IF : scoreRea=0.3535533905932738 ^ scoreProtTaxo=28.234455298768772 ^ scorePageRankTopoDiv=2.8703446527435645E-4  (1)
        class DEGRADATION IF : scoreRea=0.7071067811865476 ^ scoreProtTaxo=0.0 ^ 2.2527573199519746E-4<=scorePageRankTopoDiv<=2.2746501665296874E-4  (5)
        class DEGRADATION IF : scoreRea=0.8819171036881968 ^ scoreProtTaxo=49.44565641564656 ^ scorePageRankTopoDiv=2.2699784921029255E-4  (4)
        class DEGRADATION IF : scoreRea=1.632993161855452 ^ scoreProtTaxo=0.0 ^ scorePageRankTopoDiv=2.5577683916162976E-4  (1)
        class DEGRADATION IF : scoreRea=1.0 ^ scoreProtTaxo=130.2781338404075 ^ scorePageRankTopoDiv=2.89016577371395E-4  (3)
        class DEGRADATION IF : scoreRea=1.224744871391589 ^ scoreProtTaxo=0.0 ^ scorePageRankTopoDiv=2.8718895603203323E-4  (5)
        class DEGRADATION IF : 0.4472135954999579<=scoreRea<=0.5 ^ 26.098002609031003<=scoreProtTaxo<=61.54997969131992 ^ 2.0504604532525092E-
4<=scorePageRankTopoDiv<=2.9372551216232394E-4  (10)
        class DEGRADATION IF : scoreRea=1.0 ^ scoreProtTaxo=0.0 ^ scorePageRankTopoDiv=2.8232844640266373E-4  (1)
        class DEGRADATION IF : scoreRea=1.0 ^ scoreProtTaxo=0.0 ^ scorePageRankTopoDiv=2.405203763478195E-4  (3)
        class BIOSYNTHESIS IF : scoreRea=1.2909944487358056 ^ scoreProtTaxo=7.863722682721194 ^ scorePageRankTopoDiv=4.7724352816539794E-4  (3)
        class BIOSYNTHESIS IF : scoreRea=0.7071067811865476 ^ scoreProtTaxo=174.41293300255478 ^ scorePageRankTopoDiv=4.233778112332431E-4  (1)
        class BIOSYNTHESIS IF : scoreRea=1.4142135623730951 ^ scoreProtTaxo=0.0 ^ scorePageRankTopoDiv=3.7182091341019847E-4  (3)
        class BIOSYNTHESIS IF : scoreRea=1.0 ^ scoreProtTaxo=0.0 ^ scorePageRankTopoDiv=3.642818610374926E-4  (1)
        class BIOSYNTHESIS IF : scoreRea=1.0 ^ scoreProtTaxo=0.0 ^ scorePageRankTopoDiv=3.642818610374926E-4  (1)
        class DEGRADATION IF : scoreRea=0.5 ^ scoreProtTaxo=0.0 ^ scorePageRankTopoDiv=7.09543945507385E-5  (4)
        class DEGRADATION IF : 0.2773500981126146<=scoreRea<=0.29277002188455997 ^ 51.76085988447591<=scoreProtTaxo<=79.27139361808803 ^ 2.9101694011063927E-
5<=scorePageRankTopoDiv<=4.9277085691789035E-5  (7)
        class DEGRADATION IF : scoreRea=0.30403449605253013 ^ scoreProtTaxo=0.0 ^ scorePageRankTopoDiv=3.188925859954084E-5  (4)
        class DEGRADATION IF : 0.28867513459481287<=scoreRea<=0.3535533905932738 ^ scoreProtTaxo=0.0 ^ 2.8531030310799123E-5<=scorePageRankTopoDiv<=2.915896725059666E-5
(8)
        class DEGRADATION IF : 0.31154265122036146<=scoreRea<=0.3660422490704798 ^ 0.0<=scoreProtTaxo<=0.18601989901028204 ^ 3.086599717478484E-
5<=scorePageRankTopoDiv<=3.1162427507990004E-5  (5)
        class DEGRADATION IF : scoreRea=0.3535533905932738 ^ 18.295026892033253<=scoreProtTaxo<=135.57699032842908 ^ 3.549749858825105E-
5<=scorePageRankTopoDiv<=3.687615953751601E-5  (4)
        class DEGRADATION IF : scoreRea=0.4472135954999579 ^ scoreProtTaxo=0.0 ^ scorePageRankTopoDiv=3.891465433234945E-5  (3)
        class DEGRADATION IF : 0.4472135954999579<=scoreRea<=0.6030226891555273 ^ 14.888777232157643<=scoreProtTaxo<=25.93340227167837 ^ 2.719160309303253E-
5<=scorePageRankTopoDiv<=3.413336931846702E-5  (10)
        class DEGRADATION IF : 2.0<=scoreRea<=2.449489742783178 ^ scoreProtTaxo=0.0 ^ 7.75568091423706E-5<=scorePageRankTopoDiv<=9.184702952142395E-5  (4)
        class OTHER IF : 1.3333333333333333<=scoreRea<=2.449489742783178 ^ 12.23396062108038<=scoreProtTaxo<=196.6616989567411 ^ 3.1706889880715245E-
4<=scorePageRankTopoDiv<=3.2628709644987394E-4  (15)
        class BIOSYNTHESIS IF : scoreRea=0.408248290463863 ^ scoreProtTaxo=17.289366675040693 ^ scorePageRankTopoDiv=2.6039275168697428E-5  (8)
        class BIOSYNTHESIS IF : scoreRea=2.0 ^ scoreProtTaxo=404.46246448531906 ^ scorePageRankTopoDiv=4.133797272255148E-4  (1)
        class BIOSYNTHESIS IF : 2.6233033431358117<=scoreRea<=3.3189795118379384 ^ 0.018034925656415072<=scoreProtTaxo<=0.03610143342723248 ^ 2.2962848986681343E-
4<=scorePageRankTopoDiv<=4.167988354063402E-4  (6)
        class BIOSYNTHESIS IF : 1.4142135623730951<=scoreRea<=1.7320508075688772 ^ scoreProtTaxo=0.0 ^ 4.3390899382588383E-4<=scorePageRankTopoDiv<=5.594139963627898E-4
(8)
        class BIOSYNTHESIS IF : 2.4748737341529163<=scoreRea<=2.54000254000381 ^ 0.9842135491902679<=scoreProtTaxo<=83.28699216797433 ^ 3.831836775900027E-
4<=scorePageRankTopoDiv<=3.9007559818462736E-4  (2)
        class BIOSYNTHESIS IF : scoreRea=1.0 ^ scoreProtTaxo=0.0 ^ 6.301529812221773E-5<=scorePageRankTopoDiv<=6.679401603124203E-5  (5)
        class DEGRADATION IF : 0.2478788664079178<=scoreRea<=0.3637867197989336 ^ 0.10714265193236548<=scoreProtTaxo<=0.3162759272716693 ^ 6.607743171125309E-
5<=scorePageRankTopoDiv<=6.844104169344639E-5  (6)
        class BIOSYNTHESIS IF : 2.449489742783178<=scoreRea<=4.558423058385518 ^ 47.09028349250943<=scoreProtTaxo<=192.5298544265061 ^ 1.0265239631480663E-
4<=scorePageRankTopoDiv<=1.0519340961076511E-4  (6)
        class BIOSYNTHESIS IF : 0.5477225575051662<=scoreRea<=0.7071067811865476 ^ 23.04241130879896<=scoreProtTaxo<=25.763761925834835 ^ 6.524395329840937E-
5<=scorePageRankTopoDiv<=7.127803006092852E-5  (3)
        class DEGRADATION IF : 0.26111648393354675<=scoreRea<=0.262612865719445 ^ 10.200574818917099<=scoreProtTaxo<=58.56007336799859 ^ 4.156525024183641E-
5<=scorePageRankTopoDiv<=5.010702370674141E-5  (5)
```

```
        class BIOSYNTHESIS IF : scoreRea=0.3872983346207417 ^ scoreProtTaxo=14.062439847057187 ^ scorePageRankTopoDiv=3.57718714206892E-5  (13)
        class BIOSYNTHESIS IF : 0.3457459036417604<=scoreRea<=0.35805743701971643 ^ 12.185683561383481<=scoreProtTaxo<=60.85843502945625 ^ 3.949727682687551E-
5<=scorePageRankTopoDiv<=4.8160184062752934E-5  (9)
        class BIOSYNTHESIS IF : scoreRea=0.31622776601683794 ^ scoreProtTaxo=40.35275850475697 ^ scorePageRankTopoDiv=3.873442937778711E-5  (1)
        class BIOSYNTHESIS IF : scoreRea=0.1224744871391589 ^ scoreProtTaxo=20.90744024523745 ^ scorePageRankTopoDiv=3.659757676415439E-5  (5)
        class BIOSYNTHESIS IF : scoreRea=0.2169304578186516 ^ scoreProtTaxo=1.581334155432704 ^ scorePageRankTopoDiv=3.84149619368706E-5  (2)
        class BIOSYNTHESIS IF : scoreRea=0.2581988897471611 ^ scoreProtTaxo=7.811297292035066 ^ scorePageRankTopoDiv=3.7324173731263076E-5  (2)
        class BIOSYNTHESIS IF : 0.2672612419124244<=scoreRea<=0.2773500981126146 ^ 15.767142540186411<=scoreProtTaxo<=60.83421144432251 ^ 2.227982915180602E-
5<=scorePageRankTopoDiv<=2.8170111704260316E-5  (16)
        class DEGRADATION IF : scoreRea=0.2282177322938192 ^ scoreProtTaxo=0.0 ^ scorePageRankTopoDiv=3.0179017746654186E-5  (1)
        class DEGRADATION IF : 0.5773502691896257<=scoreRea<=0.6454972243679027 ^ 0.0<=scoreProtTaxo<=0.3172590498740863 ^ 7.422303094299724E-
5<=scorePageRankTopoDiv<=8.034981572344388E-5  (8)
        class BIOSYNTHESIS IF : 0.2041241452319315<=scoreRea<=0.3578810105803186 ^ 0.0<=scoreProtTaxo<=1.4727924657147426 ^ 5.965447660311724E-
5<=scorePageRankTopoDiv<=6.308082194836142E-5  (13)
        class ENERGY IF : 0.7385489458759964<=scoreRea<=0.7912565680749444 ^ 24.12140312237191<=scoreProtTaxo<=71.73402074191125 ^ 1.7392856210161315E-
4<=scorePageRankTopoDiv<=2.1357981861322832E-4  (10)
        class DEGRADATION IF : scoreRea=2.0 ^ scoreProtTaxo=8.959013470267083 ^ scorePageRankTopoDiv=2.608700894174257E-4  (3)
        class DEGRADATION IF : scoreRea=0.28867513459481287 ^ scoreProtTaxo=0.0 ^ scorePageRankTopoDiv=4.605630158757446E-5  (2)
        class BIOSYNTHESIS IF : scoreRea=0.19245008972987526 ^ scoreProtTaxo=32.10799423357924 ^ scorePageRankTopoDiv=8.527890121925288E-5  (4)
        class ENERGY IF : scoreRea=0.24253562503633297 ^ scoreProtTaxo=2.2987411082902036 ^ scorePageRankTopoDiv=2.466515140945271E-5  (2)
        class BIOSYNTHESIS IF : scoreRea=0.6009252125773316 ^ scoreProtTaxo=0.1446951787120938 ^ scorePageRankTopoDiv=1.4356825744751527E-4  (2)
        class BIOSYNTHESIS IF : 0.408248290463863<=scoreRea<=0.4472135954999579 ^ scoreProtTaxo=0.0 ^ 1.0936108451902584E-4<=scorePageRankTopoDiv<=1.1208874171937474E-4
(12)
        class OTHER IF : 1.0<=scoreRea<=1.7320508075688772 ^ scoreProtTaxo=0.0 ^ 5.844004545899021E-4<=scorePageRankTopoDiv<=5.926013986908866E-4  (7)
        class DEGRADATION IF : 3.2071349029490923<=scoreRea<=3.4641016151377544 ^ scoreProtTaxo=0.0 ^ 7.84789069021558E-5<=scorePageRankTopoDiv<=1.700829014660686E-4
(17)
        class BIOSYNTHESIS IF : 0.06537204504606135<=scoreRea<=0.10741723110591495 ^ 19.350235633395865<=scoreProtTaxo<=126.00744288078114 ^ 6.66200616101798E-
5<=scorePageRankTopoDiv<=8.067388019590874E-5  (20)
        class OTHER IF : scoreRea=1.0954451150103324 ^ scoreProtTaxo=0.0 ^ scorePageRankTopoDiv=8.47493446785784E-5  (1)
        class DEGRADATION IF : scoreRea=2.0976176963403033 ^ scoreProtTaxo=91.32623089960902 ^ scorePageRankTopoDiv=1.8669124202801856E-4  (4)
        class DEGRADATION IF : scoreRea=0.3086066999241838 ^ scoreProtTaxo=43.62489741937418 ^ scorePageRankTopoDiv=4.394931004141637E-5  (3)
        class BIOSYNTHESIS IF : scoreRea=0.408248290463863 ^ 0.1693690883496217<=scoreProtTaxo<=55.94009746764132 ^ 1.2915211751054413E-
4<=scorePageRankTopoDiv<=1.318723082960551E-4  (2)
        class BIOSYNTHESIS IF : 0.31622776601683794<=scoreRea<=0.3892494720807615 ^ 0.0<=scoreProtTaxo<=0.8046255931149046 ^ 1.256658419610188E-
4<=scorePageRankTopoDiv<=1.2611462768251616E-4  (3)
        class BIOSYNTHESIS IF : 0.14824986333222023<=scoreRea<=0.408248290463863 ^ 20.91549697888896<=scoreProtTaxo<=33.39177500970544 ^ 9.893705866072709E-
5<=scorePageRankTopoDiv<=1.0299665425326959E-4  (5)
        class DETOX IF : scoreRea=1.7320508075688772 ^ scoreProtTaxo=0.0 ^ scorePageRankTopoDiv=2.5605297194339116E-4  (3)
        class BIOSYNTHESIS IF : 1.4142135623730951<=scoreRea<=8.090398349558905 ^ 0.0<=scoreProtTaxo<=34.94361669771234 ^ 6.113644840014297E-
4<=scorePageRankTopoDiv<=6.86861693231527E-4  (11)
        class BIOSYNTHESIS IF : 0.3779644730092272<=scoreRea<=0.40206235255037975 ^ 0.0<=scoreProtTaxo<=0.6415802624320309 ^ 4.10208090166561E-
5<=scorePageRankTopoDiv<=4.4058285781172826E-5  (8)
        class OTHER IF : scoreRea=0.3429971702850177 ^ scoreProtTaxo=12.779161504403755 ^ scorePageRankTopoDiv=2.709465462200697E-5  (4)
        class BIOSYNTHESIS IF : 0.9039935293326323<=scoreRea<=0.9117917111913264 ^ 0.014986097067932382<=scoreProtTaxo<=0.12752196982754047 ^ 5.562663384351787E-
5<=scorePageRankTopoDiv<=6.2815337693300 5E-5  (7)
        class DEGRADATION IF : 2.100420126042015<=scoreRea<=2.309401076758503 ^ 0.27763102616899094<=scoreProtTaxo<=20.427731716836988 ^ 1.0491226408433257E-
4<=scorePageRankTopoDiv<=1.5280871764678444E-4  (8)
        class BIOSYNTHESIS IF : 0.5<=scoreRea<=0.7071067811865476 ^ 63.563996258345064<=scoreProtTaxo<=121.17298334143021 ^ 5.0986526676321536E-
5<=scorePageRankTopoDiv<=5.673579070415349E-5  (14)
        class OTHER IF : scoreRea=1.0 ^ scoreProtTaxo=98.47761194044129 ^ scorePageRankTopoDiv=1.8256592974131777E-4  (6)
        class BIOSYNTHESIS IF : 0.1386750490563073<=scoreRea<=0.15191090506255 ^ 178.2014346819538<=scoreProtTaxo<=295.8907046596878 ^ 1.1409009550231638E-
4<=scorePageRankTopoDiv<=1.3709365853150742E-4  (7)
        class BIOSYNTHESIS IF : scoreRea=0.7071067811865476 ^ scoreProtTaxo=0.0 ^ scorePageRankTopoDiv=1.6282912934427522E-4  (1)
        class BIOSYNTHESIS IF : scoreRea=0.4472135954999579 ^ scoreProtTaxo=41.94715730728113 ^ scorePageRankTopoDiv=1.6197739285580512E-4  (1)
        class BIOSYNTHESIS IF : scoreRea=0.6201736729460423 ^ scoreProtTaxo=0.0 ^ scorePageRankTopoDiv=1.4969734830735088E-4  (4)
        class BIOSYNTHESIS IF : 0.5773502691896257<=scoreRea<=0.7071067811865476 ^ 54.014760540597656<=scoreProtTaxo<=473.23239832180855 ^ 1.6067019311958588E-
4<=scorePageRankTopoDiv<=1.689506791098228E-4  (17)
        class BIOSYNTHESIS IF : scoreRea=0.7071067811865476 ^ scoreProtTaxo=0.0 ^ scorePageRankTopoDiv=1.6282912934427522E-4  (1)
        class BIOSYNTHESIS IF : scoreRea=0.7071067811865476 ^ scoreProtTaxo=0.0 ^ scorePageRankTopoDiv=1.6282912934427522E-4  (1)
        class BIOSYNTHESIS IF : scoreRea=0.7071067811865476 ^ scoreProtTaxo=0.0 ^ scorePageRankTopoDiv=1.6282912934427522E-4  (1)
        class DEGRADATION IF : scoreRea=0.18257418583505536 ^ scoreProtTaxo=22.268720038735957 ^ scorePageRankTopoDiv=6.369478121980365E-5  (1)
        class DEGRADATION IF : 0.30950774330130126<=scoreRea<=0.35007002100700246 ^ 0.2474538250752462<=scoreProtTaxo<=19.268303014858162 ^ 7.648579923948595E-
5<=scorePageRankTopoDiv<=7.952826632257314E-5  (7)
        class DEGRADATION IF : scoreRea=0.21320071635561044 ^ scoreProtTaxo=21.764641378281617 ^ scorePageRankTopoDiv=7.768413224144437E-5  (3)
        class DEGRADATION IF : scoreRea=0.14213381090374028 ^ scoreProtTaxo=113.04884267889307 ^ scorePageRankTopoDiv=7.340639209575947E-5  (3)
        class DEGRADATION IF : scoreRea=0.3039305703971088 ^ scoreProtTaxo=0.056819613421865284 ^ scorePageRankTopoDiv=5.170939021601188E-5  (5)
        class DEGRADATION IF : scoreRea=0.2041241452319315 ^ scoreProtTaxo=40.89029742320763 ^ scorePageRankTopoDiv=5.026181979438789E-5  (2)
```

```
        class DEGRADATION IF : 0.11605177063713189<=scoreRea<=0.19069251784911848 ^ scoreProtTaxo=0.0 ^ 7.625650949316087E-5<=scorePageRankTopoDiv<=8.56468047874572E-5
(12)
        class DEGRADATION IF : scoreRea=0.3779644730092272 ^ scoreProtTaxo=17.676317209375433 ^ scorePageRankTopoDiv=5.840067482207978E-5  (2)
        class DEGRADATION IF : 0.1690308509457033<=scoreRea<=0.2614881801842454 ^ 2.770913290327486<=scoreProtTaxo<=4.389269595667062 ^ 6.872439206549558E-
5<=scorePageRankTopoDiv<=6.900065823830583E-5  (7)
        class BIOSYNTHESIS IF : scoreRea=0.816496580927726 ^ scoreProtTaxo=29.381948174963497 ^ scorePageRankTopoDiv=1.3811067031774507E-4  (6)
        class BIOSYNTHESIS IF : 0.4731054880004788<=scoreRea<=0.655825835783953 ^ 0.2129451608611354<=scoreProtTaxo<=0.5425207638665946 ^ 4.3947989392497114E-
5<=scorePageRankTopoDiv<=4.553160486790778E-5  (9)
        class DEGRADATION IF : 1.0<=scoreRea<=1.4142135623730951 ^ scoreProtTaxo=0.0 ^ 3.779069584777629E-4<=scorePageRankTopoDiv<=3.9300224993881345E-4  (6)
        class BIOSYNTHESIS IF : scoreRea=1.949358868961793 ^ scoreProtTaxo=16.89414776770891 ^ scorePageRankTopoDiv=1.0185105332063447E-4  (3)
        class DEGRADATION IF : scoreRea=2.0 ^ scoreProtTaxo=0.0 ^ scorePageRankTopoDiv=1.5710299375717E-4  (2)
        class BIOSYNTHESIS IF : 0.7071067811865476<=scoreRea<=0.816496580927726 ^ 297.3580011933267<=scoreProtTaxo<=3913.2410008347624 ^ 6.448584985880469E-
5<=scorePageRankTopoDiv<=1.194769514392008E-4  (26)
        class DETOX IF : 0.7071067811865476<=scoreRea<=0.816496580927726 ^ scoreProtTaxo=0.0 ^ 2.990628864272763E-4<=scorePageRankTopoDiv<=3.081020677346779E-4  (5)
        class BIOSYNTHESIS IF : scoreRea=7.155417527999327 ^ scoreProtTaxo=350.7536764461839 ^ scorePageRankTopoDiv=2.2226023968397097E-4  (1)
        class BIOSYNTHESIS IF : scoreRea=3.162277660168379 ^ scoreProtTaxo=0.0 ^ scorePageRankTopoDiv=1.4574643740035997E-4  (3)
        class BIOSYNTHESIS IF : 1.8027756377319946<=scoreRea<=3.779644730092272 ^ 175.50727843332442<=scoreProtTaxo<=707.9816559175148 ^ 1.4033397296821316E-
4<=scorePageRankTopoDiv<=1.5062995730354858E-4  (12)
        class BIOSYNTHESIS IF : scoreRea=4.006187165337532 ^ scoreProtTaxo=0.5162840607074476 ^ scorePageRankTopoDiv=2.3928989987570317E-4  (2)
        class BIOSYNTHESIS IF : 4.469039979581783<=scoreRea<=4.961389383568338 5 ^ 4.06401447787824<=scoreProtTaxo<=5.9733817144801575 ^ 1.500186914875437E-
4<=scorePageRankTopoDiv<=1.9060320625320944E-4  (4)
        class BIOSYNTHESIS IF : 6.324555320336759<=scoreRea<=7.2048401940794085 ^ 0.0<=scoreProtTaxo<=0.027107567624810956 ^ 1.3831447392257697E-
4<=scorePageRankTopoDiv<=4.7412393000109237E-4  (10)
        class BIOSYNTHESIS IF : 0.5883484054145521<=scoreRea<=0.7125253031944253 ^ 32.85268093843657<=scoreProtTaxo<=54.451591212359055 ^ 1.0963920554047921E-
4<=scorePageRankTopoDiv<=1.1232116545170716E-4  (8)
        class OTHER IF : scoreRea=2.752988806446741 ^ scoreProtTaxo=2.140986903025044 ^ scorePageRankTopoDiv=2.4666729467556E-4  (5)
        class DEGRADATION IF : scoreRea=2.690370836538197 ^ scoreProtTaxo=115.30167744007765 ^ scorePageRankTopoDiv=1.21168839895759E-4  (3)
        class BIOSYNTHESIS IF : scoreRea=1.8257418583505536 ^ scoreProtTaxo=26.946669963812305 ^ scorePageRankTopoDiv=7.086179282175386E-5  (5)
        class DEGRADATION IF : 1.224744871391589<=scoreRea<=1.4142135623730951 ^ 79.17823553516814<=scoreProtTaxo<=224.318621212841 ^ 3.4578481937800454E-
4<=scorePageRankTopoDiv<=3.936306327135957E-4  (8)
        class ENERGY IF : scoreRea=0.34992710611188255 ^ scoreProtTaxo=72.64854370442542 ^ scorePageRankTopoDiv=8.914044698560096E-5  (4)
        class DEGRADATION IF : 0.03571428571428571<=scoreRea<=0.07151985398521515 ^ 2.3881652599676126<=scoreProtTaxo<=7.670614106764109 ^ 2.091755686439555E-
5<=scorePageRankTopoDiv<=4.027498609978394E-5  (14)
        class BIOSYNTHESIS IF : 0.4629100498862757<=scoreRea<=0.4803844614152614 ^ scoreProtTaxo=0.0 ^ 1.2222907282488868E-4<=scorePageRankTopoDiv<=1.2257412972154787E-4
(4)
        class BIOSYNTHESIS IF : 0.25819888974716115<=scoreRea<=0.31622776601683794 ^ 124.94102171451942<=scoreProtTaxo<=196.26152482287281 ^ 4.731607333123962E-
5<=scorePageRankTopoDiv<=5.672967459123219E-5  (19)
        class ENERGY IF : scoreRea=2.0 ^ 23.043090779329386<=scoreProtTaxo<=171.03718661796125 ^ 8.057833970960894E-4<=scorePageRankTopoDiv<=8.212633348548685E-4  (7)
        class OTHER IF : scoreRea=3.24037034920393 ^ scoreProtTaxo=16.520431758983598 ^ scorePageRankTopoDiv=1.376020875302525E-4  (5)
        class ENERGY IF : scoreRea=1.0 ^ scoreProtTaxo=0.0 ^ scorePageRankTopoDiv=2.621581168434315E-4  (2)
        class ENERGY IF : 2.449489742783178<=scoreRea<=2.8284271247461903 ^ 2.603503540460364<=scoreProtTaxo<=3.416462264904479 ^ 2.0579771942454608E-
4<=scorePageRankTopoDiv<=2.3023325596980624E-4  (7)
        class ENERGY IF : scoreRea=1.2018504251546631 ^ scoreProtTaxo=20.306853655091704 ^ scorePageRankTopoDiv=1.3345185313772253E-4  (1)
        class ENERGY IF : scoreRea=0.6837634587578277 ^ scoreProtTaxo=73.53967834571104 ^ scorePageRankTopoDiv=1.9846985407949105E-4  (1)
        class BIOSYNTHESIS IF : scoreRea=0.6454972243679028 ^ scoreProtTaxo=3.0093030929956157 ^ scorePageRankTopoDiv=8.528360635683957E-5  (2)
        class BIOSYNTHESIS IF : scoreRea=0.5 ^ scoreProtTaxo=1115.3429503058185 ^ scorePageRankTopoDiv=6.967487895208222E-5  (3)
        class DEGRADATION IF : 0.9746794344808964<=scoreRea<=1.0 ^ 7.910932501563312<=scoreProtTaxo<=62.32607531484857 ^ 5.5384972988497854E-
5<=scorePageRankTopoDiv<=5.673579070415349E-5  (3)
        class ENERGY IF : scoreRea=0.17407765595569785 ^ scoreProtTaxo=2.5899363590874156 ^ scorePageRankTopoDiv=8.642067746743294E-5  (3)
        class ENERGY IF : 0.31491832864888675<=scoreRea<=0.3636920293639972 ^ 14.540992002260255<=scoreProtTaxo<=21.347301650371904 ^ 8.53579169959795E-
5<=scorePageRankTopoDiv<=9.34097088632978E-5  (5)
        class BIOSYNTHESIS IF : scoreRea=1.4142135623730951 ^ scoreProtTaxo=25.889356934858828 ^ scorePageRankTopoDiv=1.4266988669730282E-4  (1)
        class BIOSYNTHESIS IF : 1.2909944487358056<=scoreRea<=1.311651671567906 ^ 0.0<=scoreProtTaxo<=0.6345180997481727 ^ 1.538649778103079E-
4<=scorePageRankTopoDiv<=1.5705232879669777E-4  (5)
        class BIOSYNTHESIS IF : scoreRea=1.4142135623730951 ^ scoreProtTaxo=0.0 ^ scorePageRankTopoDiv=1.3814148733742277E-4  (2)
        class BIOSYNTHESIS IF : 1.3333333333333333<=scoreRea<=1.4142135623730951 ^ 4.254489402727082<=scoreProtTaxo<=18.837414430363225 ^ 1.2775863673017025E-
4<=scorePageRankTopoDiv<=1.4036841679306012E-4  (6)
        class BIOSYNTHESIS IF : scoreRea=1.4142135623730951 ^ scoreProtTaxo=25.889356934858828 ^ scorePageRankTopoDiv=1.4266988669730282E-4  (1)
        class BIOSYNTHESIS IF : scoreRea=1.4142135623730951 ^ scoreProtTaxo=25.889356934858828 ^ scorePageRankTopoDiv=1.4266988669730282E-4  (1)
        class BIOSYNTHESIS IF : scoreRea=1.4142135623730951 ^ scoreProtTaxo=25.889356934858828 ^ scorePageRankTopoDiv=1.4266988669730282E-4  (1)
        class BIOSYNTHESIS IF : scoreRea=1.4142135623730951 ^ scoreProtTaxo=25.889356934858828 ^ scorePageRankTopoDiv=1.4266988669730282E-4  (1)
        class BIOSYNTHESIS IF : scoreRea=1.4142135623730951 ^ scoreProtTaxo=25.889356934858828 ^ scorePageRankTopoDiv=1.4266988669730282E-4  (1)
        class BIOSYNTHESIS IF : scoreRea=1.4142135623730951 ^ scoreProtTaxo=25.889356934858828 ^ scorePageRankTopoDiv=1.4266988669730282E-4  (1)
        class DEGRADATION IF : scoreRea=0.7071067811865476 ^ scoreProtTaxo=49.53075812056128 ^ scorePageRankTopoDiv=2.128797577747855E-4  (3)
        class BIOSYNTHESIS IF : 0.18257418583505536<=scoreRea<=0.454858826147342 ^ 86.68743731527776<=scoreProtTaxo<=154.10754286919666 ^ 8.635506247650476E-
6<=scorePageRankTopoDiv<=1.722019170849132E-5  (6)
        class DEGRADATION IF : 0.724568837309472<=scoreRea<=0.7385489458759964 ^ 15.845995698294628<=scoreProtTaxo<=33.91166461882087 ^ 3.330277643310726E-
5<=scorePageRankTopoDiv<=4.1805930719753834E-5  (10)
```

class BIOSYNTHESIS IF : 0.5400617248673216<=scoreRea<=0.6030226891555273 ^ scoreProtTaxo=0.0 ^ 3.856354313589377E-5<=scorePageRankTopoDiv<=3.972820985848399E-5 (10)
class OTHER IF : scoreRea=0.9428090415820634 ^ scoreProtTaxo=66.20588492072285 ^ scorePageRankTopoDiv=2.242015684498887E-4 (3)
class DEGRADATION IF : scoreRea=2.6457513110645907 ^ scoreProtTaxo=241.16138616799333 ^ scorePageRankTopoDiv=3.360824014842919E-4 (2)
class DEGRADATION IF : 2.0<=scoreRea<=3.2498506536772873 ^ 0.0<=scoreProtTaxo<=0.0024079724851000252 ^ 2.542965180805312E-4<=scorePageRankTopoDiv<=2.95211782315212E-4 (9)
class DEGRADATION IF : scoreRea=1.4142135623730951 ^ scoreProtTaxo=0.0 ^ scorePageRankTopoDiv=3.0243191704417294E-4 (1)
class DEGRADATION IF : 0.5773502691896257<=scoreRea<=0.8660254037844386 ^ 44.831569115369774<=scoreProtTaxo<=77.27660504356028 ^ 3.1345088576072296E-4<=scorePageRankTopoDiv<=3.6066606573853435E-4 (10)
class DEGRADATION IF : scoreRea=1.4142135623730951 ^ scoreProtTaxo=160.94856643572083 ^ scorePageRankTopoDiv=3.1005745950147753E-4 (2)
class DEGRADATION IF : scoreRea=1.0 ^ 32.46883637689602<=scoreProtTaxo<=71.39072873809421 ^ 3.140914019212182E-4<=scorePageRankTopoDiv<=3.268750300999243E-4 (7)
class DEGRADATION IF : 1.4142135623730951<=scoreRea<=2.1602468994692865 ^ 21.13898069316951<=scoreProtTaxo<=74.56938331200074 ^ 3.58337921459429E-4<=scorePageRankTopoDiv<=3.8436270956853036E-4 (10)
class DEGRADATION IF : scoreRea=1.4142135623730951 ^ scoreProtTaxo=0.0 ^ scorePageRankTopoDiv=3.2941395309936893E-4 (3)
class DEGRADATION IF : scoreRea=0.8084520834544432 ^ scoreProtTaxo=16.486688450760568 ^ scorePageRankTopoDiv=4.504717622040652E-5 (2)
class DEGRADATION IF : scoreRea=0.8744746321952062 ^ scoreProtTaxo=62.371656238981856 ^ scorePageRankTopoDiv=5.077560580601176E-5 (4)
class DEGRADATION IF : scoreRea=0.5865884600854132 ^ scoreProtTaxo=0.0 ^ scorePageRankTopoDiv=6.626181790340842E-5 (3)
class DEGRADATION IF : scoreRea=0.655825835783953 ^ scoreProtTaxo=0.12645644717105106 ^ scorePageRankTopoDiv=4.7873986812249354E-5 (1)
class DEGRADATION IF : scoreRea=0.7071067811865476 ^ scoreProtTaxo=29.260568309485883 ^ scorePageRankTopoDiv=6.49456574064399E-5 (4)
class DEGRADATION IF : 0.6123724356957945<=scoreRea<=0.7958224257542215 ^ 0.0<=scoreProtTaxo<=3.8486696703969288 ^ 5.860706774825163E-5<=scorePageRankTopoDiv<=5.8803733051915237E-5 (5)
class DEGRADATION IF : 0.7071067811865476<=scoreRea<=0.8401680504168059 ^ 15.13088285984221<=scoreProtTaxo<=22.94891351632441 ^ 5.673579070415349E-5<=scorePageRankTopoDiv<=5.8739070632628016E-5 (6)
class DEGRADATION IF : scoreRea=0.7071067811865476 ^ scoreProtTaxo=0.0 ^ scorePageRankTopoDiv=5.7345542270816386E-5 (5)
class BIOSYNTHESIS IF : 2.059867849051379<=scoreRea<=2.11332577548596 ^ 0.32552899071239816<=scoreProtTaxo<=1.3575694107361542 ^ 1.8287761797669406E-4<=scorePageRankTopoDiv<=2.2495306725919904E-4 (9)
class BIOSYNTHESIS IF : 2.119864892037657<=scoreRea<=2.140872096444188 ^ 3.2379181783169733<=scoreProtTaxo<=3.6899110841277327 ^ 2.660174404203223E-4<=scorePageRankTopoDiv<=2.985873638512974E-4 (8)
class BIOSYNTHESIS IF : scoreRea=0.7071067811865476 ^ scoreProtTaxo=57.19877992820294 ^ scorePageRankTopoDiv=1.273031544422776E-4 (7)
class BIOSYNTHESIS IF : scoreRea=0.6642111641550714 ^ scoreProtTaxo=4.442629524810974 ^ scorePageRankTopoDiv=9.992258732964255E-5 (2)
class BIOSYNTHESIS IF : scoreRea=0.6546536707079771 ^ scoreProtTaxo=0.0 ^ scorePageRankTopoDiv=9.507727063923345E-5 (1)
class BIOSYNTHESIS IF : 0.18346822357102757<=scoreRea<=0.2672612419124244 ^ 1.0041442365714153<=scoreProtTaxo<=4.894951118682685 ^ 7.206541728125592E-5<=scorePageRankTopoDiv<=9.91658068652667E-5 (9)
class BIOSYNTHESIS IF : 0.5686020318963798<=scoreRea<=0.5773502691896257 ^ scoreProtTaxo=0.0 ^ 9.680375868053309E-5<=scorePageRankTopoDiv<=1.1386306057343698E-4 (8)
class BIOSYNTHESIS IF : 0.09245003270420486<=scoreRea<=0.4472135954999579 ^ 41.11374693353282<=scoreProtTaxo<=90.72358809850219 ^ 9.68361607384624E-5<=scorePageRankTopoDiv<=9.71025528247856E-5 (5)
class DEGRADATION IF : scoreRea=1.5 ^ scoreProtTaxo=328.6761930573438 ^ scorePageRankTopoDiv=1.9482402096573516E-4 (3)
class DEGRADATION IF : 2.449489742783178<=scoreRea<=3.4641016151377544 ^ 186.8047092621161<=scoreProtTaxo<=526.7533706709773 ^ 2.1608188604580465E-4<=scorePageRankTopoDiv<=2.337782760475997E-4 (9)
class BIOSYNTHESIS IF : scoreRea=1.202652838880877 ^ scoreProtTaxo=1.932961341016369 ^ scorePageRankTopoDiv=1.0051476168913065E-4 (4)
class OTHER IF : 0.408248290463863<=scoreRea<=0.5 ^ 355.1112760084071<=scoreProtTaxo<=521.8331253857299 ^ 5.4018423913444024E-5<=scorePageRankTopoDiv<=6.384999288989806E-5 (7)
class BIOSYNTHESIS IF : 0.19839002137983244<=scoreRea<=0.24397501823713327 ^ 1.2825136528866317<=scoreProtTaxo<=34.62065082470671 ^ 4.958838726520721E-5<=scorePageRankTopoDiv<=5.205683608903145E-5 (5)
class BIOSYNTHESIS IF : 0.28867513459481287<=scoreRea<=0.408248290463863 ^ 89.0085484841533<=scoreProtTaxo<=98.81836756108136 ^ 1.1510213213064633E-4<=scorePageRankTopoDiv<=1.185665663845858E-4 (15)
class BIOSYNTHESIS IF : 1.0<=scoreRea<=1.8027756377319946 ^ 199.87966851217695<=scoreProtTaxo<=205.33627510231165 ^ 1.1807420237890181E-4<=scorePageRankTopoDiv<=1.1924321543192456E-4 (7)
class BIOSYNTHESIS IF : scoreRea=0.3086066999241838 ^ scoreProtTaxo=0.0 ^ scorePageRankTopoDiv=4.303619168485682E-5 (1)
class BIOSYNTHESIS IF : scoreRea=0.9746794344808964 ^ scoreProtTaxo=14.537153264002997 ^ scorePageRankTopoDiv=5.4546243901817575E-5 (7)
class OTHER IF : 0.1336306209562122<=scoreRea<=0.2041241452319315 ^ 11.48907832227296<=scoreProtTaxo<=24.204940852237677 ^ 2.277997394999028E-5<=scorePageRankTopoDiv<=2.2992049064083972E-5 (8)
class DEGRADATION IF : 1.0933445471810679<=scoreRea<=1.2126781251816647 ^ scoreProtTaxo=0.0 ^ 1.220459139739041E-4<=scorePageRankTopoDiv<=1.2410399332421477E-4 (5)
class DEGRADATION IF : scoreRea=1.3258750156531338 ^ scoreProtTaxo=0.7079245963934496 ^ scorePageRankTopoDiv=1.295463900452369E-4 (3)
class DEGRADATION IF : 2.1143765594836976<=scoreRea<=2.211398230003235 ^ 33.436888932748474<=scoreProtTaxo<=157.89757001622166 ^ 1.4383666539306183E-4<=scorePageRankTopoDiv<=1.5764329330262091E-4 (4)
class DEGRADATION IF : scoreRea=1.8973665961010275 ^ scoreProtTaxo=0.0 ^ scorePageRankTopoDiv=1.068428112262252E-4 (2)
class DEGRADATION IF : 1.5491933384829668<=scoreRea<=2.0 ^ 24.540654200910076<=scoreProtTaxo<=247.55660370974545 ^ 1.37032589402138E-4<=scorePageRankTopoDiv<=1.3902458959959068E-4 (8)
class DEGRADATION IF : scoreRea=1.224744871391589 ^ scoreProtTaxo=258.6785545214659 ^ scorePageRankTopoDiv=3.191856776052939E-5 (12)
class BIOSYNTHESIS IF : scoreRea=0.8473185457363234 ^ scoreProtTaxo=2.896420549071816 ^ scorePageRankTopoDiv=9.581353925799543E-5 (2)
class DEGRADATION IF : 0.816496580927726<=scoreRea<=1.0 ^ 138.45986359634793<=scoreProtTaxo<=168.12250435495292 ^ 9.184702952142395E-5<=scorePageRankTopoDiv<=1.0609466112632037E-4 (3)
class DEGRADATION IF : scoreRea=0.816496580927726 ^ scoreProtTaxo=128.28625663138507 ^ scorePageRankTopoDiv=1.0926502894973586E-4 (5)
class DEGRADATION IF : scoreRea=0.356034497458156 ^ scoreProtTaxo=0.0 ^ scorePageRankTopoDiv=7.818754435989404E-5 (2)
class DETOX IF : scoreRea=1.4142135623730951 ^ scoreProtTaxo=0.0 ^ scorePageRankTopoDiv=2.1605297494252306E-4 (2)

```
        class BIOSYNTHESIS IF : scoreRea=0.4472135954999579 ^ scoreProtTaxo=41.94715730728113 ^ scorePageRankTopoDiv=1.6197739285580512E-4  (1)
        class BIOSYNTHESIS IF : scoreRea=0.4472135954999579 ^ scoreProtTaxo=41.94715730728113 ^ scorePageRankTopoDiv=1.6197739285580512E-4  (1)
        class BIOSYNTHESIS IF : scoreRea=0.4472135954999579 ^ scoreProtTaxo=41.94715730728113 ^ scorePageRankTopoDiv=1.6197739285580512E-4  (1)
        class BIOSYNTHESIS IF : scoreRea=0.8660254037844386 ^ scoreProtTaxo=0.0 ^ scorePageRankTopoDiv=9.834448953690958E-5  (3)
        class BIOSYNTHESIS IF : scoreRea=1.2909944487358056 ^ scoreProtTaxo=0.6405682128609419 ^ scorePageRankTopoDiv=2.0320445389278625E-4  (3)
        class BIOSYNTHESIS IF : scoreRea=0.5 ^ scoreProtTaxo=25.84565026836747 ^ scorePageRankTopoDiv=4.95954293050021E-5  (4)
        class DEGRADATION IF : scoreRea=0.11180339887498948 ^ scoreProtTaxo=53.33607029865077 ^ scorePageRankTopoDiv=2.221322151018711E-5  (5)
        class DEGRADATION IF : scoreRea=0.33071891388307384 ^ scoreProtTaxo=0.6368952509453817 ^ scorePageRankTopoDiv=2.5582106535976228E-5  (3)
        class OTHER IF : scoreRea=2.035529993132299 ^ scoreProtTaxo=37.18309334171484 ^ scorePageRankTopoDiv=1.6214399066947588E-4  (6)
        class ENERGY IF : 0.22360679774997896<=scoreRea<=0.439586982263858 ^ 0.03824909535505272<=scoreProtTaxo<=0.25395543377941004 ^ 3.4356952197702415E-
5<=scorePageRankTopoDiv<=3.5129610103536256E-5  (4)
        class BIOSYNTHESIS IF : 1.7320508075688772<=scoreRea<=1.9364916731037085 ^ 21.379806720813527<=scoreProtTaxo<=139.060820931979 ^ 1.4466068132218887E-
4<=scorePageRankTopoDiv<=1.492140440385095E-4  (7)
        class BIOSYNTHESIS IF : scoreRea=0.3872983346207417 ^ scoreProtTaxo=0.0 ^ scorePageRankTopoDiv=5.3414666428876934E-5  (3)
        class BIOSYNTHESIS IF : scoreRea=1.0 ^ scoreProtTaxo=98.40420334876411 ^ scorePageRankTopoDiv=1.7230729652463356E-4  (2)
        class OTHER IF : scoreRea=2.6457513110645907 ^ scoreProtTaxo=11.653831840400214 ^ scorePageRankTopoDiv=1.2548442637641923E-4  (4)
        class DEGRADATION IF : scoreRea=0.7071067811865476 ^ scoreProtTaxo=206.55452266500356 ^ scorePageRankTopoDiv=1.1490929097897867E-4  (2)
        class BIOSYNTHESIS IF : 1.224744871391589<=scoreRea<=1.4142135623730951 ^ 51.50365682807401<=scoreProtTaxo<=105.77882039783354 ^ 2.5761354379839283E-
4<=scorePageRankTopoDiv<=2.7358989262553504E-4  (5)
        class BIOSYNTHESIS IF : scoreRea=1.7638342073763935 ^ scoreProtTaxo=79.0514992333247 ^ scorePageRankTopoDiv=1.3677116599979192E-4  (3)
        class BIOSYNTHESIS IF : scoreRea=1.949358868961793 ^ scoreProtTaxo=34.87119154833509 ^ scorePageRankTopoDiv=1.359130639592478E-4  (4)
        class BIOSYNTHESIS IF : scoreRea=0.08333333333333333 ^ scoreProtTaxo=71.90009062732221 ^ scorePageRankTopoDiv=3.470487807565085E-5  (3)
        class DEGRADATION IF : scoreRea=0.7071067811865476 ^ 109.4646547441358<=scoreProtTaxo<=146.491662142068 ^ 4.371498400030517E-
5<=scorePageRankTopoDiv<=5.058178454993116E-5  (6)
        class DEGRADATION IF : scoreRea=0.23570226039551584 ^ scoreProtTaxo=0.2303427360363902 ^ scorePageRankTopoDiv=1.448780413994269E-4  (2)
        class DETOX IF : scoreRea=1.0 ^ scoreProtTaxo=608.3681488898235 ^ scorePageRankTopoDiv=3.396371341897463E-4  (1)
        class DETOX IF : scoreRea=2.581988897471611 ^ scoreProtTaxo=27.930778633284643 ^ scorePageRankTopoDiv=4.568212402645179E-4  (3)
        class DETOX IF : scoreRea=1.2909944487358056 ^ scoreProtTaxo=0.0 ^ scorePageRankTopoDiv=4.810911994972087E-4  (3)
        class DETOX IF : scoreRea=1.0 ^ scoreProtTaxo=0.0 ^ scorePageRankTopoDiv=2.5005781477850783E-4  (3)
        class DETOX IF : scoreRea=1.4142135623730951 ^ scoreProtTaxo=146.53881007132415 ^ scorePageRankTopoDiv=2.6652816827249016E-4  (2)
        class DETOX IF : scoreRea=1.4142135623730951 ^ scoreProtTaxo=0.0 ^ scorePageRankTopoDiv=3.3284914715507266E-4  (1)
        class DETOX IF : scoreRea=1.0 ^ scoreProtTaxo=290.5100020231181 ^ 2.626806104123039E-4<=scorePageRankTopoDiv<=3.0321704900335665E-4  (5)
        class BIOSYNTHESIS IF : scoreRea=1.0 ^ scoreProtTaxo=0.0 ^ 2.2507399536377622E-4<=scorePageRankTopoDiv<=2.2734171088002557E-4  (8)
        class BIOSYNTHESIS IF : scoreRea=1.7320508075688772 ^ scoreProtTaxo=113.37384803240558 ^ scorePageRankTopoDiv=9.025333891358375E-5  (3)
        class DEGRADATION IF : 0.9013878188659973<=scoreRea<=1.093344547181068 ^ 200.94615943355413<=scoreProtTaxo<=424.7421113040829 ^ 8.070273683221971E-
5<=scorePageRankTopoDiv<=8.622735221921629E-5  (9)
        class BIOSYNTHESIS IF : 0.8660254037844386<=scoreRea<=1.0 ^ 308.8787482384362<=scoreProtTaxo<=321.94884504151656 ^ 1.237945613915102E-
4<=scorePageRankTopoDiv<=1.2392035308247696E-4  (3)
        class DEGRADATION IF : 0.21081851067789195<=scoreRea<=0.6324555320336759 ^ 6.648788023034921<=scoreProtTaxo<=12.431118389046912 ^ 1.8381833697315455E-
4<=scorePageRankTopoDiv<=1.8416305184943934E-4  (7)
        class ENERGY IF : scoreRea=0.7071067811865476 ^ scoreProtTaxo=2.883461995813866 ^ scorePageRankTopoDiv=3.288585667948916E-4  (2)
        class ENERGY IF : scoreRea=0.5898297717679785 ^ scoreProtTaxo=80.88172121496505 ^ scorePageRankTopoDiv=7.984042811155853E-5  (1)
        class BIOSYNTHESIS IF : scoreRea=0.9486832980505138 ^ scoreProtTaxo=492.4769364822055 ^ scorePageRankTopoDiv=6.382584844988021E-5  (4)
        class ENERGY IF : scoreRea=0.16035674514745463 ^ scoreProtTaxo=44.11999794819986 ^ scorePageRankTopoDiv=4.305270368731032E-5  (3)
        class BIOSYNTHESIS IF : scoreRea=1.4142135623730951 ^ scoreProtTaxo=212.39271801691976 ^ scorePageRankTopoDiv=1.306755114111501E-4  (4)
        class DEGRADATION IF : scoreRea=1.1547005383792515 ^ scoreProtTaxo=120.27240963691459 ^ scorePageRankTopoDiv=9.346737750457407E-5  (2)
        class DEGRADATION IF : scoreRea=1.0 ^ scoreProtTaxo=0.0 ^ scorePageRankTopoDiv=2.3162289563086192E-5  (2)
        class ENERGY IF : scoreRea=1.1710800875382399 ^ scoreProtTaxo=83.83084120930127 ^ scorePageRankTopoDiv=3.405667421549469E-4  (4)
        class DEGRADATION IF : 3.7951582057210227<=scoreRea<=4.307507614472608 ^ 0.3018617796253848<=scoreProtTaxo<=1.379921366421163 ^ 1.7404809878674647E-
4<=scorePageRankTopoDiv<=1.9574314093248436E-4  (6)
        class BIOSYNTHESIS IF : scoreRea=1.4142135623730951 ^ scoreProtTaxo=0.0 ^ 2.0875414097402804E-4<=scorePageRankTopoDiv<=2.1395082657941635E-4  (6)
        class OTHER IF : 7.082843120291925<=scoreRea<=8.031189202104505 ^ 4.863076241064854<=scoreProtTaxo<=12.209490805052312 ^ 1.0283029333560688E-
4<=scorePageRankTopoDiv<=1.0442531089430809E-4  (6)
        class DEGRADATION IF : scoreRea=1.0 ^ scoreProtTaxo=24.522053185615338 ^ scorePageRankTopoDiv=7.175420992019046E-5  (5)
        class BIOSYNTHESIS IF : scoreRea=0.7071067811865476 ^ scoreProtTaxo=6.738311679550256 ^ scorePageRankTopoDiv=5.673579070415349E-5  (4)
        class DEGRADATION IF : scoreRea=1.4142135623730951 ^ scoreProtTaxo=6.461124413009387 ^ scorePageRankTopoDiv=7.304909884610215E-4  (3)
        class BIOSYNTHESIS IF : scoreRea=1.224744871391589 ^ scoreProtTaxo=150.04371593384334 ^ scorePageRankTopoDiv=7.267741345896467E-5  (4)
        class DEGRADATION IF : scoreRea=1.0 ^ 176.6748265891641<=scoreProtTaxo<=265.45642259993303 ^ 1.2292863073691518E-4<=scorePageRankTopoDiv<=1.318125744054719E-4
(7)
        class BIOSYNTHESIS IF : 0.6258327785172862<=scoreRea<=0.696630546019236 ^ 0.3172590498740864<=scoreProtTaxo<=1.0730405606481515 ^ 7.221388362580414E-
5<=scorePageRankTopoDiv<=7.693248890515395E-5  (7)
        class DEGRADATION IF : scoreRea=0.6324555320336759 ^ scoreProtTaxo=0.0 ^ scorePageRankTopoDiv=7.186184244774277E-5  (4)
        class BIOSYNTHESIS IF : 0.7071067811865476<=scoreRea<=0.816496580927726 ^ 122.41731903615599<=scoreProtTaxo<=132.7672987437464 ^ 8.461694462624069E-
5<=scorePageRankTopoDiv<=8.9145592096062E-5  (3)
        class BIOSYNTHESIS IF : scoreRea=0.5 ^ scoreProtTaxo=59.600361397029246 ^ scorePageRankTopoDiv=6.177647995694302E-5  (3)
        class DEGRADATION IF : 0.5252257314388902<=scoreRea<=1.2909944487358056 ^ 184.36354556058535<=scoreProtTaxo<=184.90955657450604 ^ 7.521950513745555E-
5<=scorePageRankTopoDiv<=8.499128390523789E-5  (7)
        class OTHER IF : scoreRea=1.0 ^ scoreProtTaxo=0.0 ^ scorePageRankTopoDiv=2.075060281598621E-4  (2)
```

```
        class BIOSYNTHESIS IF : scoreRea=0.5 ^ scoreProtTaxo=0.0 ^ scorePageRankTopoDiv=7.06019595021213E-5  (1)
        class DEGRADATION IF : scoreRea=0.4714045207910317 ^ scoreProtTaxo=0.21736656470342558 ^ scorePageRankTopoDiv=2.039267862920187E-4  (3)
        class DEGRADATION IF : scoreRea=0.5773502691896257 ^ scoreProtTaxo=0.0 ^ scorePageRankTopoDiv=2.1673517381795885E-4  (3)
        class BIOSYNTHESIS IF : scoreRea=0.7071067811865476 ^ 37.351611171971676<=scoreProtTaxo<=46.43188130854518 ^ 2.1106370026593825E-
4<=scorePageRankTopoDiv<=2.8099010423062484E-4  (4)
        class DEGRADATION IF : scoreRea=4.242640687119285 ^ scoreProtTaxo=0.0 ^ scorePageRankTopoDiv=5.351422632147156E-4  (2)
        class OTHER IF : scoreRea=1.0 ^ scoreProtTaxo=278.9260654725549 ^ scorePageRankTopoDiv=1.5119971642290792E-4  (2)
        class DEGRADATION IF : scoreRea=1.0 ^ scoreProtTaxo=162.15630319750082 ^ scorePageRankTopoDiv=4.63834105930411E-4  (6)
        class DEGRADATION IF : scoreRea=5.392738882336249 ^ scoreProtTaxo=31.56070563126379 ^ scorePageRankTopoDiv=2.6560409197769793E-4  (2)
        class ENERGY IF : scoreRea=0.3469443332443555 ^ scoreProtTaxo=0.008501686508290446 ^ scorePageRankTopoDiv=4.25938615935737E-5  (3)
        class BIOSYNTHESIS IF : scoreRea=0.5773502691896257 ^ scoreProtTaxo=0.0 ^ scorePageRankTopoDiv=1.8401674405587953E-4  (2)
        class BIOSYNTHESIS IF : scoreRea=0.816496580927726 ^ scoreProtTaxo=53.96055802488666 ^ scorePageRankTopoDiv=1.799068798674255E-4  (3)
        class DEGRADATION IF : 6.76930789754497<=scoreRea<=17.580173491749164 ^ 0.25453539884412596<=scoreProtTaxo<=48.876164669699776 ^ 2.27214944912841E-
4<=scorePageRankTopoDiv<=5.500256568628485E-4  (3)
        class DEGRADATION IF : scoreRea=0.3779644730092272 ^ scoreProtTaxo=27.294314276743904 ^ scorePageRankTopoDiv=4.608224295654832E-5  (3)
        class ENERGY IF : scoreRea=1.0 ^ scoreProtTaxo=0.0 ^ scorePageRankTopoDiv=2.9556844973405226E-4  (3)
        class BIOSYNTHESIS IF : 0.816496580927726<=scoreRea<=0.8660254037844386 ^ 65.79623872982258<=scoreProtTaxo<=130.03392307060201 ^ 4.77018583093523E-
5<=scorePageRankTopoDiv<=5.0451195012364305E-5  (3)
        class DEGRADATION IF : scoreRea=2.8284271247461903 ^ scoreProtTaxo=46.08171497882105 ^ scorePageRankTopoDiv=9.184702952142395E-5  (1)
        class OTHER IF : scoreRea=0.39528470752104744 ^ scoreProtTaxo=67.31782581752879 ^ scorePageRankTopoDiv=3.0197216976965264E-5  (3)
        class BIOSYNTHESIS IF : 0.5773502691896257<=scoreRea<=0.7071067811865476 ^ 0.0<=scoreProtTaxo<=0.22537446792761007 ^ 2.3274077720938588E-
4<=scorePageRankTopoDiv<=2.423052815653982E-4  (9)
        class BIOSYNTHESIS IF : 0.816496580927726<=scoreRea<=1.0 ^ 32.13477923659716<=scoreProtTaxo<=286.698115661492 ^ 2.16674851498363E-
4<=scorePageRankTopoDiv<=2.209479886732525E-4  (12)
        class BIOSYNTHESIS IF : scoreRea=1.0 ^ 15.54358844509754<=scoreProtTaxo<=20.97192084929702 ^ 4.6220909847007676E-4<=scorePageRankTopoDiv<=5.428246164357167E-4
(5)
        class BIOSYNTHESIS IF : 0.8451542547285166<=scoreRea<=0.9428090415820634 ^ 39.943773190847274<=scoreProtTaxo<=217.84101623961143 ^ 2.3351532071486775E-
4<=scorePageRankTopoDiv<=2.500264397421409E-4  (11)
        class BIOSYNTHESIS IF : scoreRea=1.632993161855452 ^ scoreProtTaxo=0.0 ^ scorePageRankTopoDiv=7.432112654143253E-5  (2)
        class DEGRADATION IF : scoreRea=0.7071067811865476 ^ scoreProtTaxo=0.0 ^ scorePageRankTopoDiv=7.913623939731866E-5  (8)
        class DEGRADATION IF : scoreRea=0.8966167345234256 ^ scoreProtTaxo=0.0 ^ scorePageRankTopoDiv=7.191567364364338E-5  (1)
        class DEGRADATION IF : scoreRea=1.0 ^ scoreProtTaxo=13.050286539712438 ^ scorePageRankTopoDiv=7.175420992019046E-5  (3)
        class ENERGY IF : scoreRea=0.7071067811865476 ^ scoreProtTaxo=57.22088458559952 ^ scorePageRankTopoDiv=4.163608256714727E-4  (1)
        class ENERGY IF : scoreRea=1.2121830534626528 ^ scoreProtTaxo=0.858760746827019 ^ scorePageRankTopoDiv=4.0904708137729363E-4  (1)
        class ENERGY IF : scoreRea=1.8708286933869707 ^ scoreProtTaxo=291.22705834189765 ^ scorePageRankTopoDiv=3.676671633311061E-4  (2)
        class ENERGY IF : scoreRea=0.7071067811865476 ^ scoreProtTaxo=459.6016391037568 ^ scorePageRankTopoDiv=3.0829274756230974E-4  (2)
        class ENERGY IF : scoreRea=1.0 ^ scoreProtTaxo=101.09186523842465 ^ scorePageRankTopoDiv=3.147732888004335E-4  (4)
        class BIOSYNTHESIS IF : scoreRea=1.0 ^ 0.0<=scoreProtTaxo<=54.48256601886515 ^ 6.892763088378026E-4<=scorePageRankTopoDiv<=6.989120687488944E-4  (2)
        class DEGRADATION IF : scoreRea=0.1336306209562122 ^ scoreProtTaxo=0.0 ^ scorePageRankTopoDiv=1.4624678323885254E-5  (1)
        class DEGRADATION IF : scoreRea=0.07216878364870322 ^ scoreProtTaxo=2.460316855277241 ^ scorePageRankTopoDiv=1.816769296368919E-5  (2)
        class ENERGY IF : scoreRea=0.08006407690254357 ^ scoreProtTaxo=1.653721012550384 ^ scorePageRankTopoDiv=2.2280734529695785E-5  (1)
        class BIOSYNTHESIS IF : scoreRea=0.6324555320336759 ^ scoreProtTaxo=65.08449599386569 ^ scorePageRankTopoDiv=2.2907062577451448E-4  (5)
        class BIOSYNTHESIS IF : scoreRea=0.7071067811865476 ^ scoreProtTaxo=165.95993492398236 ^ scorePageRankTopoDiv=1.7817525502658317E-4  (2)
        class DEGRADATION IF : 1.0954451150103324<=scoreRea<=1.4142135623730951 ^ 608.5348962878501<=scoreProtTaxo<=639.1599181489809 ^ 1.5862456260021715E-
4<=scorePageRankTopoDiv<=2.6220409643941574E-4  (4)
        class BIOSYNTHESIS IF : scoreRea=0.408248290463863 ^ scoreProtTaxo=789.8485696478076 ^ scorePageRankTopoDiv=2.7322301369745626E-4  (5)
        class DEGRADATION IF : scoreRea=2.8284271247461903 ^ scoreProtTaxo=0.0 ^ scorePageRankTopoDiv=1.666222754058243E-4  (3)
        class DEGRADATION IF : scoreRea=1.8257418583505536 ^ scoreProtTaxo=98.4699452170974 ^ scorePageRankTopoDiv=7.104793139068288E-5  (3)
        class DEGRADATION IF : scoreRea=3.289913283319897 ^ scoreProtTaxo=559.0074476741592 ^ scorePageRankTopoDiv=3.0701005128886387E-4  (1)
        class DEGRADATION IF : scoreRea=0.7071067811865476 ^ 0.0<=scoreProtTaxo<=144.7478392962769 ^ 5.034282496324573E-4<=scorePageRankTopoDiv<=6.588344911766697E-4  (5)
        class DEGRADATION IF : scoreRea=1.0 ^ scoreProtTaxo=12.004669260881082 ^ scorePageRankTopoDiv=4.909937360556403E-4  (1)
        class DEGRADATION IF : scoreRea=1.4142135623730951 ^ scoreProtTaxo=41.60349216917426 ^ scorePageRankTopoDiv=5.21570208523604E-4  (2)
        class BIOSYNTHESIS IF : scoreRea=1.224744871391589 ^ scoreProtTaxo=56.161515770653565 ^ scorePageRankTopoDiv=6.948673131421873E-5  (4)
        class BIOSYNTHESIS IF : scoreRea=0.5 ^ scoreProtTaxo=0.0 ^ scorePageRankTopoDiv=5.698856430172402E-5  (2)
        class BIOSYNTHESIS IF : scoreRea=0.7071067811865476 ^ 0.0<=scoreProtTaxo<=2.0863620823036073 ^ scorePageRankTopoDiv=4.985531431087294E-4  (4)
        class DEGRADATION IF : 0.5773502691896257<=scoreRea<=0.7071067811865476 ^ 20.915987692451182<=scoreProtTaxo<=22.440204905652035 ^ 1.6686662865866955E-
4<=scorePageRankTopoDiv<=1.7408311085569377E-4  (6)
        class BIOSYNTHESIS IF : scoreRea=0.7071067811865476 ^ scoreProtTaxo=3.110805504795198 ^ scorePageRankTopoDiv=1.0511522598179651E-4  (3)
        class OTHER IF : scoreRea=0.3922322702763681 ^ scoreProtTaxo=0.0 ^ scorePageRankTopoDiv=2.7874776905481745E-5  (4)
        class BIOSYNTHESIS IF : scoreRea=0.31622776601683794 ^ scoreProtTaxo=387.6447516157186 ^ scorePageRankTopoDiv=8.360272329903238E-5  (2)
        class BIOSYNTHESIS IF : scoreRea=0.7071067811865476 ^ scoreProtTaxo=0.0 ^ 1.441783978103374E-4<=scorePageRankTopoDiv<=1.4791573811250174E-4  (9)
        class BIOSYNTHESIS IF : scoreRea=1.1547005383792515 ^ scoreProtTaxo=61.78620275022985 ^ scorePageRankTopoDiv=1.1737307636183124E-4  (1)
        class BIOSYNTHESIS IF : scoreRea=1.118033988749895 ^ scoreProtTaxo=0.0 ^ scorePageRankTopoDiv=5.1313350432075225E-5  (5)
        class BIOSYNTHESIS IF : scoreRea=0.655825835783953 ^ scoreProtTaxo=0.2566072677695416 ^ scorePageRankTopoDiv=1.0879989589336553E-4  (2)
        class BIOSYNTHESIS IF : scoreRea=1.4142135623730951 ^ scoreProtTaxo=0.0 ^ scorePageRankTopoDiv=3.160640423272123E-4  (3)
        class DEGRADATION IF : 1.1372040637927596<=scoreRea<=1.2029375808949572 ^ 1.2821315087821932<=scoreProtTaxo<=1.495970447499228 ^ 1.263409130347141E-
4<=scorePageRankTopoDiv<=1.3817645839686696E-4  (4)
        class BIOSYNTHESIS IF : scoreRea=1.118033988749895 ^ scoreProtTaxo=0.0 ^ scorePageRankTopoDiv=2.358103253919151E-4  (3)
```

```
        class BIOSYNTHESIS IF : scoreRea=0.7191949522280763 ^ scoreProtTaxo=55.61823899667411 ^ scorePageRankTopoDiv=1.3556204474833197E-5  (2)
        class BIOSYNTHESIS IF : scoreRea=0.1336306209562122 ^ scoreProtTaxo=113.06193486507043 ^ scorePageRankTopoDiv=8.526134756584441E-5  (3)
        class DEGRADATION IF : scoreRea=2.680951323690902 ^ scoreProtTaxo=0.011672391865901513 ^ scorePageRankTopoDiv=2.1883160997492087E-4  (4)
        class BIOSYNTHESIS IF : scoreRea=1.0 ^ scoreProtTaxo=0.0 ^ scorePageRankTopoDiv=1.9055649310582596E-4  (2)
        class BIOSYNTHESIS IF : scoreRea=1.0 ^ scoreProtTaxo=0.0 ^ scorePageRankTopoDiv=1.8860851388601528E-4  (1)
        class DEGRADATION IF : 1.0<=scoreRea<=1.1881770515720091 ^ 4.7879252966052475<=scoreProtTaxo<=5.411851401620706 ^ 1.1442075717380128E-
4<=scorePageRankTopoDiv<=1.2181717835020451E-4  (4)
        class ENERGY IF : scoreRea=3.4641016151377544 ^ scoreProtTaxo=16.560296819002364 ^ scorePageRankTopoDiv=2.120396524500385E-4  (3)
        class DEGRADATION IF : scoreRea=1.0 ^ scoreProtTaxo=0.0 ^ scorePageRankTopoDiv=1.1328728240243136E-4  (4)
        class ENERGY IF : scoreRea=1.0 ^ scoreProtTaxo=21.7568033167796 ^ scorePageRankTopoDiv=7.011720372293434E-4  (4)
        class BIOSYNTHESIS IF : scoreRea=1.0 ^ scoreProtTaxo=0.0 ^ scorePageRankTopoDiv=3.3064912264776746E-4  (1)
        class BIOSYNTHESIS IF : scoreRea=1.1114378604524227 ^ scoreProtTaxo=19.8045615400731 ^ scorePageRankTopoDiv=5.409945344885199E-5  (1)
        class DEGRADATION IF : scoreRea=5.372405894758811 ^ scoreProtTaxo=64.86872458326154 ^ scorePageRankTopoDiv=2.5451465611213983E-4  (1)
        class BIOSYNTHESIS IF : scoreRea=0.9274777915203366 ^ scoreProtTaxo=2.284447209188501 ^ scorePageRankTopoDiv=1.341109037928679E-4  (1)
        class BIOSYNTHESIS IF : scoreRea=0.28867513459481287 ^ scoreProtTaxo=1.8435289494404254 ^ scorePageRankTopoDiv=5.057229096274693E-5  (1)
        class DEGRADATION IF : scoreRea=7.874007874011811 ^ scoreProtTaxo=83.60493888793984 ^ scorePageRankTopoDiv=9.184702952142395E-5  (4)
        class BIOSYNTHESIS IF : scoreRea=0.7071067811865476 ^ scoreProtTaxo=488.5222615194924 ^ scorePageRankTopoDiv=2.954382058651906E-4  (1)
        class BIOSYNTHESIS IF : scoreRea=1.0 ^ scoreProtTaxo=99.94050773418287 ^ scorePageRankTopoDiv=5.84516351945006E-4  (5)
        class OTHER IF : scoreRea=2.0 ^ scoreProtTaxo=0.0 ^ scorePageRankTopoDiv=4.088733957652656E-4  (2)
        class OTHER IF : scoreRea=0.20965696734438366 ^ scoreProtTaxo=62.006127251263905 ^ scorePageRankTopoDiv=3.6903530145216174E-5  (1)
        class BIOSYNTHESIS IF : scoreRea=1.0 ^ scoreProtTaxo=0.0 ^ scorePageRankTopoDiv=1.9202370889358065E-4  (1)
        class BIOSYNTHESIS IF : scoreRea=2.8535691936340255 ^ scoreProtTaxo=0.0 ^ scorePageRankTopoDiv=3.201551503233059E-4  (2)
        class BIOSYNTHESIS IF : scoreRea=1.0 ^ scoreProtTaxo=0.0 ^ scorePageRankTopoDiv=1.9202370889358065E-4  (1)
        class DEGRADATION IF : scoreRea=1.4142135623730951 ^ scoreProtTaxo=1121.1691346845957 ^ scorePageRankTopoDiv=1.50642091339147E-4  (2)
        class BIOSYNTHESIS IF : scoreRea=0.5 ^ scoreProtTaxo=0.0 ^ scorePageRankTopoDiv=4.787860914058206E-5  (3)
        class OTHER IF : scoreRea=2.0 ^ scoreProtTaxo=57.65446242084278 ^ scorePageRankTopoDiv=4.287988675749166E-4  (3)
        class DEGRADATION IF : scoreRea=0.7071067811865476 ^ scoreProtTaxo=273.33252297887157 ^ scorePageRankTopoDiv=4.474941279021902E-5  (1)
        class OTHER IF : scoreRea=0.8498365855987975 ^ scoreProtTaxo=491.14396928120954 ^ scorePageRankTopoDiv=8.933471786716984E-5  (3)

Stat :
        class DEGRADATION : 315 exemplar(s) including 288 Hyperrectangle(s) and 27 Single(s).
        class BIOSYNTHESIS : 455 exemplar(s) including 385 Hyperrectangle(s) and 70 Single(s).
        class OTHER : 92 exemplar(s) including 80 Hyperrectangle(s) and 12 Single(s).
        class DETOX : 35 exemplar(s) including 31 Hyperrectangle(s) and 4 Single(s).
        class ENERGY : 71 exemplar(s) including 64 Hyperrectangle(s) and 7 Single(s).

        Total : 968 exemplars(s) including 848 Hyperrectangle(s) and 120 Single(s).

        Feature weights : [0.026621704589354037 0.013098001491379322 0.03430947381803635]



Time taken to build model: 1.72 seconds

=== Stratified cross-validation ===
=== Summary ===

Correctly Classified Instances        7822               94.7432 %
Incorrectly Classified Instances       434                5.2568 %
Kappa statistic                          0.9076
Mean absolute error                      0.021
Root mean squared error                  0.145
Relative absolute error                  9.2047 %
Root relative squared error             42.9119 %
Total Number of Instances             8256

=== Detailed Accuracy By Class ===

               TP Rate   FP Rate   Precision   Recall  F-Measure   ROC Area  Class
                 0.922     0.028      0.927     0.922     0.925      0.947    DEGRADATION
                 0.965     0.06       0.958     0.965     0.961      0.952    BIOSYNTHESIS
                 0.929     0.003      0.947     0.929     0.938      0.963    OTHER
                 0.869     0.001      0.926     0.869     0.897      0.934    DETOX
                 0.935     0.004      0.939     0.935     0.937      0.966    ENERGY
Weighted Avg.    0.947     0.043      0.947     0.947     0.947      0.952

=== Confusion Matrix ===
```

| a | b | c | d | e | <-- classified as |
|---|---|---|---|---|---|
| 2121 | 151 | 10 | 3 | 15 | a = DEGRADATION |
| 136 | 4672 | 16 | 6 | 13 | b = BIOSYNTHESIS |
| 13 | 22 | 469 | 0 | 1 | c = OTHER |
| 6 | 11 | 0 | 113 | 0 | d = DETOX |
| 11 | 20 | 0 | 0 | 447 | e = ENERGY |
